# Synthesis of Bicyclic Hemiacetals Catalyzed by Unnatural Densely Substituted γ-Dipeptides

**DOI:** 10.1021/acs.joc.2c01230

**Published:** 2022-09-30

**Authors:** Maddalen Agirre, Tamara Bello, Jinxiu Zhou, María de Gracia Retamosa, Fernando P. Cossío

**Affiliations:** † Departamento de Química Orgánica I, University of the Basque Country (UPV/EHU), P° Manuel Lardizabal 3, 20018 Donostia-San Sebastián, Spain; ‡ CIC Energigune, Parque Tecnológico de Álava, 01510 Vitoria-Gasteiz, Spain; § Quimatryx Ltd., Parque Tecnológico de Gipuzkoa, 2009 Donostia-San Sebastián, Spain; ∥ Department of Polymer Science and Technology, Institute of Polymer Materials, University of the Basque Country (UPV/EHU), P° Manuel Lardizabal 3, 20018 Donostia-San Sebastián, Spain; ⊥ Instituto de Síntesis Orgánica y Departamento de Química Orgánica, Universidad de Alicante 03080 Alicante, Spain; # Centro de Innovación en Química Avanzada (ORFEO-CINQA), https://orfeocinqa.es/; @ Donostia International Physics Center (DIPC), P° Manuel Lardizabal 4, 20018 Donostia-San Sebastián, Spain

## Abstract

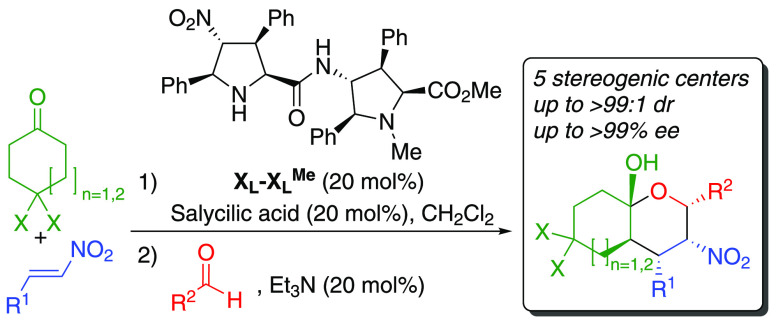

The asymmetric synthesis of bicyclic highly substituted tetrahydropyrans is described. The reaction is catalyzed by unnatural γ-dipeptides based on densely substituted l- and d-proline derivatives. This organocatalytic one-pot reaction takes place among a ketone, a nitroalkene, and an aldehyde to yield an octahydro-2*H*-chromene scaffold. Monomeric species, from which the corresponding γ-dipeptides are synthesized, cannot catalyze the reaction, thus confirming the emergent nature of the catalytic behavior of these dimeric species.

Tetrahydropyrans (THPs) are important structural six-membered oxygenated heterocycles that incorporate up to five stereogenic centers. In particular, fused THPs, including one or two octahydro-8a*H*-chromen-8a-ol units, are found in natural products. For instance, Figure 1 includes compounds **A**,^1^**B**,^2^**C**, and **D**.^3,4^ In addition, some of them are biologically active, such as diterpene **C**, which inhibits androgen receptor transcriptional activity in prostate cancer cells.^5^

**Figure 1:**
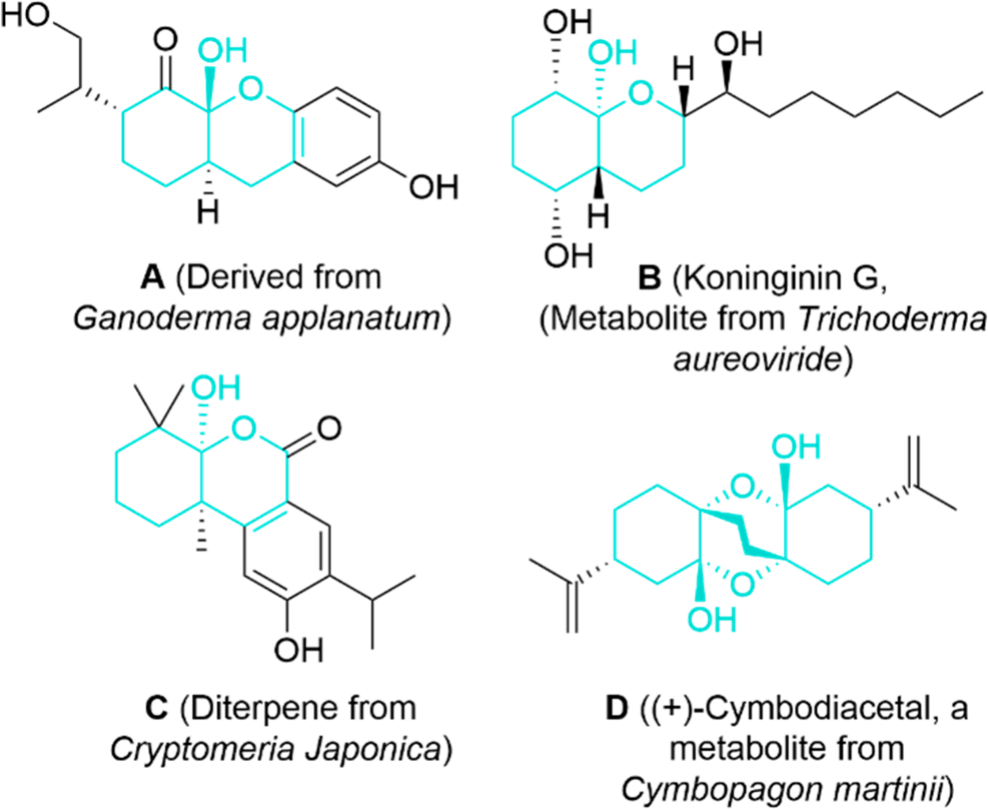
Several examples of natural products containing one or two octahydro-8a*H*-chromen-8a-ol units (colored cyan).

Several methods have been developed for the construction of chiral THP structures,^6,7^ among which asymmetric organocatalysis deserves special attention.^8^ Cordova’s group reported in 2005 the first organocatalytic synthesis of THPs employing iterative aldol reactions.^9^ Enders et al. also published an interesting work on the organocatalytic synthesis of monocyclic 3-acetyl-2-hydroxy-5-nitro-THPs.^10^ It is noteworthy that there is only one example of the synthesis of cyclohexane-fused 2-unsubstituted THPs by using cyclohexanone and 2-nitroprop-2-en-1-ols in a two-component hemiketalization reaction.^11^ In addition, the Michael–Henry–hemiketalization reaction has scarcely been investigated. It was not until 2011 that Hayashi and co-workers described a one-pot Michael–Henry–acetalization starting from nitroalkenes and two different aldehydes to yield mixtures of α- and β-anomeric monocyclic THPs.^12^ In 2015, an organocatalytic kinetic resolution of racemic secondary nitroallylic alcohols via a Michael–hemiketalization sequence was reported to give densely substituted monocyclic tetrahydropyran-6-ols.^13^ However, to the best of our knowledge, no Michael–Henry–hemiketalization reaction for the synthesis of fused THPs has been described in the literature.^14^

We have previously described the synthesis via (3+2) cycloadditions of densely substituted proline esters and their abilities as organocatalysts.^15^ We found that unnatural l-*exo*-4-amino 2-carboxy O_2_N-X_L_ species [**1** (Figure 2)] are efficient catalysts for aldol reactions^16^ and for a particular three-component cyclization.^17^ In contrast, compounds of type **1** were unable to catalyze conjugate additions between ketones and nitroalkenes. However, amino derivatives H_2_N-X_L_**2** (Figure 2) proved to be suitable organocatalysts for both aldol and Michael reactions.^18^ More recently, we have found that γ-dipeptides **3–5** possessing 4-nitro terminal groups exhibit catalytic properties, which can promote the three previously commented reactions with excellent yields and stereoselectivities.^19^ On the basis of DFT calculations, we interpreted these results in terms of a favorable combination between the enamine catalytic site of one unit of the dipeptide (HOMO-enhancing effect on the ketone) and the protonated pyrrolidine unit of the other component (LUMO-lowering effect on the Michael acceptor).

**Figure 2:**
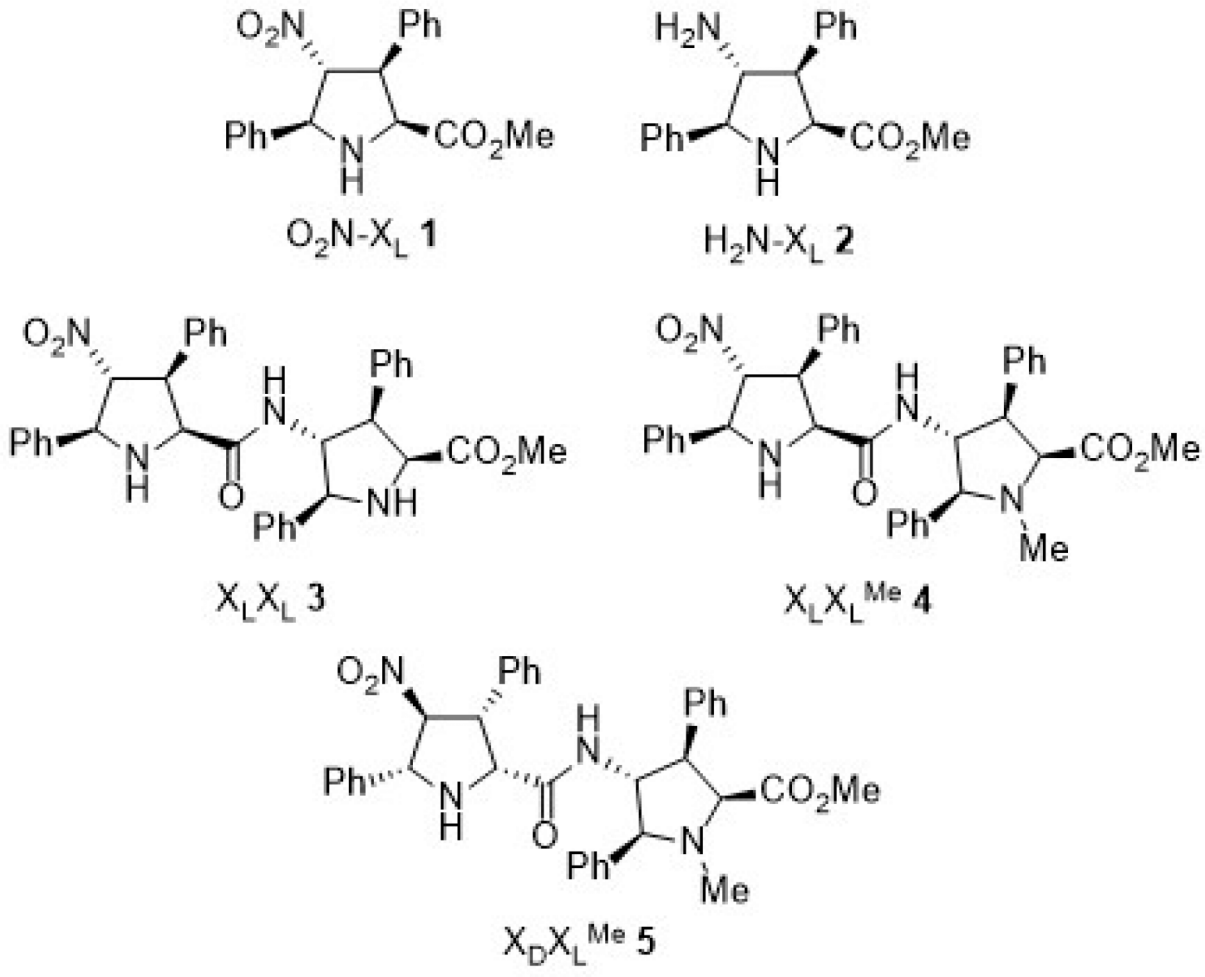
Monomeric (**1** and **2**) and dimeric (**3–5**) unnatural proline organocatalysts used in this work.

In view of these precedents, we envisioned the possible chemical synthesis of fused THP octahydro-2*H*-chromene scaffolds by the Michael–Henry–hemiketalization one-pot reaction starting from ketones, nitroalkenes, and aldehydes, as shown in Table 1. Initially, the three starting reagents were mixed together to yield traces of the aldol product corresponding to the competitive aldol reaction between cyclohexanone **6a** and ethyl glyoxylate **8a**. Monomeric pyrrolidines **1** (O_2_N-X_L_) and **2** (H_2_N-X_L_) were inactive for this reaction (Table 1, entries 1 and 2, respectively). In contrast, the reaction promoted by dipeptide **3** (X_L_X_L_) showed reasonable reaction times for the conjugate addition. In addition, the Henry–hemiketalization step was completed within 6 h, and the final product was obtained in good yield, good diastereomeric ratio, and excellent enantiomeric excess (entry 3). Increasing the catalytic load and using 4-nitrobenzoic acid accelerated the conjugate addition step, but a loss of diastereoselectivity was observed (entry 4). It is interesting to note that partial loss of selectivity after completion of the reaction in the presence of acetic acid has been reported.^20^ These results were improved in terms of ee when dimeric catalyst **4** (X_L_-X_L_^Me^), with only one active enamine precursor catalytic site, was employed (entry 5). Inversion of configuration in the pyrrolidine ring that possesses the NH group required for the formation of the enamine nucleophile (catalyst **5**, X_D_-X_L_^Me^) resulted in longer reaction times, and despite the good diastereomeric ratio, the yield and ee values decreased significantly, with inversion of configuration in final product **10aaa**. This latter result is compatible with a Michael step that determines the final stereochemical outcome and a partial mismatching between both d- and l-pyrrolidine units, thus resulting in a lower ee. Increasing the catalytic load to 20 mol % allowed us to obtain THP adduct **10aaa** as the sole product in good yield and excellent enantiomeric excess (entry 7). Final experiments comprised the study of the effect of the base in the reaction. None of the tested basic additives could improve the results obtained with Et_3_N (entry 7). Treatment with DIPEA and DABCO entailed long cyclization times (entries 8 and 11, respectively), and the use of DBU resulted in two diastereomeric species (entry 9, vide infra for further details). When 1 equiv of DBU was used, the other diastereomeric species was isolated with 65% yield and 98% ee (entry 10). The structure and stereochemistry of both kinetically and thermodynamically favored products (**10aaa** and **10aaa′**, vide infra) were confirmed by X-ray diffraction analysis (see the Supporting Information). Interestingly, neither proline and several derivatives nor diamine organocatalysts could sufficiently promote this reaction efficiently (see the Supporting Information for further details).

**Table 1:**
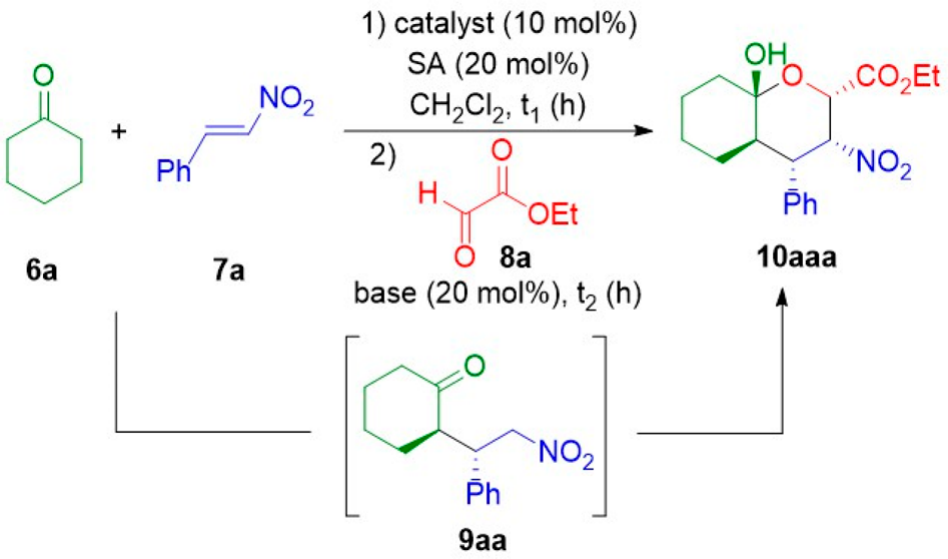
Screening of Various Densely Substituted Prolines and Bases for the Optimization of the Michael–Henry–Hemiketalization Reaction^a^,^b^

entry	catalyst	base	*t*_1_ (h)	*t*_2_ (h)	dr^c^	yield^d^ (%)	ee^e^ (%)
1	**1** (O_2_N-X_L_)	Et_3_N	48	240	nd	<5	nd
2^f^	**2** (H_2_N-X_L_)	Et_3_N	48	216	nd	<5	nd
3	**3** (X_L_X_L_)	Et_3_N	48	6	85:15	73	93
4^g^	**3** (X_L_X_L_)	Et_3_N	16	6	75:25	70	91
5	**4** (X_L_X_L_^Me^)	Et_3_N	24	6	80:20	47	99
6	**5** (X_D_X_L_^Me^)	Et_3_N	144	6	84:16	69	–63
7^g^	**4** (X_L_X_L_^Me^)	Et_3_N	24	6	>99:1	72	99
8^g^	**4** (X_L_X_L_^Me^)	DIPEA	24	168	nd	<5	nd
9^g^	**4** (X_L_X_L_^Me^)	DBU	24	48	50:50	53^h^	96
10^g^	**4** (X_L_X_L_^Me^)	DBU^i^	24	48	<1:99	65	98
11^g^	**4** (X_L_X_L_^Me^)	DABCO	24	48	nd	30	65

a The first step was conducted using ketone **6a** (1.0 mmol) and *trans*-β-nitrostyrene **7a** (1.1 mmol) in the presence of 10 mol % catalyst and 20 mol % salicylic acid (SA). The second step was conducted using aldehyde **8a** (2.0 mmol) and 20 mol % Et_3_N. nd denotes not determined.

b Reactions were monitored by ^1^H NMR, and mixtures were stirred at room temperature until the starting materials were totally consumed (>99% conversion).

c dr refers to (2*S*,3*R*,4*S*,4a*R*,8a*S*)-**10aaa**:(2*S*,3*S*,4*S*,4a*R*,8a*S*)-**10aaa′** ratios (different configurations of the chiral centers in the α-position with respect to the nitro group) and determined by ^1^H NMR analysis of the crude mixture.

d Isolated yield, after purification by column chromatography of **10aaa**.

e Determined by HPLC with a chiral stationary phase.

f With 30 mol % catalyst and 30 mol % *p*-nitrobenzoic acid.

g With 20 mol % catalyst.

h Yields refer to the sum of both diastereomers.

i With 1 equiv of DBU.

Having determined the best reaction conditions, we investigated the scope of this process by evaluating several nitroalkenes while keeping the other two components **6a** and **8a** constant. The reaction proceeded smoothly in the presence of dimeric catalyst **4** with aromatic and heteroaromatic nitroalkenes **7a–h** (Scheme 1). While aliphatic conjugated nitroalkene **7i** was also convenient for this transformation yielding adduct **10aia** in moderate yield and excellent ee, aliphatic nitroalkene (*E*)-(2-nitrovinyl)cyclohexane **7j** provided only the intermediate Michael adduct in low conversion (<20%) after reaction for 7 days.

**Scheme 1:**
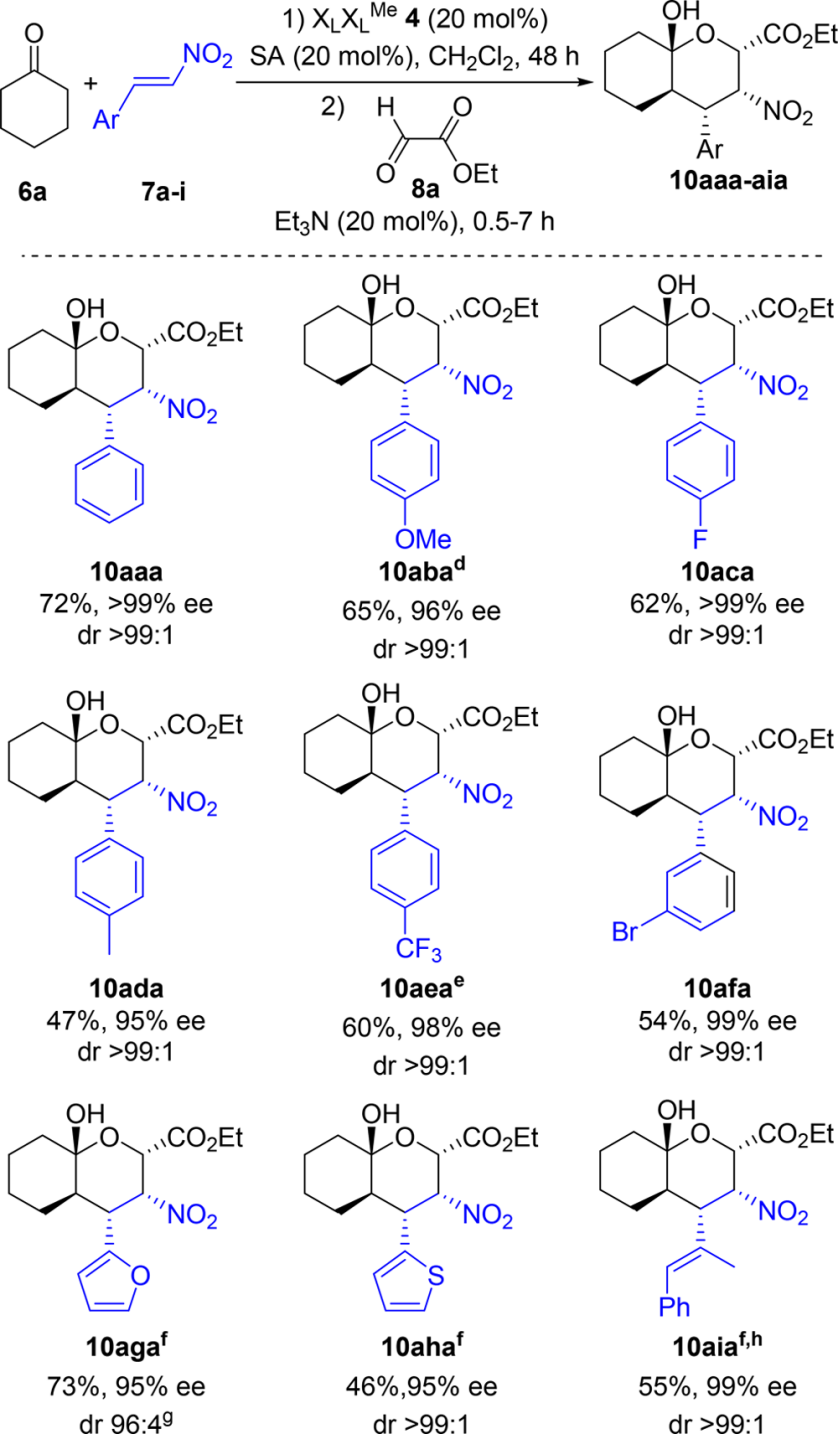
Enantioselective Michael–Henry–Hemiketalization Reaction with Cyclohexanone, Ethyl Glyoxylate, and Various Nitroalkenes, Catalyzed by Dimeric Pyrrolidine X_L_X_L_^Me^– The first step was conducted using ketone **6a** (1.0 mmol) and the corresponding *trans*-β-nitroalkene **7a–i** (1.1 mmol) in the presence of 10 mol % O_2_N-X_L_-X_L_^Me^-OMe **4** and 20 mol % salicylic acid, at room temperature for 48 h. The second step was conducted using aldehyde **8a** (2.0 mmol) and 20 mol % Et_3_N and monitored by ^1^H NMR until the starting materials were totally consumed (>99% conversion). Yields refer to isolated products. ee determined by HPLC with a chiral stationary phase corresponding to major enantiomer (2*S*,3*R*,4*S*,4a*R*,8a*S*)-**10aaa–aia**. The Michael reaction required 3 days. Performed in toluene. The second step was performed at 0 °C. The product was obtained as an inseparable mixture of diastereomers. The Michael reaction required 6 days.

DFT calculations on the **6a** + **7a** + **8a** → **10aaa** reaction in the presence of NMe_3_ and SA (see the Supporting Information) provided a suitable model of the sequence of events that leads to the final product from the nitronate derived from intermediate Michael adduct **9aa**. The stereochemistry of this intermediate is determined by the chiral organocatalyst. In turn, the facial discrimination of aldehyde **8a** stems from this intermediate. From these calculations (see Figure S1), we concluded that the role of the base and the acidic additive is essential for determining the viability of the reaction and its stereochemistry.

The reaction also worked with other cyclic ketones such as cycloheptanone **6b** and 1,4-cyclohexanedione monoethylene acetal **6c**. However, small changes were necessary to obtain the corresponding cycloadducts (Scheme 2). Derivative **10baa** demanded equimolar amounts of Et_3_N for the total consumption of the γ-nitroketone intermediate. The corresponding adduct **10baa** was obtained in moderate yield and high enantioselectivity, but in a 92:8 mixture of inseparable diastereomers. The synthesis of **10caa** required 7 days for the Michael step to reach full conversion. Attempts to shorten the reaction time by increasing the temperature to 45 °C resulted in the formation of sluggish mixtures. The following Henry–hemiketalization step, on the contrary, was completed in 1 h. The desired THP derivative **10caa** was obtained as a single diastereomer in 62% yield and 87% ee. Unfortunately, when tetrahydro-4*H*-pyran-4-one **6d** and cyclohexane-1,3-dione **6e** were used as starting materials, no formation of the corresponding Michael adducts was observed in the presence of salicylic acid or TFA. In contrast, when cyclopentanone **6f** was employed, the final tetrahydropyran derivative could not be isolated due low conversion and selectivity (see the Supporting Information).

**Scheme 2:**
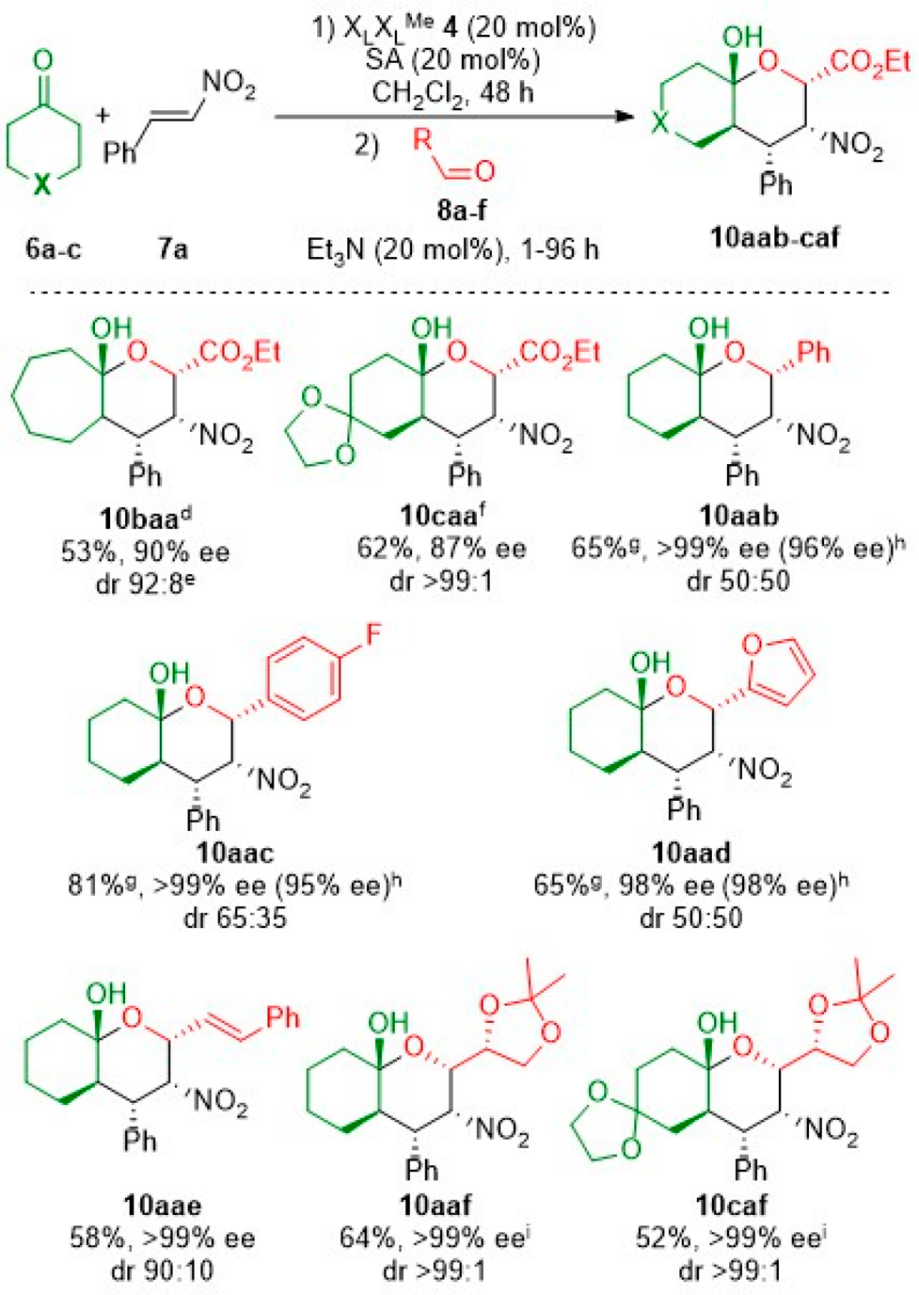
Enantioselective Michael–Henry–Hemiketalization Reaction with Different Ketones **6** and Aldehydes **8**– The first step was conducted using the corresponding ketone **6a–c** (1.0 mmol) and *trans*-β-nitrostyrene **7a** (1.1 mmol) in the presence of 10 mol % O_2_N-X_L_-X_L_^Me^-OMe **4** and 20 mol % salicylic acid, at room temperature for 48 h. The second step was conducted using the corresponding aldehyde **8a–f** (2.0 mmol) and 20 mol % Et_3_N and monitored by ^1^H NMR until the starting materials were totally consumed (>99% conversion). Yields refer to isolated products. Enantiomeric excesses determined by HPLC with a chiral stationary phase corresponding to major enantiomer (2*S*,3*R*,4*S*,4a*R*,8a*S*)-**10aab–caf**. The first step was performed with trifluoroacetic acid as an additive, and the second step was conducted using 1 equiv of Et_3_N. The product was obtained as an inseparable mixture of diastereomers. Diastereomeric ratio related to the cyclization of *syn*- and *anti*-Michael adducts. The first step required 7 days. Yields refer to the sum of both diastereomers. ee refers to (2*S*,3*S*,4*S*,4a*R*,8a*S*)-**10aab′–caf′** enantiomers. ee refers to de (diastereomeric excess) measured by ^1^H NMR.

The Henry reaction step was found to be compatible with aromatic and aliphatic conjugated aldehydes (Scheme 2). Because the studied aldehydes were less electrophilic than model ethyl glyoxylate **8a**, in some cases changes in the number of equivalents of the aldehyde and triethylamine were required (see the Supporting Information for further details). The reaction could be applied to a broad scope of aldehydes to generate the corresponding THP derivatives in good overall yields and excellent enantioselectivities. Nevertheless, the diasteroselectivity of the process fluctuated from low to excellent depending on the aldehyde employed. Heteroaromatic aldehydes such as furfural could also be applied to this one-pot reaction with 1 equiv of triethylamine. The final products were obtained as a 50:50 mixture of diastereomers, in good overall yield and excellent enantioselectivities. Because problems arose in the purification of the final products, isomerization of **10aad** into **10aad′** was investigated (*vide infra*). This method was extended to chiral aldehyde **8f** aldehyde, which would act as a chiral auxiliary leading to the formation of a single diastereomer. Indeed, final product **10aaf** was obtained in good yield and excellent diastereo- and enantioselectivity. Finally, cyclohexanone **6a** was replaced by 1,4-cyclohexanedione monoethylene acetal **6c** to generate the more complex THP derivative **10caf** in moderate yield and with virtually complete stereocontrol.

The isomerization of final products **10aad** and **10aad′** (see also entries 9 and 10, respectively, of Table 1) was studied. Treatment with DBU (1 equiv) at room temperature for 16 h led to **10aad′** quantitatively (Scheme 3a). This reaction was successfully scaled up to 1 mmol without losing the reaction efficiency. Hence, in the one-pot process for the straight synthesis of epimer **10aad′**, the second step was conducted using 1 equiv of DBU. Under these conditions, the desired product was obtained in good yield with excellent diastereo- and enantiocontrol (Scheme 3b).

**Scheme 3:**
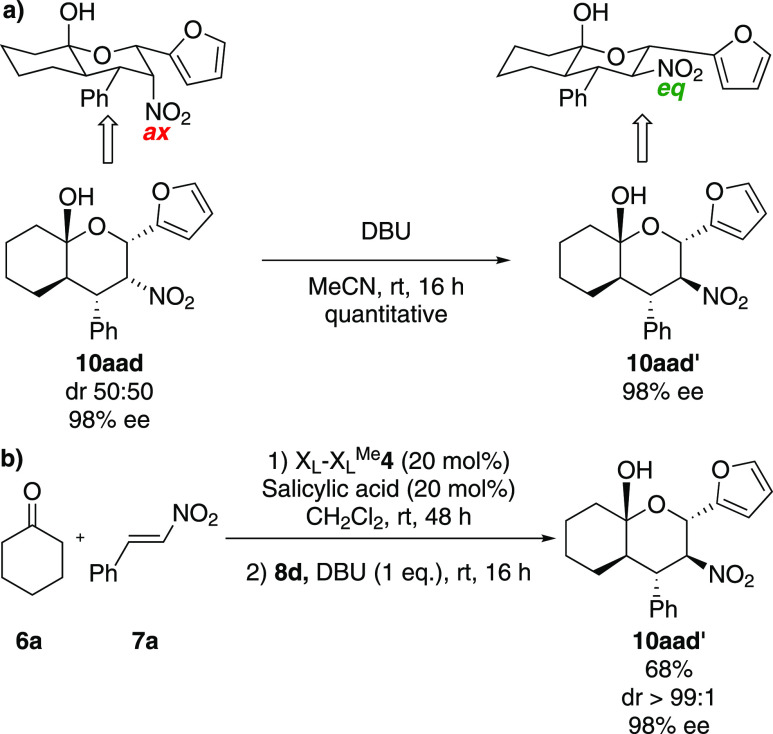
(a) Isomerization Reaction for the Formation of Derivative **10aad′** and (b) One-Pot Synthesis of Compound **10aad′** with 1 equiv of DBU

This isomerization is compatible with the change in configuration of the carbon atom contiguous to the nitro group, which passes from an axial position in **10aad** to a thermodynamically favored equatorial geometry in **10aad′** (see Scheme 3 and the Supporting Information for additional DFT calculations).

In summary, in this study, we have found that one-pot Michael–Henry–hemiketalization reaction of ketones catalyzed by dimeric γ-peptides **3–5** leads to bicyclic densely substituted octahydro-2*H*-chromene derivatives with up to five chiral centers in good yields and excellent distereo- and enantioselectivities. This one-pot process constitutes an example of distinct catalytic properties on passing from monomeric to condensed dimeric species.

## Supporting Information Available

General experimental procedures, NMR spectra for all compounds and full characterization of the described products, and computational data, including energies, harmonic analysis, and Cartesian coordinates, of all relevant stationary points (PDF)
