# Supplementary material for: Synthesis of Bicyclic Hemiacetals Catalyzed by Unnatural Densely Substituted γ-Dipeptides
Source: J Org Chem. 2022 Sep 30;87(21):14819–24. doi: 10.1021/acs.joc.2c01230 (PMC9639056; doi:10.1021/acs.joc.2c01230)
Supplement: Supplementary file 1 — jo2c01230_si_001.pdf [file jo2c01230_si_001.pdf]

Synthesis of Bicyclic Hemiacetals Catalyzed by Unnatural Densely Substituted γ-Dipeptides

Maddalen Agirre,[‡,†,§] Tamara Bello,[‡,†,#] Jinxiu Zhou,[†,√] María de Gracia Retamosa,[^,¬,◊,*] and Fernando P. Cossío[†,¬,◊,*]

[†]Departamento de Química Orgánica I, University of the Basque Country (UPV/EHU), Pº Manuel Lardizabal 3, 20018 Donostia/San Sebastián, Spain.
[§]CIC Energigune, Parque Tecnológico de Álava, 01510, Vitoria-Gasteiz, Spain.
[#]Quimatryx Ltd. Parque Tecnológico de Gipuzkoa, 2009 San Sebastián-Donostia, Spain.
[√]Department of Polymer Science and Technology, Institute of Polymer Materials, University of the Basque Country UPV/EHU, Pº Manuel Lardizabal 3, 20018 Donostia/San Sebastian, Spain.
[^]Instituto de Síntesis Orgánica y Departamento de Química Orgánica, Universidad de Alicante 03080-Alicante, Spain.
[¬]Centro de Innovación en Química Avanzada (ORFEO-CINQA), Spain
[◊]Donostia International Physics Center (DIPC), Pº Manuel Lardizabal 4, 20018 Donostia/San Sebastián, Spain.

[‡]These authors contributed equally to this work.

Supporting Information

TABLE OF CONTENTS

1 General Remarks S3

2 Procedure for the synthesis of NO_2-X_L-Gly-OMe **14** organocatalyst S4

3 Screening of different organocatalysts based on proline derivatives for the Michael Addition Reaction S4

4 General Procedure for the One-pot Michael-Henry-Acetalization Reaction S6

4.1 Screening of different chiral organocatalysts based on primary amines S6

4.2 Procedure for the synthesis of **10aaa** at the 5 mmol scale S7

4.3 Isomerization Reaction for the Synthesis of **10aad’** S7

4.4 Studies with Other Cyclic Ketones S8

5 NMR spectra S28

6 X-Ray diffraction structures S57

6.1 X-Ray diffraction of **10aaa** (CCDC 2090677) S57

6.2 X-Ray diffraction of **10aaa’** (CCDC 2090834) S59

7 Computational studies S60

7.1 Michael-Henry-Hemiketalization Reaction S60

7.2 Isomerization Reaction S62

8 References S80

1 General Remarks

Unless otherwise noted, reagents (**6**, **7** and **8**) and organocatalysts **11-13, 15** and **16** were purchased from commercial suppliers. Nitrodiene **7i**[1] and aldehyde **8f**[2] were prepared according to literature. Catalysts NO_2-X_L-**1**, NH_2-X_L-**2**, X_LX_L-**3**, $X_LX_L^{Me}$-**4**, $X_DX_L^{Me}$-**5**, were prepared following our previously described procedures. [3,4,5,6]

TLC was performed on 0.25mm silica gel 60 F254 aluminum plates and visualized with UV lamps or potassium permanganate stain. Flash column chromatography was carried out on columns of silica gel 60 (particle size 23-40 μm).

Optical rotations were measured at 589 nm (Sodium line) in a digital polarimeter with a thermally jacketed 5 cm cell at approximately 20 °C. Concentrations are given in g/100 mL.

Infrared spectra were recorded on an Alpha-Bruker FT-IR spectrometer with a single reflection ATR module. Wavenumbers are given in cm^{-1}.

High Resolution Mass Spectra (HRMS) analyses were carried out by SGIker services (Central Service of Alava and Bizkaia, University of the Basque Country) and performed on a LC/QTOF, Agilent mass spectrometer using electrospray ionization (ESI) mode.

NMR spectra were recorded at 400 or 500 MHz for ^{1}H NMR, 101 or 126 MHz for ^{13}C NMR and 376 MHz for ^{19}F NMR using $CDCl_3$, acetone-*d6* and methanol-*d4* as solvents and TMS as internal standard. The data are reported as s = singlet, d = doublet, t = triplet, q = quartet, m = multiplet or unresolved, bs = broad signal, coupling constant(s) (*J*) in Hz, integration. ^{13}C NMR spectra were recorded with ^{1}H decoupling. Structural assignments were made with additional information from gCOSY, gHSQC, and gHMBC experiments

Enantioselectivities were measured by HPLC using chiral stationary phases (Daicel Chiralpak IA/IB/IC/ID). In these experiments the racemic mixtures were analysed in order to establish the enantiomeric parameters of each enantiomer.

For X-Ray diffraction analyses, Agilent Technologies Super-Nova diffractometer was employed, equipped with monochromated Cu kα radiation (λ = 1.54184 Å) and Atlas CCD detector. Measurements were accomplished at 100 K with the aid of an Oxford Cryostream 700 PLUS temperature device. Data frames were processed (united cell determination, analytical absorption correction with face indexing, intensity data integration and correction for Lorentz and polarization effects) utilizing the Crysalis software package.[7] The structure was solved by Superflip[8] and refined by full-matrix least-squares with SHELXL-97[9]. Final

geometrical calculations were carried out on Mercury[10] and PLATON[11] as integrated in WinGX[12]. Samples were prepared by heating a solution of the compound in a xx yy, followed by slow evaporation.

2 Procedure for the synthesis of NO_2-X_L-Gly-OMe 14 organocatalyst

Catalysts NO_2-X_L-Gly-OMe **14** was prepared following our previously described procedures[3,4,5,6]: To a stirred solution of the corresponding amine (0.8 mmol) in CH_2Cl_2 (10 mL) was added acid (1.0 mmol), PyBOP (1.0 mmol) and by diisopropyl ethyl amine (1.4 mmol). The resulting mixture was then stirred until completion of the reaction. Then, the reaction mixture was diluted with CH_2Cl_2, washed with a 1M HCl solution, saturated aqueous $NaHCO_3$, brine and then dried over Na_2SO_4. Evaporation of the solvent followed by column chromatography eluting with EtOAc/hexane provided the product described below.

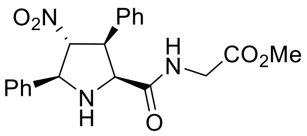

Methyl 2-((2S,3S,4R,5S)-4-nitro-3,5-diphenylpyrrolidine-2-carboxamido)acetate (NO_2-X_L-Gly-OMe **14)**

Yield: 72 mg, 85%, yellow syrup; **$[\alpha]_D^{25}$** = +86.07 (*c* 1.15, acetone); **FTIR** (neat, cm^{-1}): 1744, 1666, 1546, 1208, 698; **^{1}H NMR** (500 MHz, $CDCl_3$) δ 7.57 (d, *J* = 7.3 Hz, 2H, ArH), 7.46 (dd, *J* = 21.7, 14.7 Hz, 2H, ArH), 7.39 (dd, *J* = 16.2, 9.0 Hz, 1H, ArH), 7.31 – 7.23 (m, 3H, ArH), 7.23 – 7.18 (m, 2H, ArH), 7.01 (m, 1H, CONH), 5.16 (t, *J* = 7.7 Hz, 1H, C^4H), 4.85 (d, *J* = 7.9 Hz, 1H, C^5H), 4.44 (d, *J* = 9.5 Hz, 1H, C^2H), 4.41 – 4.32 (dd, *J* = 9.4, 7.6 Hz, 1H, C^3H), 3.73 (dd, *J* = 18.3, 5.2 Hz, 1H, CH_2), 3.72 (s, 3H, CO_2Me), 3.61 (dd, *J* = 18.5, 5.0 Hz, 1H, CH_2); **^{13}C NMR** (101 MHz, $CDCl_3$) δ 170.2, 170.0, 137.7, 135.7, 129.2, 129.1, 128.6, 128.4, 128.2, 126.8, 95.2, 66.6, 64.5, 53.1, 52.4, 40.6; **HRMS** (ESI) m/z: $[M + H]^+$ Calcd for $C_{20}H_{22}N_3O_5$ 384.1559. Found: 384.1567.

3 Screening of different organocatalysts based on proline derivatives for the Michael Addition Reaction

Based on previous studies of our research group[6] we evaluated different proline derivative organocatalyts in the Michael reaction between cyclohexanone **6a** and nitrostyrene **7a** in the presence of benzoic acid as additive. The obtained results are shown in Table S1. Reactions were performed under neat conditions using cyclohexanone **6a** (0.8 mmol), trans-β-nitrostyrene **7a** (0.1 mmol) in presence of the corresponding catalyst (0.03 mmol) and benzoic acid (0.03 mmol).

Under these conditions, L-proline **11** was not very efficient and a low conversion was observed after one day of reaction (Table S1, entry 1). It is interesting to note that Enders and Seki[13] observed, under different conditions, medium-low ee's in the Michael reaction between **6a** and **7a** (18-57%). Similarly, D-prolinol derivative **12** showed low catalytic activity (Table S1, entry 2). Pro-Gly dimer **13** was moderately efficient in the presence of benzoic acid (Table S1, entry 4). However, no noticeable ee was observed. The α–dipeptide X_L-Gly-OH **14** also showed moderate catalytic activity and a modest ee (Table S1, entry 5). These results show the suitability of γ–depeptides **3** and **4** for the Michael-Henry-hemiketalization reaction discussed in this study.

Table S1. Michael reaction between cyclohexanone **6a** and *trans*-β-nitrostyrene **7a** catalyzed by proline derivatives **11-14**.[a]

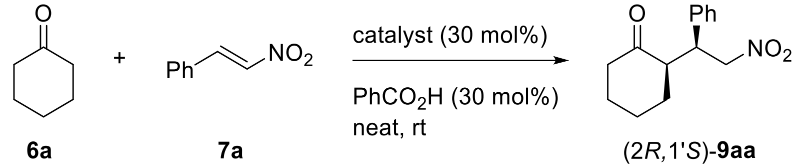

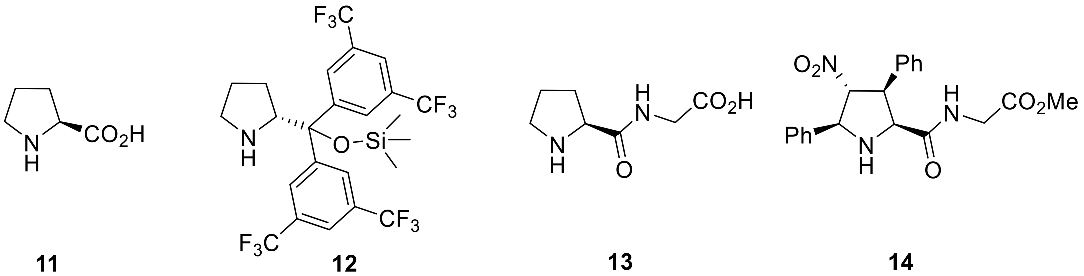

11 **12** **13** **14**

Entry	Catalyst	Time (d)	Conv[b] (%)	*syn*:*anti*[c]	Yield[d] (%)	ee[e] (%)
1	**11**	1	57	nd[f]	nd	nd
2	**12**	3	<10%	nd	nd	nd
3[f]	**13**	7	22	nd	nd	nd
4	**13**	7	72	87:13	45	0
5	**14**	7	74	78:22	38	66

[a]Reactions were conducted under neat conditions using cyclohexanone **6a** (0.8 mmol), trans-β-nitrostyrene **7a** (0.1 mmol) in the presence of the corresponding catalyst (0.03 mmol) and benzoic acid (0.03 mmol). [b]Conversions to **8aa** were measured by ^{1}H NMR of crude reaction mixtures. [c]*Syn:anti* ratio was measured by ^{1}H NMR of crude reaction mixtures. [d]Yields refer to isolated pure

Michael adducts. [e]Enantiomeric excesses measured by HPLC correspond to the major *syn*-diastereomer (2*R*,1'*S*)-**8aa**. [f]nd: not determined because of very low values and/or conversions [g]The reaction was performed in the absence of $PhCO_2H$. [g]Unreacted **7a** was present in the reaction mixture.

4 General Procedure for the One-pot Michael-Henry-Acetalization Reaction

A reaction mixture of X_L-X_L^{Me}-OMe-**4** (121.0 mg, 0.2 mmol, 0.2 eq.), acid derivative (0.2 mmol, 0.2 eq.), ketone **6a-c** (1 mmol, 1.0 eq.) and nitroolefins **7a-i** (1.1 mmol, 1.1 eq.) in 550 µL of DCM was stirred at room temperature until total consumption of the nitroalkene. This step could be monitored by NMR analysis of reaction mixtures, according to our previously described characterization of Michael adducts obtained via monomeric and dimeric species (see refs. 17 and 18 of the main text). Then, the corresponding aldehyde **8a-f** (2.0 mmol, 2 eq.) and triethylamine (30 µL, 0.2 mmol, 0.2 eq.) were successively added and the resulting reaction mixture was allowed to stir at the indicated temperature until total consumption of the intermediate γ-nitroketone. Afterwards, the crude mixture was evaporated under reduced pressure and purified by flash column chromatography on silica gel (check each compound for conditions). For the racemic compounds, the reactions were carried out using pyrrolidine (80 µL, 1.0 mmol, 1 eq.).

4.1 Screening of different chiral organocatalysts based on primary amines

The catalytic activities of quinidine derivative **15** and bifunctional amine-thiourea **16** were investigated. In both cases, the Michael step required 10 days to achieve less than 30% conversion. Then, ethyl glyoxylate (2 eq.) and triethylamine (0.2 eq) were added and the resulting reaction mixture was allowed to stir at room temperature for 1 day. The final product **10aaa** could not be distinguished in the reaction crude and the enantiomeric excess could not be determined in any case (Scheme S1).

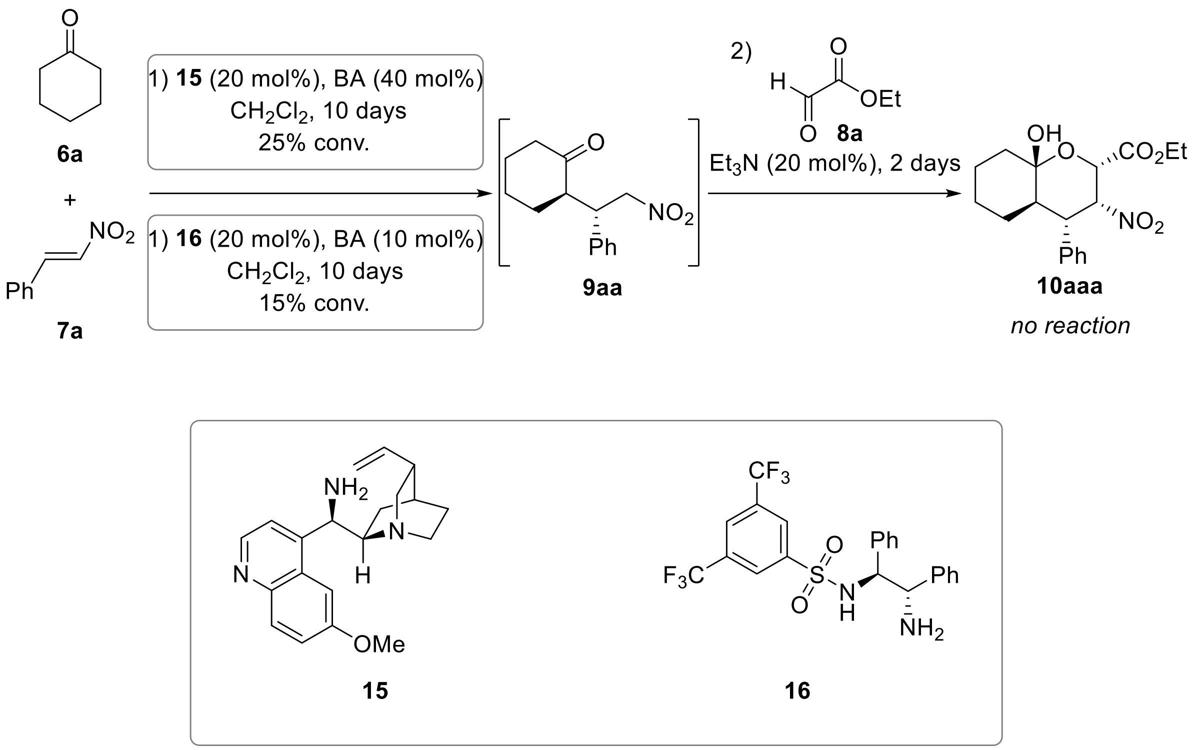

Scheme S1. Unsuccessful screening of organocatalyst **15** and **16**.

4.2 Procedure for the synthesis of 10aaa at the 5 mmol scale

A reaction mixture of X_L-X_L^{Me}-OMe-**4** (302.5 mg, 1 mmol, 0.1 eq.), salicilyc acid (XX mg, 0.2 mmol, 0.2 eq.), ketone **6a** (5 mmol, 1.0 eq.) and nitroolefins **7a** (5.0 mmol, 1.0 eq.) in 2.75 mL of DCM was stirred 3 days at room temperature until total consumption of the nitroalkene. This step could be monitored by NMR analysis of reaction mixtures, according to our previously described characterization of Michael adducts obtained via monomeric and dimeric species (see refs. 17 and 18 of the main text). Then triethylamine (140 µL, 1.0 mmol, 0.2 eq.) and the freshly distilled ethyl glyoxylate **8a** (10.0 mmol, 2.0 eq.) were successively added and the resulting reaction mixture was allowed to stir at room temperature 1 day. Afterwards, the crude mixture was evaporated under reduced pressure and purified by flash column chromatography on silica gel (1:2 EtOAc:Hexane mixture)) to provided **10aaa** (838 mg, 48%).

4.3 Isomerization Reaction for the Synthesis of 10aad’

A reaction mixture of **10aad** (343 mg, 1 mmol, 1 eq.) and DBU (150µL, 1 mmol, 1 eq.) in acetonitrile was stirred at room temperature for 16h. Then, the crude mixture was

evaporated under reduced pressure and purified by flash column chromatography on silica gel (1:2 EtOAc:Hexane mixture) to provided **10aad'** (234 mg, 68%).

4.4 Studies with Other Cyclic Ketones

After optimizing the reaction conditions, the scope of this process was investigated employing other cyclic ketones. Unfortunately, the reaction presented some restrictions regarding the nature of the nucleophiles. For instance, when tetrahydro-*4H*-pyran-4-one **6d** and cyclohexane-1,3-dione **6e** were selected as starting materials, no formation of the intermediate Michael adducts was observed in the presence of salicylic acid (Scheme S2). Changing the acidic additive into TFA did not still show any formation of the Michael adduct. In contrast, when cyclopentanone **6f** was employed as precursor of the corresponding nucleophilic enamine species, complex mixtures of diastereomeric adducts **10faa** and Michael intermediate *syn*-**9fa** were observed (Scheme S2). ^{1}H-NMR spectra of the crude reaction mixtures obtained in different experiments showed that previously characterized[14]. *syn* Michael adduct **9fa** and different diastereomers of cyclized product **10faa** were formed in a ca. 40:60 ratio. Analyses of the intermediate Michael adducts showed a transient 85:15 ratio of the *syn* and *anti* diastereomers of **9fa**, from which the different isomers of **10faa**, together with an unreacted amount of *syn*-**9fa** were finally observed in low yield as an inseparable mixture. Therefore, we concluded that cyclopentanone **6f** is not a suitable substrate for this reaction.

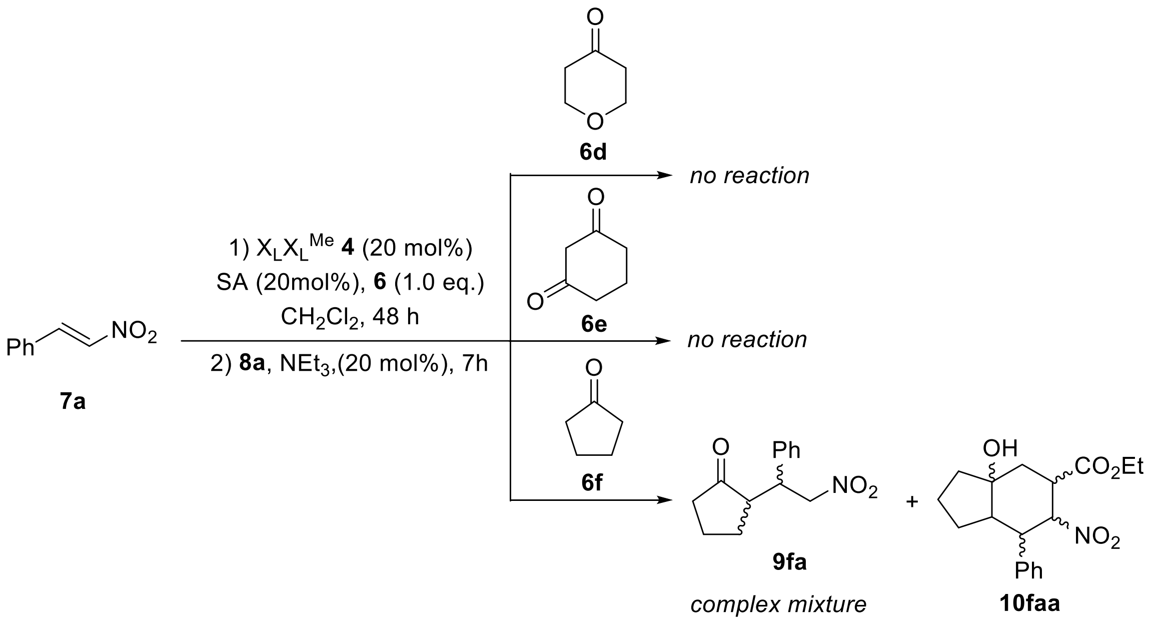

Scheme S2. Unsuccessful Michael-Henry-acetalization reactions using ketones **6d-f.**

Our attempts turned successful when cycloheptanone **6b** and 1,4-cyclohexanedione monoethylene acetal **6c** were chosen as starting materials. However, small changes were necessary for the proper synthesis of the corresponding derivatives.

Derivative **10baa** demanded equimolar amounts of Et_3N for the total consumption of the γ-nitroketone intermediate. Longer reaction times related to catalytic equivalents resulted in a severe decrease in yield. It should be mentioned that the final product was achieved in moderate yield and excellent enantioselectivites, but in a 92:8 mixture of non-separable diastereomers. Such proportion could be explained by the cyclization of both the *syn*- and *anti*- Michael adducts.

In the case of the synthesis of **10caa**, the Michael step required 7 days to reach full conversion. The following Henry-acetalization step, on the contrary, was completed in 1 hour. Hence, catalyst O_2N-X_L-X_L^{Me}-OMe-**4** provided the desired **10caa** product as a single diastereomer in 62% yield and 89% of enantiomeric excess.

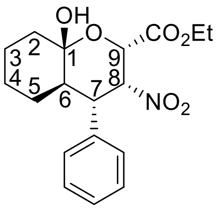

Ethyl (2S,3R,4S,4aR,8aS)-8a-hydroxy-3-nitro-4-phenyloctahydro-2H-chromene-2-carboxylate (**10aaa**). The title product was obtained from cyclohexanone **6a**, *trans*-β-nitrostyrene **7a** and ethyl glyoxylate **8a** employing salicylic acid as additive. Purified on 1:2 EtOAc:Hexane mixture. Yield 72% (251 mg, 0.72 mmol), white solid. $\mathbf{m_p}$ = 169-171 °C. $[\alpha]_D^{25}$ = +52.63 (*c* 0.95, chloroform). **FTIR** (neat, cm^{-1}) 3507, 1756, 1545, 1313. **^{1}H NMR** (400 MHz, $CDCl_3$) δ 7.32 (d, *J* = 7.4 Hz, 3H, ArH), 7.13 (d, *J* = 7.3 Hz, 2H, ArH), 5.14 (d, *J* = 3.0 Hz, 1H, C^9H), 5.12 (d, *J* = 4.4 Hz, 1H, C^8H), 4.33 – 4.13 (m, 2H, CH_2CH_3), 3.52 (dd, *J* = 12.5, 4.8 Hz, 1H, C^7H), 2.61 (td, *J* = 12.2, 3.3 Hz, 1H, C^6H), 2.09 – 1.97 (m, 2H, CH_2, OH), 1.95 (s, 1H, CH_2), 1.78 (d, *J* = 13.7 Hz, 1H, CH_2), 1.74 – 1.60 (m, 2H, CH_2), 1.39 (d, *J* = 14.4 Hz, 1H, CH_2), 1.23 (t, *J* = 7.2 Hz, 3H, CH_2CH_3), 1.18 – 0.98 (m, 1H, CH_2), 0.92 – 0.79 (m, 1H, CH_2). **^{13}C{H} NMR** (101 MHz, $CDCl_3$) δ 167.7 (C=O), 136.2(ArC), 129.1 (ArC), 128.3 (ArC), 128.2 (ArC), 98.1 (C^1), 86.9 (C^8), 69.3(C^9), 62.3 (CH_2CH_3), 44.1 (C^7), 38.9 (C^6), 38.5 (CH_2), 26.2 (CH_2), 25.6 (CH_2), 23.1 (CH_2), 14.1 (CH_2CH_3). **HRMS** (ESI) m/z: $[M+H]^+$ Calcd for $C_{18}H_{24}NO_6$ 350.1603; Found 350.1605. **HPLC** (Chiralpak IA, Hexane:^{i}PrOH = 95:5, flow rate 1 mL/min, λ = 210 nm), t_R (major) = 21.56 min, t_R (minor) = 31.23 min; *ee* = 99%.

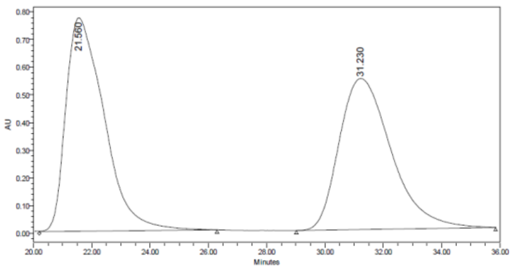
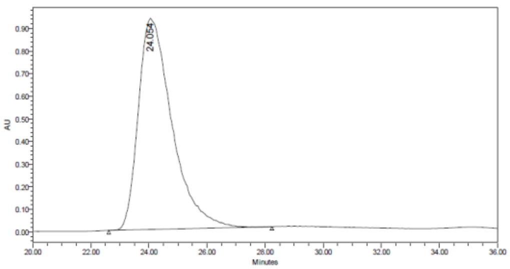

	RT	Height	Area	% Area
**1**	21.560	772486	70549380	50.95
**2**	31.230	546319	67923484	49.05

	RT	Height	Area	% Area
**1**	24.054	932234	74986663	100.0
**2**				

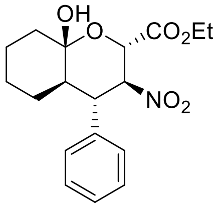

Ethyl (2S,3S,4S,4aR,8aS)-8a-hydroxy-3-nitro-4-phenyloctahydro-2H-chromene-2-carboxylate (**10aaa'**). The title product was obtained from cyclohexanone **6a**, *trans*-β-nitrostyrene **7a** and ethyl glyoxylate **8a** employing salicylic acid as additive and 1 equivalent of DBU. Purified on 1:2 EtOAc:Hexane mixture. Yield 65% (226 mg, 0.65 mmol), white solid. **m_p** = 137-139 °C. $[\alpha]_D^{25}$ = -18.28 (*c* 0.90, chloroform). **FTIR** (neat, cm^{-1}) 3471, 1739, 1550, 1373. **^{1}H NMR** (400 MHz, $CDCl_3$) δ 7.39 – 7.23 (m, 3H, ArH), 7.18 (d, *J* = 21.5 Hz, 2H, ArH), 5.20 (d, *J* = 10.3 Hz, 1H, C^9H), 4.88 (dd, *J* = 11.3, 10.3 Hz, 1H, C^8H), 4.34 – 4.09 (m, 2H, CH_2CH_3), 3.54 (t, *J* = 11.6 Hz, 1H, C^7H), 2.25 (d, *J* = 1.7 Hz, 1H, OH), 1.94 – 1.68 (m, 4H, C^6H, CH_2), 1.66 (m, 1H, CH_2), 1.62 – 1.52 (m, 1H, CH_2), 1.25 (t, *J* = 7.2 Hz, 3H, CH_2CH_3)), 1.22 – 1.05 (m, 3H, CH_2). **^{13}C{H} NMR** (101 MHz, $CDCl_3$) δ 168.4 (C=O), 136.6 (ArC), 129.2 (ArC), 128.2 (ArC), 97.7 (C^1), 89.0 (C^8), 70.2 (C^9), 62.4 (CH_2CH_3), 46.7 (C^7), 46.4 (C^6), 38.3 (CH_2), 26.2 (CH_2), 25.5 (CH_2), 23.0 (CH_2), 14.0 (CH_2CH_3). **HRMS** (ESI) m/z: $[M+H]^+$ Calcd for $C_{18}H_{24}NO_6$ 350.1603; Found 350.1605. **HPLC** (Chiralpak IC, Hexane:^{i}PrOH = 95:5, flow rate 1 mL/min, λ = 210 nm), t_R (minor) = 14.87 min, t_R (major) = 24.39 min; *ee* = 98%.

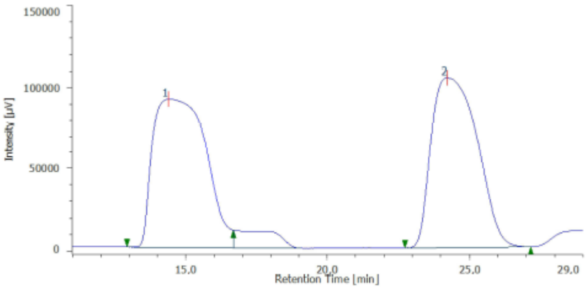
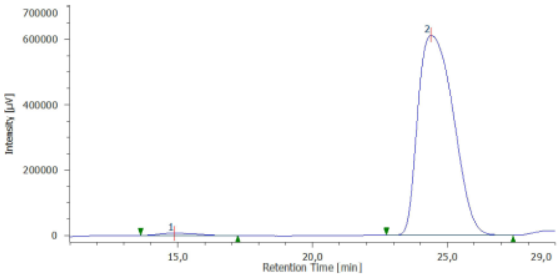

tR [min]	Area [μV·sec]	Height [μV]	Area%	Height%
14,387	11815621	90825	50,742	46,576
24,253	11470134	104178	49,258	53,424

tR [min]	Area [μV·sec]	Height [μV]	Area%	Height%
14,867	633602	6689	1,121	1,083
24,387	55907490	611194	98,879	98,917

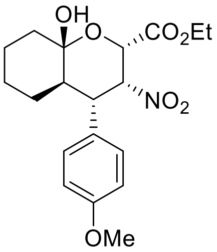

Ethyl (2S,3R,4S,4aR,8aS)-8a-hydroxy-4-(4-methoxyphenyl)-3-nitrooctahydro-2H-chromene-2-carboxylate (**10aba**). The title product was obtained from cyclohexanone **6a**, *trans*-4-methoxy-β-nitrostyrene **7b** and ethyl glyoxylate **8a** employing salicylic acid as additive. Purified on 1:2 EtOAc:Hexane mixture. Yield 65% (247 mg, 0.65 mmol), pale brown solid. **m$_p$** = 166-168 °C. $[\alpha]_D^{25}$ = +57.84 (*c* 0.75, chloroform). **FTIR** (neat, cm^{-1}) 3460, 2939, 1754, 1548, 1514, 1249. **^{1}H NMR** (400 MHz, $CDCl_3$) δ 7.08 – 6.98 (m, 2H, ArH), 6.85 (d, *J* = 8.6 Hz, 2H, ArH), 5.12 (d, *J* = 3.2 Hz, 1H, C^{9}H), 5.08 (dd, *J* = 4.9, 3.2 Hz, 1H, C^{8}H), 4.32 – 4.22 (m, 1H, C*H*$_2$CH$_3$), 4.22 – 4.13 (m, 1H, C*H*$_2$CH$_3$), 3.78 (s, 3H, OCH_3), 3.46 (dd, *J* = 12.5, 4.8 Hz, 1H, C^{7}H), 2.55 (td, *J* = 12.4, 3.2 Hz, 1H, C^{6}H), 2.22 (bs, 1H, OH), 2.04 – 1.87 (m, 2H, CH_2), 1.83 – 1.73 (m, 1H, CH_2), 1.71 – 1.54 (m, 2H, CH_2), 1.42 – 1.35 (m, 1H, CH_2), 1.31 (dt, *J* = 12.9, 4.0 Hz, 1H, CH_2), 1.23 (t, *J* = 7.1 Hz, 3H, CH$_2$C*H*$_3$), 1.08 (qd, *J* = 12.6, 3.4 Hz, 1H, CH_2). **^{13}C{H} NMR** (126 MHz, $CDCl_3$) δ 167.7, 159.3, 129.3, 128.2, 114.5, 98.1, 87.2, 69.3, 62.3, 55.3, 43.28, 39.2, 38.5, 26.2, 25.7, 23.1, 14.1. **HRMS** (ESI) m/z $[M+Na]^+$ Calcd for $C_{19}H_{25}NO_7Na$ 402.1521; Found 402.1514. **HPLC** (Chiralpak IA, Hexane:^{i}PrOH = 95:5, flow rate 1 mL/min, λ = 210 nm), t$_R$ (major) = 29.22 min, t$_R$ (minor) = 45.97 min, *ee* = 96%.

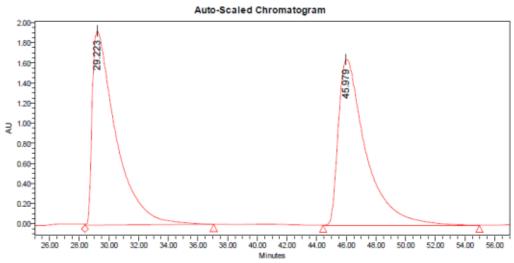

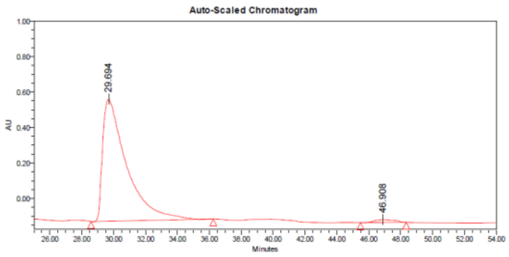

	RT	Height	Area	% Area
**1**	29.223	1932825	214068574	50.19
**2**	45.979	1652558	212420464	49.81

	RT	Height	Area	% Area
**1**	29.694	688406	71643586	98.05
**2**	46.908	16266	1427149	1.95

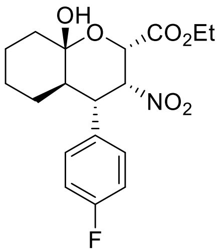

Ethyl (2S,3R,4S,4aR,8aS)-4-(4-fluorophenyl)-8a-hydroxy-3-nitrooctahydro-2H-chromene-2-carboxylate (**10aca**). The title product was obtained from cyclohexanone **6a**, *trans*-4-fluoro-β-nitrostyrene **7c** and ethyl glyoxylate **8a** employing salicylic acid as additive. Purified on 1:2 EtOAc:Hexane mixture. Yield 62% (228 mg, 0.62 mmol), white solid. **m_p** = 179-181 °C. $[\alpha]_D^{25}$ = +57.03 (*c* 0.42, chloroform). **FTIR** (neat, cm^{-1}) 3483, 2937, 1747, 1548, 1225. **^{1}H NMR** (400 MHz, $CDCl_3$) δ 7.13 – 7.06 (m, 2H, ArH), 7.02 (t, *J* = 8.6 Hz, 2H, ArH), 5.13 (d, *J* = 3.1 Hz, 1H, C^9H), 5.08 (dd, *J* = 4.8, 3.2 Hz, 1H, C^8H), 4.27 (dq, *J* = 10.7, 7.1 Hz, 1H, CH_2CH_3), 4.19 (dq, *J* = 10.8, 7.1 Hz, 1H, CH_2CH_3), 3.51 (dd, *J* = 12.5, 4.8 Hz, 1H, C^7H), 2.55 (tdd, *J* = 12.5, 3.3, 1.5 Hz, 1H, C^6H), 2.12 (s, 1H, OH), 2.05 – 1.90 (m, 2H, CH_2), 1.77 (ddt, *J* = 11.1, 4.5, 2.2 Hz, 1H, CH_2), 1.72 – 1.65 (m, 1H, CH_2), 1.59 (d, *J* = 6.7 Hz, 1H, CH_2), 1.41 – 1.28 (m, 2H, CH_2), 1.23 (t, *J* = 7.1 Hz, 3H, CH_2CH_3), 1.09 (qd, *J* = 14.1, 13.3, 4.2 Hz, 1H, CH_2). **^{13}C{H} NMR** (126 MHz, $CDCl_3$) δ 167.6, 162.5 (d, $^1J_{C-F}$ = 246.9 Hz), 131.9 (d, $^4J_{C-F}$ = 3.3 Hz), 129.9, 116.1 (d, $^2J_{C-F}$ = 21.5 Hz), 98.1, 86.9, 69.3, 62.4, 43.4, 39.2, 38.5, 26.2, 25.6, 23.1, 14.1. **^{19}F NMR** (376 MHz, $CDCl_3$) δ -113.91. **HRMS** (ESI) m/z $[M+K]^+$ Calcd for $C_{18}H_{22}FNO_6K$ 406.1062; Found 406.1058. **HPLC** (Chiralpak IA, Hexane:^{i}PrOH = 95:5, flow rate 1 mL/min, λ = 210 nm), t_R (major) = 24.74 min, t_R (minor) = 51.81 min, *ee* = 99%.

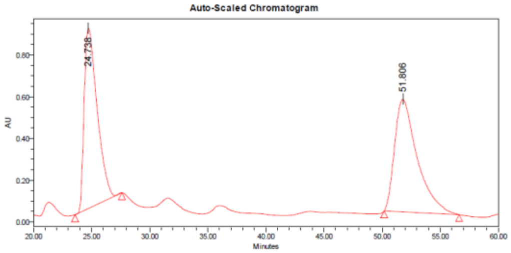

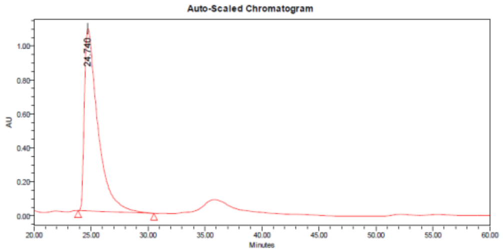

	RT	Height	Area	% Area
1	24.738	863261	66962000	47.46
2	51.806	540955	73245314	52.24

	RT	Height	Area	% Area
1	24.740	1075388	38657722	100.0
2				

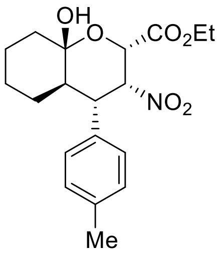

Ethyl (2S,3R,4S,4aR,8aS)-8a-hydroxy-3-nitro-4-(p-tolyl)octahydro-2H-chromene-2-carboxylate (**10ada**). The title product was obtained from cyclohexanone **6a**, *trans*-4-methyl-β-nitrostyrene **7d** and ethyl glyoxylate **8a** employing salicylic acid as additive. Purified on 1:2 EtOAc:Hexane mixture. Yield 47% (171 mg, 0.47 mmol), white solid. $\mathbf{m_p}$ = 174-177 °C. $[\alpha]_D^{25}$ = +57.69 (*c* 0.5, chloroform). **FTIR** (neat, cm^{-1}) 3475, 1750, 1549, 1372. ^{1}**H NMR** (500 MHz, $CDCl_3$) δ 7.13 (d, *J* = 7.7 Hz, 2H, ArH), 7.01 (d, *J* = 7.6 Hz, 2H, ArH), 5.13 (d, *J* = 3.0 Hz, 1H, C^9H), 5.09 (s, 1H, C^8H), 4.27 (dd, *J* = 10.8, 7.0 Hz, 1H, CH_2CH_3), 4.17 (dd, *J* = 11.0, 6.9 Hz, 1H, CH_2CH_3), 3.48 (dd, *J* = 12.5, 4.9 Hz, 1H, C^7H), 2.57 (td, *J* = 12.5, 3.4 Hz, 1H, C^6H), 2.32 (s, 3H, CH_3), 2.16 (s, 1H, OH), 2.00 (td, *J* = 13.7, 4.3 Hz, 1H, CH_2), 1.93 (d, *J* = 14.1 Hz, 1H, CH_2), 1.77 (d, *J* = 13.7 Hz, 1H, CH_2), 1.71 – 1.53 (m, 2H, CH_2), 1.44 – 1.26 (m, 2H, CH_2), 1.23 (t, *J* = 7.1 Hz, 3H, CH_2CH_3), 1.09 (qd, *J* = 12.8, 3.4 Hz, 1H, CH_2). ^{13}**C{H} NMR** (126 MHz, $CDCl_3$) δ 167.7, 137.8, 133.1, 129.8, 128.1, 98.2, 87.1, 69.4, 62.3, 43.7, 39.0, 38.6, 26.2, 25.6, 23.1, 21.2, 14.1. **HRMS** (ESI) m/z $[M+H]^+$ Calcd for $C_{19}H_{26}NO_6$ 364.1760; Found 364.1948. **HPLC** (Chiralpak IA, Hexane:^{i}PrOH = 95:5, flow rate 1 mL/min, λ = 210 nm), t_R (major) = 18.53 min, t_R (minor) = 28.20 min, *ee* = 95%.

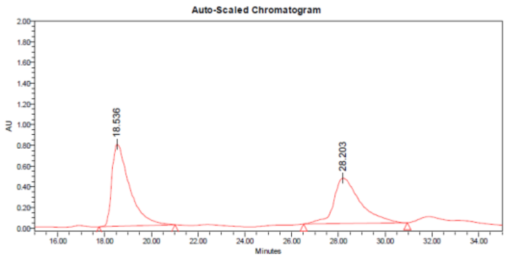

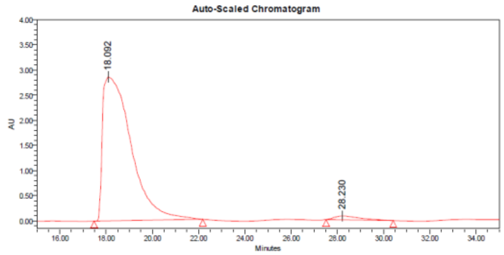

	RT	Height	Area	% Area
1	18.536	791336	41797044	54.69
2	28.203	439680	34632155	45.31

	RT	Height	Area	% Area
1	18.092	2861341	24594501	97.63
2	28.230	77664	5977755	2.37

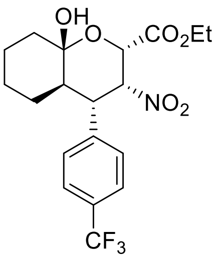

Ethyl (2S,3R,4S,4aR,8aS)-8a-hydroxy-3-nitro-4-(4-(trifluoromethyl)phenyl)octahydro-2H-chromene-2-carboxylate (**10aea**). The title product was obtained from cyclohexanone **6a**, *trans*-4-trifluoromethyl-β-nitrostyrene **7e** and ethyl glyoxylate **8a** employing salicylic acid as additive. Purified on 1:2 EtOAc:Hexane mixture. Yield 60% (250 mg, 0,60 mmol), white solid. $\mathbf{m_p}$ = 167-169 °C. $[\alpha]_D^{25}$ = +27.00

(c 0.80, chloroform). **FTIR** (neat, cm^{-1}) 3474, 2938, 1751, 1551, 1325. **^{1}H NMR** (400 MHz, $CDCl_3$) δ 7.60 (d, J = 8.1 Hz, 2H, ArH), 7.31 – 7.21 (m, 2H, ArH), 5.15 (d, J = 3.1 Hz, 1H, C^9H), 5.11 (dd, J = 4.8, 3.2 Hz, 1H, C^8H), 4.28 (dq, J = 10.8, 7.2 Hz, 1H, CH_2CH_3), 4.19 (dq, J = 10.8, 7.1 Hz, 1H, CH_2CH_3), 3.60 (dd, J = 12.5, 4.8 Hz, 1H, C^7H), 2.61 (td, J = 12.4, 3.1 Hz, 1H, C^6H), 2.18 (s, 1H, OH), 2.06 – 1.90 (m, 2H, CH_2), 1.83 – 1.73 (m, 1H, CH_2), 1.66 (ddt, J = 31.0, 13.1, 4.2 Hz, 2H, CH_2), 1.32 (dt, J = 14.2, 3.0 Hz, 2H, CH_2), 1.24 (t, J = 7.1 Hz, 3H, CH_2CH_3), 1.11 (qd, J = 13.3, 2.8 Hz, 1H, CH_2). **^{13}C{H} NMR** (126 MHz, $CDCl_3$) δ 167.5, 140.4, 130.5 (d, $^2J_{C-F}$ = 32.7 Hz), 128.8, 126.1 (q, $^3J_{C-F}$ = 3.7 Hz), 124.04 (d, $^1J_{C-F}$ = 272.2 Hz), 97.98, 86.50, 69.27, 62.46, 43.92, 38.99, 38.44, 26.17, 25.54, 23.04, 14.06. **^{19}F NMR** (376 MHz, $CDCl_3$) δ -62.69. **HRMS** (ESI) m/z $[M+Na]^+$ Calcd for $C_{19}H_{22}F_3NO_6Na$ 440.1297; Found 440.1287. **HPLC** (Chiralpak IA, Hexane:^{i}PrOH = 95:5, flow rate 1 mL/min, λ = 210 nm), t_R (major) = 9.91 min, t_R (minor) = 22.49 min, *ee* = 98%.

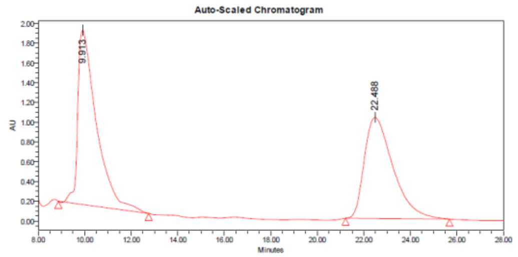

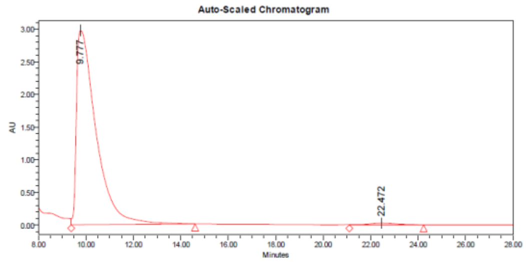

	RT	Height	Area	% Area
**1**	9.913	1763203	98242087	52.88
**2**	22.488	1023519	87536948	47.12

	RT	Height	Area	% Area
**1**	9.777	2971511	170166051	98.89
**2**	22.472	23819	1910951	1.11

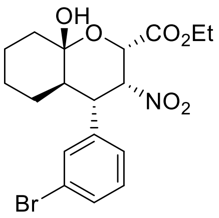

Ethyl (2S,3R,4S,4aR,8aS)-4-(3-bromophenyl)8a-hydroxy-3-nitrooctahydro-2H-chromene-2-carboxylate (**10afa**). The title product was obtained from cyclohexanone **6a**, *trans*-3-bromo-β-nitrostyrene **7f** and ethyl glyoxylate **8a** employing salicylic acid as additive. Purified on 1:2 EtOAc:Hexane mixture. Yield 54% (231 mg, 0.54 mmol), yellow oil. $[\alpha]_D^{25}$ = +41.57 (c 0.75, chloroform). **FTIR** (neat, cm^{-1}) 3469, 2938, 1750, 1549, 1339. **^{1}H NMR** (400 MHz, $CDCl_3$) δ 7.43 (dt, J = 8.2, 1.2 Hz, 1H, ArH), 7.31 (d, J = 1.9 Hz, 1H, ArH), 7.20 (t, J = 7.9 Hz, 1H, ArH), 7.04 (d, J = 7.8 Hz, 1H, ArH), 5.12 (d, J = 3.1 Hz, 1H, C^9H), 5.09 (dd, J = 4.7, 3.2 Hz, 1H, C^8H), 4.28 (dq, J = 11.0, 7.1 Hz, 1H, CH_2CH_3), 4.19 (dq, J = 10.8, 7.1 Hz, 1H, CH_2CH_3), 3.49 (dd, J = 12.4, 4.7 Hz, 1H, C^7H), 2.55 (td, J = 12.4, 3.1 Hz, 1H, C^6H), 2.05 – 1.86 (m, 2H, CH_2), 1.78 – 1.67 (m, 4H, CH_2, OH), 1.41 – 1.27 (m, 2H, CH_2),

1.24 (t, J = 7.1 Hz, 3H, CH_2CH_3), 1.12 (td, J = 12.5, 3.4 Hz, 1H, CH_2). **^{13}C{H} NMR** (126 MHz, $CDCl_3$) δ 167.5, 138.6, 131.4, 130.7, 123.1, 98.0, 86.6, 69.3, 62.4, 43.8, 38.9, 38.5, 26.2, 25.6, 23.1, 14.1. **HRMS** (ESI) m/z $[M+Na]^+$ Calcd for $C_{18}H_{22}BrNO_6Na$ 452.0528; Found 452.0494. **HPLC** (Chiralpak IA, Hexane:^{i}PrOH = 95:5, flow rate 1 mL/min, λ = 210 nm), t_R (major) = 8.28 min, t_R (minor) = 10.27 min, *ee* = 99%.

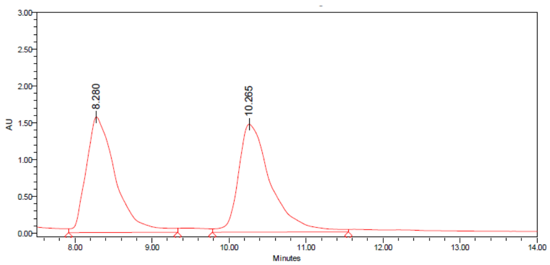

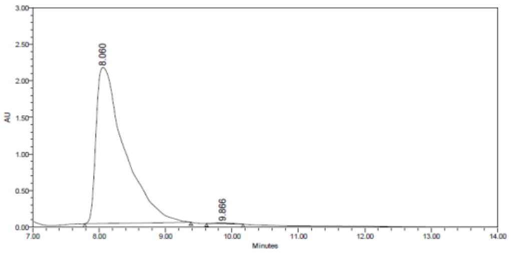

	RT	Height	Area	% Area
1	8.280	1569463	41788944	49.32
2	10.265	1464174	42949216	50.68

	RT	Height	Area	% Area
1	8.063	2140905	63722679	99.53
2	9.864	16484	302834	0.47

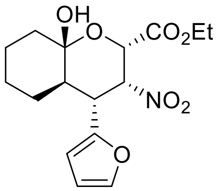

Ethyl (2S,3R,4R,4aR,8aS)-4-(furan-2-yl)-8a-hydroxy-3-nitrooctahydro-2H-chromene-2-carboxylate (**10aga**). The title product was obtained at 0 °C from cyclohexanone **6a**, *trans*-2-(2-nitrovinyl)furan **7g** and ethyl glyoxylate **8a** employing salicylic acid as additive . Purified on 1:2 EtOAc:Hexane mixture. The product was obtained as an inseparable 96:4 mixture of diastereomers. Yield 73% (248 mg, 0.73 mmol), white solid. **m_p** = 173-175 °C. $[\alpha]_D^{25}$ = +89.49 (*c* 0.85, chloroform). **FTIR** (neat, cm^{-1}) 3480, 2938, 1754, 1552, 1209. **^{1}H NMR** (500 MHz, $CDCl_3$) δ 7.34 (dd, J = 1.8, 0.8 Hz, 1H, ArH), 6.31 (dd, J = 3.2, 1.9 Hz, 1H, ArH), 6.17 (d, J = 3.2 Hz, 1H, ArH), 5.19 (dd, J = 4.8, 3.1 Hz, 1H, C^8H), 5.05 (d, J = 3.1 Hz, 1H, C^9H), 4.31 – 4.23 (m, 1H, CH_2CH_3), 4.19 (dq, J = 10.7, 7.1 Hz, 1H, CH_2CH_3), 3.66 (dd, J = 12.5, 4.8 Hz, 1H, C^7H), 2.48 (td, J = 12.4, 3.4 Hz, 1H, C^6H), 2.24 (s, 1H, OH), 1.92 (t, J = 4.1 Hz, 2H, CH_2), 1.79 – 1.67 (m, 2H, CH_2), 1.66 – 1.53 (m, 1H, CH_2), 1.44 – 1.37 (m, 1H, CH_2), 1.35 – 1.27 (m, 1H, CH_2), 1.24 (t, J = 7.1 Hz, 3H, CH_2CH_3), 1.22 – 1.11 (m, 1H, CH_2). **^{13}C{H} NMR** (126 MHz, $CDCl_3$) δ 167.6, 150.3, 142.6, 110.5, 108.5, 97.8, 84.8, 68.9, 62.3, 39.2, 38.3, 26.3, 25.5, 23.0, 14.1. **HRMS** (ESI) m/z $[M+H-H_2O]^+$ Calcd for $C_{16}H_{20}NO_6$ 322.1287; Found 322.1282. **HPLC** (Chiralpak IA, Hexane:^{i}PrOH = 95:5, flow rate 1 mL/min, λ = 210 nm), t_R (major) = 21.10 min, t_R (minor) = 26.41 min, *ee* = 95%.

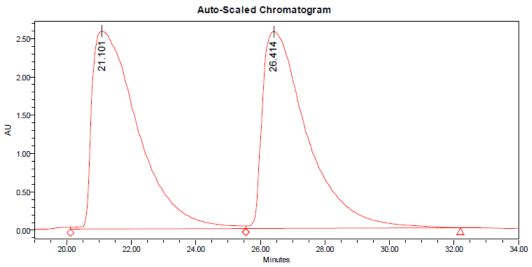

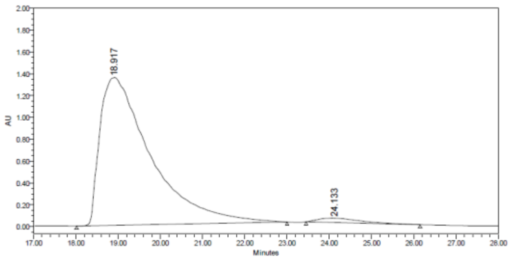

	RT	Height	Area	% Area
**1**	21.101	2583249	255048833	50.77
**2**	26.414	2566784	247343884	49.23

	RT	Height	Area	% Area
**1**	18.917	1353275	113436994	97.63
**2**	24.133	39861	2751261	2.37

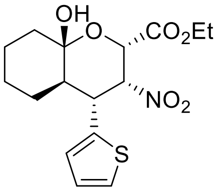

Ethyl (2S,3R,4R,4aR,8aS)-8a-hydroxy-3-nitro-4-(thiophen-2-yl)octahydro-2H-chromene-2-carboxylate (**10aha**). The title product was obtained at 0 °C from cyclohexanone **6a**, *trans*-2-(2-nitrovinyl)thiophene **7h** and ethyl glyoxylate **8a** employing salicylic acid as additive. Purified on 1:2 EtOAc:Hexane mixture. Yield 46% (164 mg, 0.46 mmol), white solid. $\mathbf{m_p}$ = 144-146 °C. $[\alpha]_D^{25}$ = +57.84 (*c* 0.75, chloroform). **FTIR** (neat, cm^{-1}) 3484, 2939, 1749, 1550, 856. **^{1}H NMR** (400 MHz, $CDCl_3$) δ 7.22 (dd, *J* = 5.1, 1.1 Hz, 1H, ArH), 6.96 (dd, *J* = 5.2, 3.5 Hz, 1H, ArH), 6.84 (d, *J* = 3.5 Hz, 1H, ArH), 5.15 (m, 2H, C^9H, C^8H), 4.32 – 4.24 (m, 1H, CH_2CH_3), 4.19 (dt, *J* = 10.8, 7.1 Hz, 1H, CH_2CH_3), 3.78 (dd, *J* = 12.3, 4.5 Hz, 1H, C^7H), 2.60 (td, *J* = 12.3, 3.4 Hz, 1H, C^6H), 2.15 (s, 1H, OH), 2.04 – 1.94 (m, 1H, CH_2), 1.90 (dt, *J* = 14.0, 3.0 Hz, 1H, CH_2), 1.81 – 1.59 (m, 3H, CH_2), 1.55 – 1.45 (m, 1H, CH_2), 1.32 (dt, *J* = 13.0, 3.9 Hz, 1H, CH_2), 1.25 (t, *J* = 7.1 Hz, 3H, CH_2CH_3), 1.15 (td, *J* = 12.8, 3.5 Hz, 1H, CH_2). **^{13}C{H} NMR** (126 MHz, $CDCl_3$) δ 167.4, 138.6, 127.5, 126.0, 125.0, 98.1, 87.0, 69.3, 62.4, 40.9, 39.61, 38.4, 26.3, 25.6, 23.1, 14.1. **HRMS** (ESI) m/z $[M+Na]^+$ Calcd for $C_{16}H_{21}NO_6SNa$ 378.0979; Found 378.0978. **HPLC** (Chiralpak IA, Hexane:^{i}PrOH = 97:3, flow rate 1 mL/min, λ = 210 nm), t_R (major) = 18.25 min, t_R (minor) = 25.59 min, *ee* = 95%.

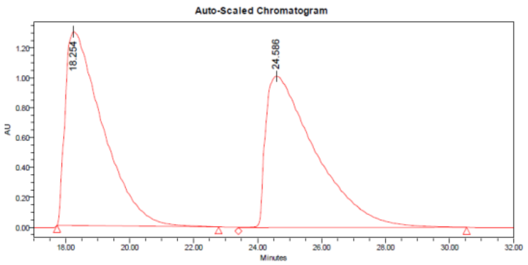

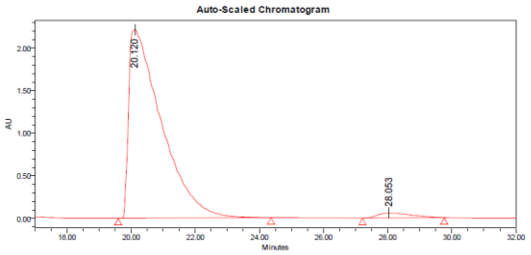

	RT	Height	Area	% Area
**1**	18.254	1296216	105677092	49.08
**2**	24.586	1011380	109649986	50.92

	RT	Height	Area	% Area
**1**	20.127	2645688	189249045	97.82
**2**	28.053	65015	4210109	2.18

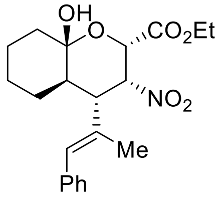

Ethyl (2S,3R,4S,4aR,8aS)-8a-hydroxy-3-nitro-4-((E)-1-phenylprop-1-en-2-yl)octahydro-2H-chromene-2-carboxylate (**10aia**). The title product was obtained at 0 °C from cyclohexanone **6a**, ((1*E*,3*E*)-2-methyl-4-nitrobuta-1,3-dien-1-yl)benzene **7i** and ethyl glyoxylate **8a** employing salicylic acid as additive. Purified on 1:2 EtOAc:Hexane mixture. Yield 55% (214 mg, 0.55 mmol), white solid. $\mathbf{m_p}$ = 157-159 °C. $[\boldsymbol{\alpha}]_{\mathbf{D}}^{\mathbf{25}}$ = +54.56 (*c* 0.60, chloroform). **FTIR** (neat, cm^{-1}) 3467, 2938, 1740, 1552, 1372. **^{1}H NMR** (400 MHz, $CDCl_3$) δ 7.36 (dd, *J* = 8.7, 6.7 Hz, 2H, ArH), 7.26 (dd, *J* = 7.9, 6.3 Hz, 3H, ArH), 6.35 (s, 1H, C*H*=C), 5.22 (dd, *J* = 4.9, 3.2 Hz, 1H, C^8H), 5.08 (d, *J* = 3.2 Hz, 1H, C^9H), 4.40 – 4.30 (dq, *J* = 10.8, 7.1 Hz, 1H, CH_2CH_3), 4.25 (dq, *J* = 10.8, 7.1 Hz, 1H, CH_2CH_3), 3.02 (dd, *J* = 12.3, 4.9 Hz, 1H, C^7H), 2.37 (td, *J* = 12.2, 2.9 Hz, 1H, C^6H), 2.18 (s, 1H, OH), 2.06 – 1.92 (m, 2H, CH_2), 1.88 (d, *J* = 1.3 Hz, 3H, CH_3C=CH), 1.85 – 1.76 (m, 3H, CH_2), 1.73 – 1.60 (m, 1H, CH_2), 1.38 (dq, *J* = 12.8, 5.3, 4.5 Hz, 1H, CH_2), 1.30 (d, *J* = 7.1 Hz, 3H, CH_2CH_3), 1.24 (dd, *J* = 13.0, 9.7 Hz, 1H, CH_2). **^{13}C{H} NMR** (126 MHz, $CDCl_3$) δ 167.8, 137.3, 132.8, 129.6, 129.1, 128.2, 126.9, 97.9, 84.8, 69.2, 62.4, 46.6, 38.6, 38.4, 26.1, 25.7, 23.1, 14.1. **HRMS** (ESI) m/z $[M+H]^+$ Calcd for $C_{21}H_{28}NO_6$ 390.1922; Found 390.1924. **HPLC** (Chiralpak IB, Hexane:^{i}PrOH = 95:5, flow rate 1 mL/min, λ = 210 nm), t_R (major) = 13.30 min, t_R (minor) = 16.24 min, *ee* = 99%.

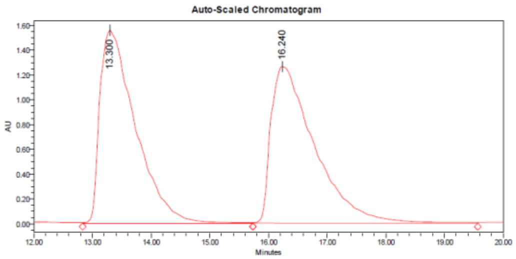

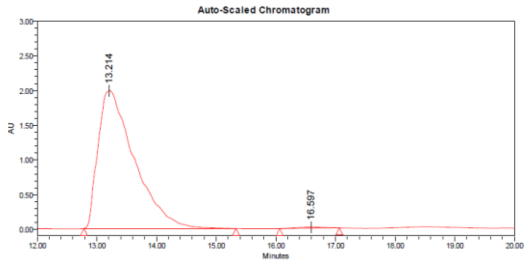

	RT	Height	Area	% Area
**1**	13.300	1555120	65385927	49.88
**2**	16.240	1264166	65691329	50.12

	RT	Height	Area	% Area
**1**	13.214	1984612	83765571	99.40
**2**	16.597	15026	503719	0.60

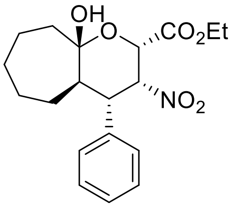

Ethyl (2S,3R,4S,4aR,9aS)-9a-hydroxy-3-nitro-4-phenyldecahydrocyclohepta[b]pyran-2-carboxylate (**10baa**). The title product was obtained from cycloheptanone **6b**, *trans*-β-nitrostyrene **7a** and ethyl glyoxylate **8a** employing TFA as additive. Purified on 1:1 Diethyl ether:hexane mixture. The product was obtained as an inseparable 92:8 mixture of diastereomers. Yield: 53% (193 mg, 0.53 mmol), colorless oil. $[\alpha]_D^{25}$ = +42.64 (*c* 0.25, chloroform). **FTIR** (neat, cm^{-1}) 3404, 2982, 1740, 1552, 1370. **^{1}H NMR** (400 MHz, $CDCl_3$) δ 7.38 (dd, *J* = 8.1, 6.5 Hz, 2H, ArH), 7.34 – 7.25 (m, 3H, ArH), 5.25 (dd, *J* = 11.1, 2.3 Hz, 1H, C^9H), 4.35 – 4.16 (m, 2H, C$H_2$$CH_3$), 4.16 – 4.08 (m, 1H, C^8H), 3.94 – 3.86 (m, 1H, C^7H), 3.24 – 3.14 (m, 1H, OH), 2.85 (ddd, *J* = 11.6, 8.1, 3.8 Hz, 1H, C^6H), 2.38 – 2.21 (m, 1H, CH_2), 1.88 – 1.73 (m, 3H, CH_2), 1.66 (d, *J* = 53.8 Hz, 3H, CH_2), 1.43 (s, 1H, CH_2), 1.28 (d, *J* = 7.1 Hz, 3H, CH_2CH_3), 1.25 (d, *J* = 4.0 Hz, 1H, CH_2), 1.22 – 1.06 (m, 1H, CH_2). **^{13}C{H} NMR** (101 MHz, $CDCl_3$) δ 170.8, 136.5, 129.2, 129.1, 128.1, 90.1, 69.8, 62.8, 55.2, 46.8, 42.8, 29.5, 28.3, 28.1, 24.8, 14.0. **HRMS** (ESI) m/z Calcd for $[M+H]^+$ $C_{19}H_{26}NO_6$ 364.1760; Found 364.1761. **HPLC** (Chiralpak IC, Hexane:^{i}PrOH = 90:10, flow rate 1 mL/min, λ = 214 nm), t_R (minor) = 17.35 min, t_R (major) = 19.42 min, *ee* = 90%.

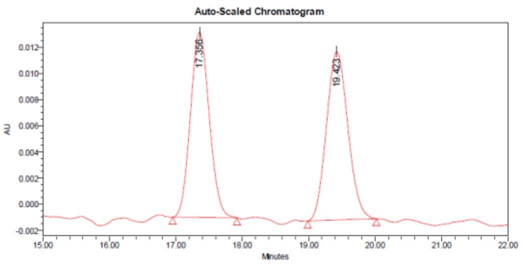

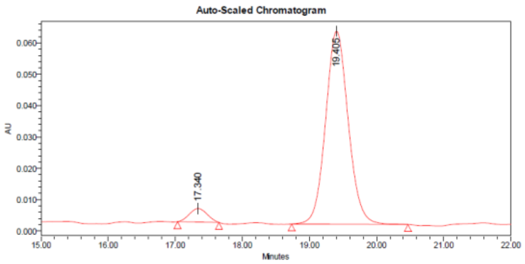

	RT	Height	Area	% Area
**1**	17.356	14190	279399	49.08
**2**	31.230	12896	289826	50.92

	RT	Height	Area	% Area
**1**	17.340	4299	78850	5.18
**2**	17.405	61608	1443594	94.82

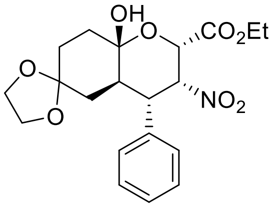

Ethyl (2S,3R,4S,4aR,8aS)-8a-hydroxy-3-nitro-4-phenylhexahyhdro-2H,5H-spiro[chromene-6,2'-[1,3]dioxolane]-2-carboxylate (**10caa**). The title product was obtained from 1,4-cyclohexanedione monoethylene acetal **6c**, *trans*-β-nitrostyrene **7a** and ethyl glyoxylate **8a** employing salicylic acid as additive. Purified on 1:2 EtOAc:Hexane mixture. Yield: 62% (253 mg, 0.62 mmol), colorless oil. $[\alpha]_D^{25}$ = +13.31 (*c* 0.7, chloroform). **FTIR** (neat, cm^{-1}) 3445, 2963, 1734, 1550, 1370. **^{1}H NMR** (400 MHz, $CDCl_3$) δ 7.39 – 7.26 (m, 3H, ArH), 7.18 – 7.12 (m, 2H, ArH), 5.13 (d, *J* = 3.1 Hz, 1H, C^9H), 5.10 (dd, *J* = 4.8, 3.2 Hz, 1H, C^8H), 4.28 (dd, *J* = 10.8, 7.1 Hz, 1H, CH_2CH_3), 4.26 – 4.07 (m, 1H, CH_2CH_3), 3.98 – 3.92 (m, 1H, CH_2O), 3.86 (ddd, *J* = 12.5, 6.9, 5.5 Hz, 2H, CH_2O), 3.82 – 3.74 (m, 1H, CH_2O), 3.53 (dd, *J* = 12.7, 4.8 Hz, 1H, C^7H), 3.00 (ddd, *J* = 12.7, 9.9, 6.5 Hz, 1H, CH_2), 2.35 (td, *J* = 15.1, 4.8 Hz, 1H, C^6H), 2.21 (s, 1H, OH), 2.01 – 1.87 (m, 2H, CH_2), 1.86 – 1.75 (m, 1H, CH_2), 1.45 (dd, *J* = 8.7, 1.8 Hz, 2H, CH_2), 1.23 (t, *J* = 7.1 Hz, 3H, CH_2CH_3). **^{13}C{H} NMR** (126 MHz, $CDCl_3$) δ 167.7, 136.0, 129.5, 128.6, 108.6, 97.7, 86.5, 69.9, 64.9, 64.7, 62.7, 43.9, 36.2, 35.9, 35.1, 32.3. **HRMS** (ESI) m/z $[M+H-H_2O]^+$ Calcd for $C_{20}H_{24}NO_7$ 390.1546; Found 390.1541. **HPLC** (Chiralpak IA, Hexane:^{i}PrOH = 95:5, flow rate 1 mL/min, λ = 210 nm), t_R (major) = 24.06 min, t_R (minor) = 39.51 min, *ee* = 88%.

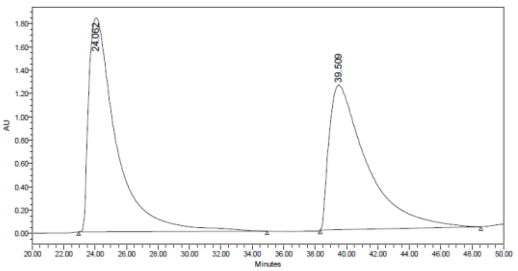

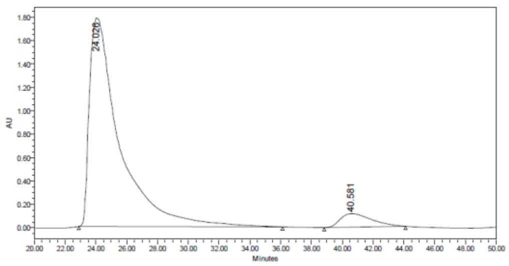

	RT	Height	Area	% Area
**1**	24.062	1834217	224008004	51.93
**2**	39.509	1242793	207365753	48.07

	RT	Height	Area	% Area
**1**	24.026	1781292	242669095	93.70
**2**	40.581	116739	16322493	6.30

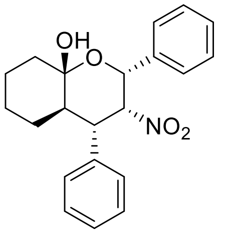

(2R,3R,4S,4aR,8aS)-3-nitro-2,4-diphenyloctahydro-8aH-chromen-8a-ol (**10aab**). The title product was obtained from cyclohexanone **6a**, *trans*-β-nitrostyrene **7a** and benzaldehyde **8b** employing salicylic acid as additive and 1 equivalent of triethylamine. Purified on 1:3 EtOAc:Hexane mixture. Global yield 65%. Isolated yield 30% (107 mg, 0.30 mmol), white solid. **m$_p$** = 195-197 °C. $[\alpha]_D^{25}$ = +26.45 (*c* 0.40, chloroform). **FTIR** (neat, cm^{-1}) 3511, 2922, 1548, 1335. **^{1}H NMR** (500 MHz, $CDCl_3$) δ 7.40 – 7.36 (m, 2H, ArH), 7.35 – 7.28 (m, 4H, ArH), 7.27 – 7.24 (m, 2H, ArH), 7.17 (dd, *J* = 7.0, 1.7 Hz, 2H, ArH), 5.66 (d, *J* = 3.1 Hz, 1H, C^{9}H), 4.95 (dd, *J* = 4.6, 3.2 Hz, 1H, C^{8}H), 3.59 (dd, *J* = 12.5, 4.5 Hz, 1H, C^{7}H), 2.97 (ddt, *J* = 14.7, 11.4, 1.6 Hz, 1H, C^{6}H), 2.07 (td, *J* = 13.7, 4.5 Hz, 1H, CH_2), 1.92 (bs, 1H, OH), 1.87 (ddt, *J* = 13.7, 4.0, 2.1 Hz, 1H, CH_2), 1.84 – 1.76 (m, 1H, CH_2), 1.76 – 1.64 (m, 1H, CH_2), 1.48 – 1.35 (m, 2H, CH_2), 1.26 (m, 1H, CH_2), 1.19 (td, *J* = 12.6, 3.1 Hz, 1H, CH_2). **^{13}C{H} NMR** (126 MHz, $CDCl_3$) δ 136.9, 136.7, 129.2, 128.7, 128.5, 127.9, 126.0, 97.9, 91.9, 71.1, 44.4, 38.9, 38.7, 26.1, 25.9, 23.2. **HRMS** (ESI) m/z $[M+H-H_2O]^+$ Calcd for $C_{21}H_{22}NO_3$ 336.1592; Found 336.1589. **HPLC** (Chiralpak IA, Hexane:^{i}PrOH = 95:5, flow rate 1 mL/min, λ = 210 nm), t_R (major) = 21.56 min, t_R (minor) = 31.23 min, *ee* = >99%.

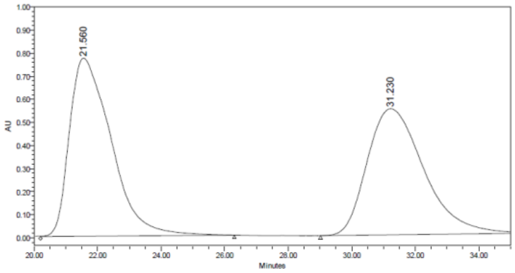

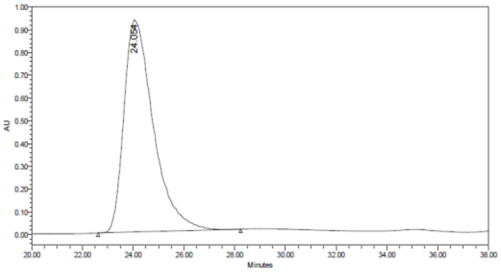

	RT	Height	Area	% Area
**1**	21.560	772486	70549380	50.95
**2**	31.230	546319	67923484	49.05

	RT	Height	Area	% Area
**1**	24.054	932234	74986663	100.0
**2**				

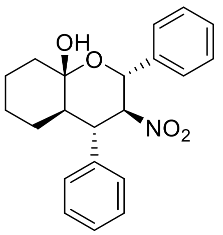

(2R,3S,4S,4aR,8aS)-3-nitro-2,4-diphenyloctahydro-8aH-chromen-8a-ol (**10aab'**). The title product was obtained from cyclohexanone **6a**, *trans*-β-nitrostyrene **7a** and benzaldehyde **8b** employing salicylic acid as additive and 1 equivalent of triethylamine. Purified on 1:3 EtOAc:Hexane mixture. Global yield 65%. Isolated yield 35% (123 mg, 0.35 mmol), white solid. $\mathbf{m_p}$ = 223-225 °C. $[\boldsymbol{\alpha}]_{\mathbf{D}}^{\mathbf{25}}$ = +31.48 (*c* 0.25, chloroform). **FTIR** (neat, cm^{-1}) 3552, 2928, 1544, 1123. ^{1}**H NMR** (400 MHz, $CDCl_3$) δ 7.42 – 7.22 (m, 10H), 5.55 (d, *J* = 10.0 Hz, 1H, C^9H), 4.77 (t, *J* = 10.6 Hz, 1H, C^8H), 3.72 (t, *J* = 11.6 Hz, 1H, C^7H), 2.17 (s, 1H, OH)2.05 (s, 1H, CH_2), 1.88 (d, *J* = 11.8 Hz, 1H, C^6H), 1.84 – 1.74 (m, 2H, CH_2), 1.69 (d, *J* = 15.3 Hz, 2H, CH_2), 1.38 – 1.22 (m, 1H, CH_2), 1.19 (d, *J* = 5.4 Hz, 2H, CH_2). ^{13}**C{H} NMR** (126 MHz, $CDCl_3$) δ 136.9, 136.7, 129.2, 128.7, 128.5, 127.9, 126.0, 97.9, 91.9, 71.1, 44.4, 38.9, 38.7, 26.1, 25.9, 23.2. **HRMS** (ESI) m/z [M+H-H_2O]+ Calcd for $C_{21}H_{22}NO_3$ 336.1592; Found 336.1591. **HPLC** (Chiralpak IA, Hexane:^{i}PrOH = 95:5, flow rate 1 mL/min, λ = 210 nm), t_R (major) = 9.17 min, t_R (minor) = 11.68 min, *ee* = 96%.

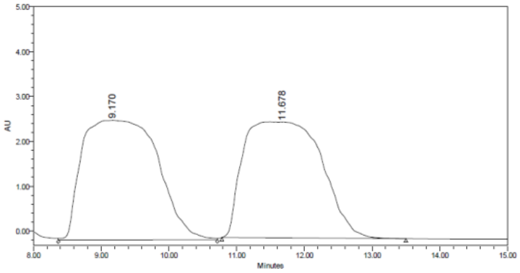

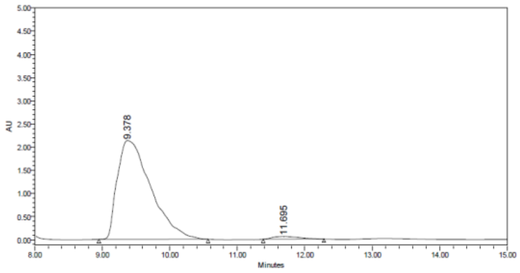

	RT	Height	Area	% Area
**1**	9.170	2662130	207821990	49.94
**2**	11.678	2582615	208361441	50.06

	RT	Height	Area	% Area
**1**	9.378	2131827	73579930	97.92
**2**	11.695	56958	1565685	2.08

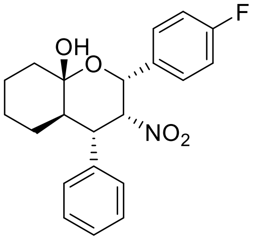

(2R,3R,4S,4aR,8aS)-2-(4-fluorophenyl)-3-nitro-4-phenyloctahydro-8aH-chromen-8a-ol (**10aac**). The title product was obtained from cyclohexanone **6a**, *trans*-β-nitrostyrene **7a** and 4-fluorobenzaldehyde **8c** employing salicylic acid as additive and 1 equivalent of triethylamine. Purified on 1:3 EtOAc:Hexane mixture. Global yield 81%. Isolated yield 54% (201 mg, 0.54 mmol), white solid. $\mathbf{m_p}$ = 198-200 °C. $[\boldsymbol{\alpha}]_{\mathbf{D}}^{\mathbf{25}}$ = +12.05 (*c* 0.60, chloroform). **FTIR** (neat, cm^{-1}) 3510, 2950, 1551, 1118. **^{1}H NMR** (400 MHz, $CDCl_3$) δ 7.39 – 7.22 (m, 5H, ArH), 7.21 – 7.11 (m, 2H, ArH), 7.01 (t, J = 8.7 Hz, 2H, ArH), 5.64 (d, J = 3.2 Hz, 1H, C^9H), 4.91 (t, J = 3.9 Hz, 1H, C^8H), 3.57 (dd, J = 12.5, 4.5 Hz, 1H, C^7H), 3.04 – 2.89 (m, 1H, C^6H), 2.05 (td, J = 13.6, 4.4 Hz, 1H, CH_2), 1.89 (d, J = 1.5 Hz, 1H, CH_2), 1.88 – 1.77 (m, 1H, CH_2), 1.77 – 1.60 (m, 2H, CH_2), 1.41 (td, J = 13.4, 12.5, 3.8 Hz, 2H, CH_2), 1.28 – 1.10 (m, 1H, CH_2). **^{13}C{H} NMR** (126 MHz, $CDCl_3$) δ 162.7 (d, $^1J_{C\text{-}F}$ = 246.8 Hz), 136.7, 132.5 (d, $^4J_{C\text{-}F}$ = 3.3 Hz), 129.2, 128.0, 127.8 (d, $^3J_{C\text{-}F}$ = 8.3 Hz), 115.7 (d, $^2J_{C\text{-}F}$ = 21.6 Hz), 97.9, 92.0, 70.5, 44.4, 38.9, 38.6, 26.1, 25.8, 23.2. **^{19}F NMR** (376 MHz, $CDCl_3$) δ -113.56. **HMRS** (ESI) m/z $[M+H-H_2O]^+$ Calcd for $C_{21}H_{21}FNO_3$ 354.1505; Found 354.1495. **HPLC** (Chiralpak IA, Hexane:^{i}PrOH = 95:5, flow rate 1 mL/min, λ = 210 nm), t_R (minor) = 18.21 min, t_R (major) = 37.22 min, *ee* = >99%.

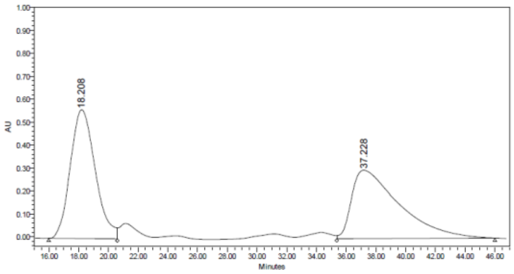

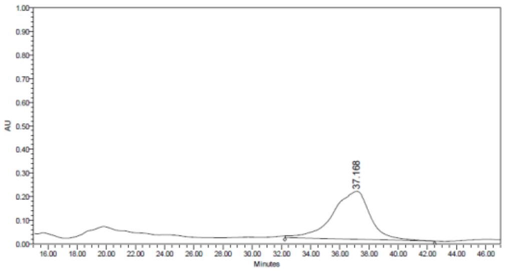

	RT	Height	Area	% Area
1	18.208	561795	65417617	49.52
2	37.228	298237	66684317	50.48

	RT	Height	Area	% Area
1				
2	37.168	203128	36380394	100.0

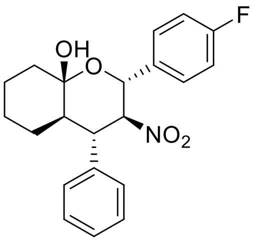

(2R,3S,4S,4aR,8aS)-2-(4-fluorophenyl)-3-nitro-4-phenyloctahydro-8aH-chromen-8a-ol (**10aac'**). The title product was obtained from cyclohexanone **6a**, *trans*-β-nitrostyrene **7a** and 4-fluorobenzaldehyde **8c** employing salicylic acid as additive and 1 equivalent of triethylamine. Purified on 1:3 EtOAc:Hexane mixture. Global yield 81%. Isolate yield 27% (100 mg, 0.27 mmol), white solid. $\mathbf{m_p}$ = 206-209 °C. $[\boldsymbol{\alpha}]_{\mathbf{D}}^{\mathbf{25}}$ = +61.88 (*c* 0.60, chloroform). **FTIR** (neat, cm^{-1}) 3484, 2936, 1547, 1115. **^{1}H NMR** (400 MHz, $CDCl_3$) δ 7.36 (dd, *J* = 8.5, 5.3 Hz, 2H, ArH), 7.31 – 7.16 (m, 5H, ArH), 7.04 (t, *J* = 8.6 Hz, 2H, ArH), 5.54 (d, *J* = 9.9 Hz, 1H, C^9H), 4.72 (t, *J* = 10.6 Hz, 1H, C^8H), 3.71 (t, *J* = 11.6 Hz, 1H, C^7H), 2.10 (s, 1H, OH), 1.88 (td, *J* = 11.8, 3.3 Hz, 1H, C^6H), 1.77 – 1.55 (m, 4H, CH_2), 1.29 – 1.11 (m, 4H, CH_2). **^{13}C{H} NMR** (126 MHz, $CDCl_3$) δ 163.2 (d, $^1J_{C\text{-}F}$ = 247.9 Hz), 136.8, 132.7 (d, $^4J_{C\text{-}F}$ = 3.3 Hz), 129.1 (d, $^3J_{C\text{-}F}$ = 8.3 Hz), 128.1, 115.9 (d, $^2J_{C\text{-}F}$ = 21.7 Hz), 97.5, 95.1, 73.2, 47.1, 46.9, 38.8, 26.2, 25.7, 23.0. **^{19}F NMR** (376 MHz, $CDCl_3$) δ -112.35. **HRMS** (ESI) m/z [M+H]+ Calcd for $C_{21}H_{23}FNO_4$ 372.1721; Found 372.1720. **HPLC** (Chiralpak IA, Hexane:^{i}PrOH = 95:5, flow rate 1 mL/min, λ = 210 nm), t_R (minor) = 28.52 min, t_R (major) = 36.43 min, *ee* = 95%.

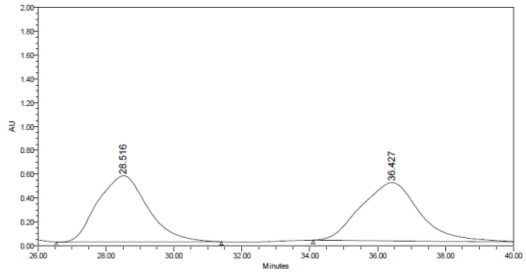

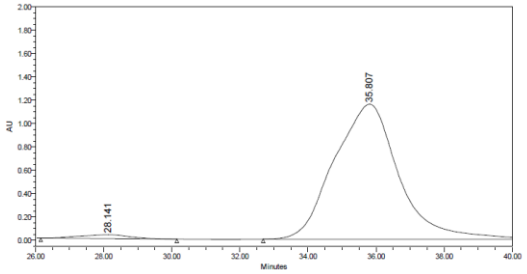

	RT	Height	Area	% Area
**1**	28.516	2034883	226075356	49.92
**2**	36.427	1062962	226837738	50.08

	RT	Height	Area	% Area
**1**	28.141	32644	3278255	2.10
**2**	35.807	1155871	152946036	97.90

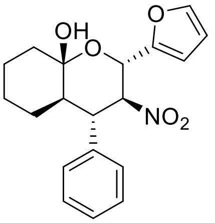

(2S,3S,4S,4aR,8aS)-2-(furan-2-yl)-3-nitro-4-phenyloctahydro-8aH-chromen-8a-ol (**10aad'**). The title product was obtained from cyclohexanone **6a**, *trans*-β-nitrostyrene **7a** and furfural **8d** employing salicylic acid as additive. When one equivalent of Et_3N was used an inseparable 50:50 mixture of **10aad:10aad'** isomers was obtained. Global yield 65% (223 mg, 0.65 mmol). **^{1}H NMR** (400 MHz, $CDCl_3$) δ 7.70 (s, 2H, ArH), 7.52 – 7.00 (m, 10H, ArH), 6.61 (s, 1H, ArH), 6.41 (d, *J* = 3.3 Hz, 1H, ArH), 6.35 (d, *J* = 3.3 Hz, 1H, ArH), 6.32 (t, *J* = 2.5 Hz, 1H, ArH), 5.71 (d, *J* = 3.0 Hz, 1H, **10aad**), 5.65 (d, *J* = 10.3 Hz, 1H, **10aad**'), 5.11 (t, *J* = 10.8 Hz, 1H, **10aad**'), 4.98 (t, *J* = 3.8 Hz, 1H, **10aad**), 3.66 (t, *J* = 11.7 Hz, 1H, **10aad**'), 3.52 (dd, *J* = 12.5, 4.4 Hz, 1H, **10aad**), 2.98 – 2.87 (m, 1H, **10aad**), 2.08 – 1.96 (m, 1H, **10aad**'), 1.93 – 1.81 (m, 2H, **10aad** and **10aad**'), 1.77 (m, 2H, **10aad** and **10aad**'), 1.66 (m, 4H, **10aad** and **10aad**'), 1.39 (m, 2H, **10aad** and **10aad**'), 1.15 (m, 8H, **10aad** and **10aad**'). The reaction performed at 1 mmol scale with 1 eq. of DBU allowed to obtain **10aad'** with complete conversion. Purified on 1:2 EtOAc:Hexane mixture. Yield 68% (230 mg, 0. 68 mmol), white solid. **m_p** = 252-254 °C. $[\alpha]_D^{25}$ = +5.66 (*c* 0.60, chloroform). **FTIR** (neat, cm^{-1}) 3539, 2935, 1546, 1124. **^{1}H NMR** (500 MHz, $CDCl_3$) δ 7.44 (d, *J* = 1.7 Hz, 1H, ArH), 7.26 (s, 5H, ArH), 6.41 (d, *J* = 3.3 Hz, 1H, ArH), 6.32 (dd, *J* = 3.3, 1.9 Hz, 1H, ArH), 5.65 (d, *J* = 10.2 Hz, 1H, C^9H), 5.11 (dd, *J* = 11.4, 10.2 Hz, 1H, C^8H), 3.66 (t, *J* = 11.6 Hz, 1H, C^7H), 2.15 (s, 1H, OH), 1.89 (tdd, *J* = 12.0, 3.7, 1.4 Hz, 1H, C^6H), 1.83 – 1.75 (m, 2H, CH_2), 1.75 – 1.67 (m, 1H, CH_2), 1.67 – 1.60 (m, 1H, CH_2), 1.32 – 1.11 (m, 4H, CH_2). **^{13}C{H} NMR** (126 MHz, $CDCl_3$) δ 149.2, 143.9, 136.9, 128.1, 110.5, 110.4, 97.6, 91.3, 67.1, 46.8, 46.7, 38.6, 26.2, 25.6, 23.0. **HPLC** (Chiralpak IA, Hexane:^{i}PrOH = 95:5, flow rate 1 mL/min,

λ = 210 nm), t_R (**10aad’** minor) = 18.00 min, t_R (**10aad** major) = 36.46 min, t_R (**10aad** minor) = 44.74 min, t_R (**10aad’** major) = 79.02 min. *ee* **10aad** = 98%. *ee* **10aad’** = 98%.

10aad

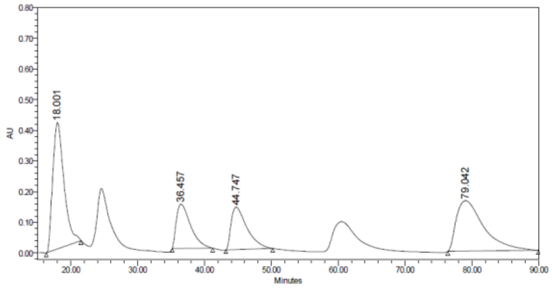

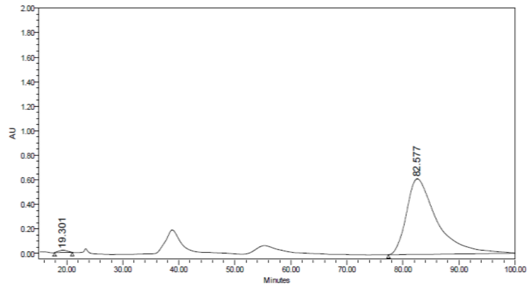

	RT	Height	Area	% Area
1	18.000	692107	82178228	35.51
2	36.456	224401	36263819	15.58
3	44.743	202476	35966472	15.45
4	79.042	267732	78351878	33.66

	RT	Height	Area	% Area
1	19.301	19103	2017127	0.86
2				
3				
4	82.577	617310	232611786	99.14

10aad’

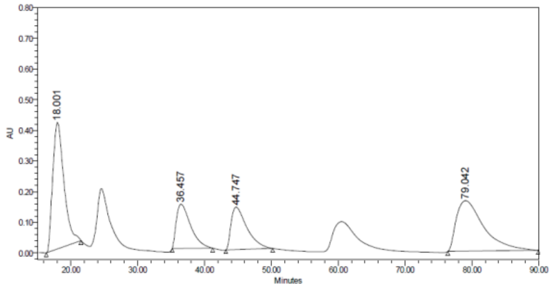

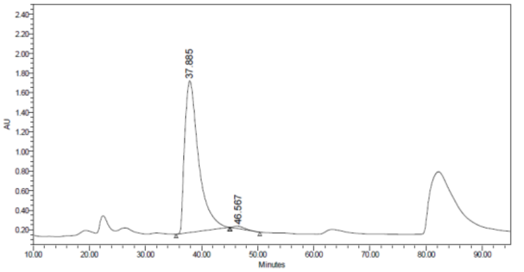

	RT	Height	Area	% Area
1	18.000	692107	82178228	35.51
2	36.456	224401	36263819	15.58
3	44.743	202476	35966472	15.45
4	79.042	267732	78351878	33.66

	RT	Height	Area	% Area
1				
2	37.885	1548286	267427094	99.19
3	46.633	19736	2173050	0.81
4				

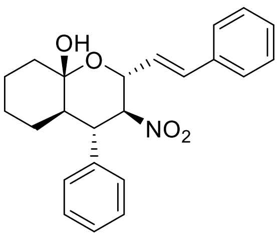

(2R,3R,4S,4aR,8aS)-3-nitro-4-phenyl-2-((E)-styryl)octahydro-8aH-chromen-8a-ol (**10aae**). The title product was obtained from cyclohexanone **6a**, *trans*-β-nitrostyrene **7a** and cinnamaldehyde **8e** employing salicylic acid as additive. Purified on 1:4 EtOAc:Hexane mixture. Global yield 58% (220 mg, 0.58 mmol), white solid. $\mathbf{m_p}$ = 198-200 °C. $[\alpha]_D^{25}$ = +58.20 (*c* 0.10, chloroform). **FTIR** (neat, cm^{-1}) 3499, 2922, 1713, 1546. **^{1}H NMR** (500 MHz, $CDCl_3$) δ 9.74 (d, *J* = 3.2 Hz, 1H, CH=C*H*Ph), 7.33 – 7.22 (m, 8H, ArH), 7.22 – 7.14 (m, 2H, ArH), 4.93 (t, *J* = 4.5 Hz, 1H, C^8H), 4.10 (dd, *J* = 12.6, 4.5 Hz, 1H, C^9H), 3.98 (dd, *J* = 12.7, 3.3 Hz, 1H, C*H*=CHPh), 3.46 (dd, *J* = 12.2, 4.5 Hz, 1H, C^7H), 2.65 (td, *J* = 12.2, 3.3 Hz, 1H, C^6H), 2.39 (s, 1H, OH), 1.89 (d, *J* = 13.6 Hz, 1H, CH_2), 1.76 (td, *J* = 13.5, 4.3 Hz, 1H, CH_2), 1.68 (d, *J* = 13.0 Hz, 2H, CH_2), 1.39 – 1.23 (m, 3H, CH_2), 1.21 – 1.10 (m, 1H, CH_2). **^{13}C{H} NMR** (126 MHz, $CDCl_3$) δ 206.9, 137.5, 136.0, 129.4, 129.2, 128.5, 128.2, 127.9, 94.9, 73.1, 54.9, 46.8, 42.9, 40.3, 37.5, 25.6, 25.1, 21.2. **HRMS** (ESI) m/z $[M+H]^+$ Calcd for $C_{23}H_{26}NO_4$ 380.1862; Found 380.1827. **HPLC** (Chiralpak IA, Hexane:^{i}PrOH = 95:5, flow rate 1 mL/min, λ = 210 nm), t_R (minor) = 36.31 min, t_R (major) = 53.53 min, *ee* = >99%.

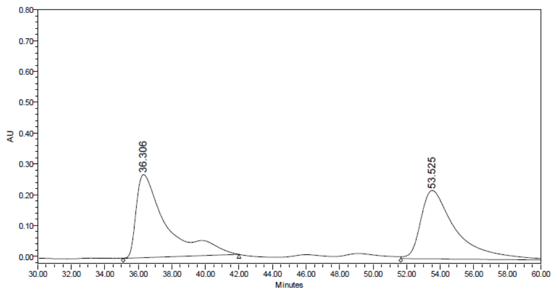

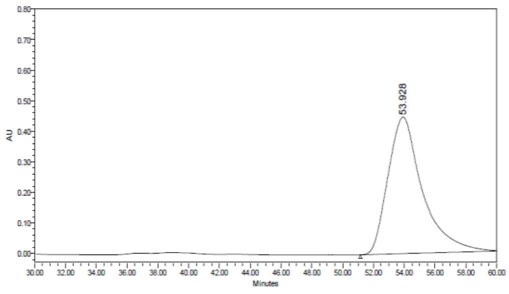

	RT	Height	Area	% Area
**1**	36.314	122720	33828331	50.96
**2**	53.522	99835	32558850	49.04

	RT	Height	Area	% Area
**1**				
**2**	53.928	447019	159851852	100.0

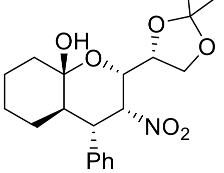

(2S,3R,4S,4aR,8aS)-2-((R)-2,2-dimethyl-1,3-dioxolan-4-yl)-3-nitro-4-phenyloctahydro-8aH-chromen-8a-ol (**10aaf**). The title product was obtained from cyclohexanone **6a**, *trans*-β-nitrostyrene **7a** and (*R*)-2,2-dimethyl-1,3-dioxolane-4-carbaldehyde **8f** employing salicylic acid as additive. Purified on 1:3 EtOAc:Hexane mixture. Yield 64% (242 mg, 0.64 mmol), colorless oil. $[\alpha]_D^{25}$ = +34.32 (*c* 0.53, chloroform). **FTIR** (neat, cm^{-1}) 3434, 2986, 1545. **^{1}H NMR** (400

MHz, $CDCl_3$) δ 7.29 (dt, *J* = 13.3, 7.0 Hz, 3H, ArH), 7.17 – 7.09 (m, 2H, ArH), 4.94 (dd, *J* = 4.7, 2.8 Hz, 1H, C^8H), 4.29 (dd, *J* = 8.9, 2.8 Hz, 1H, C^9H), 4.13 – 4.05 (m, 1H, CHO), 4.00 – 3.92 (m, 2H, CH_2O), 3.40 (dd, *J* = 12.4, 4.8 Hz, 1H, C^7H), 2.73 (d, *J* = 3.2 Hz, 1H, C^6H), 2.27 (s, 1H, OH), 1.89 – 1.78 (m, 1H, CH_2), 1.78 – 1.52 (m, 4H, CH_2), 1.44 (s, 3H, CH_3), 1.37 (dd, *J* = 12.1, 6.5 Hz, 2H, CH_2), 1.29 (s, 3H, CH_3), 1.22 – 0.97 (m, 1H, CH_2). **^{13}C{H} NMR** (126 MHz, $CDCl_3$) δ 136.8, 129.1, 127.9, 110.2, 97.5, 87.5, 74.2, 70.6, 67.5, 43.9, 39.1, 38.8, 27.1, 26.2, 25.8, 25.1, 23.2. **HRMS** (ESI) m/z $[M+H-H_2O]^+$ Calcd for $C_{20}H_{26}NO_5$ 360.1801; Found 360.1802.

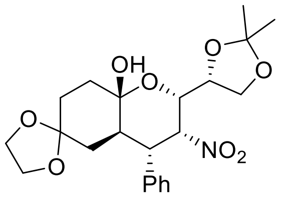

(2S,3R,4S,4aR,8aS)-2-((R)-2,2-dimethyl-1,3-dioxolan-4-yl)-3-nitro-4-phenylhexahydro-2H,8aH-spiro[chromene-6,2'-[1,3]dioxolan]-8a-ol (**10caf**). The title product was obtained from 1,4-cyclohexanedione monoethylene acetal **6c**, *trans*-β-nitrostyrene **7a** and (*R*)-2,2-dimethyl-1,3-dioxolane-4-carbaldehyde **8f** employing salicylic acid as additive. Purified on 1:2 EtOAc:Hexane mixture. Yield 52 % (227 mg, 0.52 mmol), colorless oil. $[\boldsymbol{\alpha}]_{\mathbf{D}}^{\mathbf{25}}$ = +22.62 (*c* 0.33, chloroform). **FTIR** (neat, cm^{-1}) 3393, 2960, 1546. **^{1}H NMR** (500 MHz, $CDCl_3$) δ 7.36 – 7.30 (m, 2H, ArH), 7.30 – 7.26 (m, 1H, ArH), 7.15 (dd, *J* = 7.1, 1.7 Hz, 2H, ArH), 4.93 (dd, *J* = 4.7, 2.8 Hz, 1H, C^8H), 4.29 (dd, *J* = 8.8, 2.8 Hz, 1H, C^9H), 4.09 (td, *J* = 7.3, 2.6 Hz, 1H, CHO), 3.99 – 3.97 (m, 1H, CH_2O), 3.96 – 3.94 (m, 1H, CH_2O), 3.91 – 3.83 (m, 2H, CH_2O), 3.83 – 3.77 (m, 1H, CH_2O), 3.42 (dd, *J* = 12.7, 4.6 Hz, 1H, C^7H), 3.16 – 3.08 (m, 1H, CH_2O), 2.18 (td, *J* = 13.7, 4.4 Hz, 1H, C^6H), 2.13 – 2.10 (bs, 1H, OH), 1.94 (td, *J* = 13.4, 4.5 Hz, 1H, CH_2), 1.82 – 1.71 (m, 2H, CH_2), 1.45 (d, *J* = 8.7 Hz, 2H, CH_2), 1.43 (s, 3H, CH_3), 1.29 (s, 3H, CH_3), 1.25 (s, 1H, CH_2). **^{13}C{H} NMR** (126 MHz, $CDCl_3$) δ 136.3, 129.2, 128.1, 110.3, 108.5, 96.7, 86.1, 74.2, 70.9, 67.5, 64.6, 64.4, 43.5, 36.1, 35.6, 34.7, 32.2, 27.1, 25.1. **HRMS** (ESI) m/z $[M+H-H_2O]^+$ Calcd for $C_{22}H_{28}NO_7$ 418.1859; Found 418.1857.

5 NMR spectra

Compound NO_2-X_L-Gly-OMe **14**

^{1}H NMR (500 MHz, $CDCl_3$) of 14

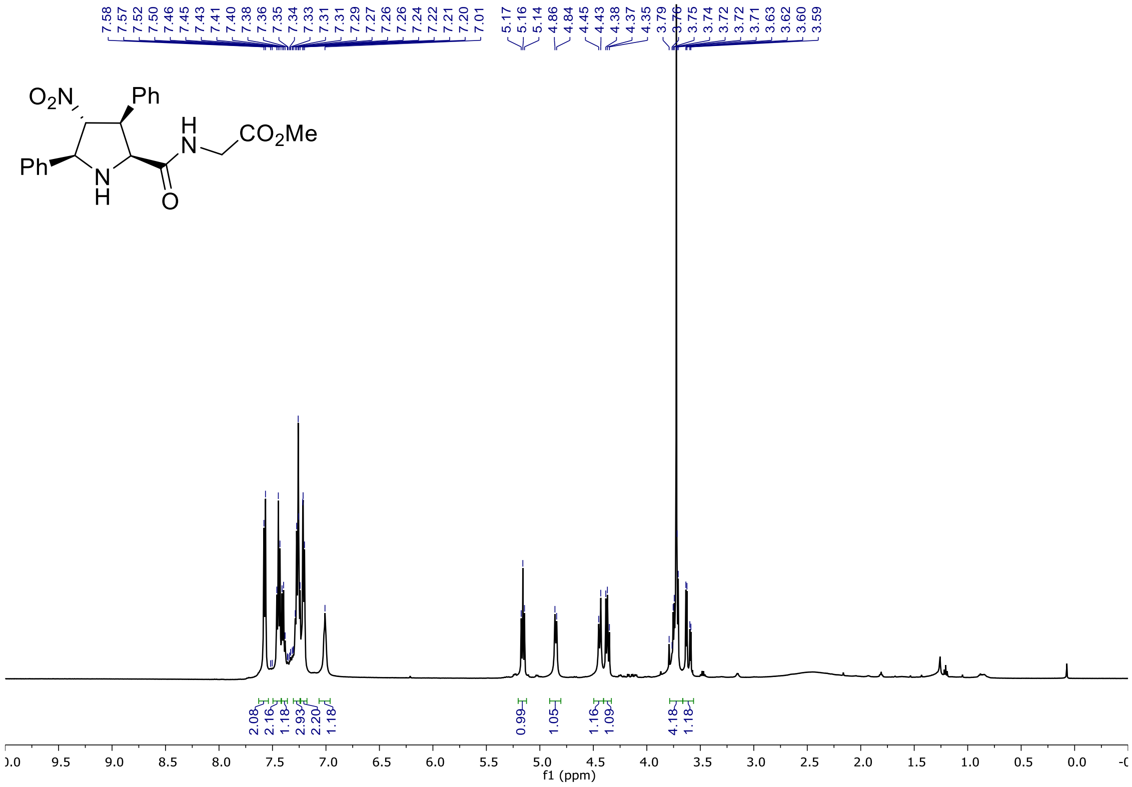

$^{13}C\{H\}$ NMR (101 MHz, $CDCl_3$) of 14

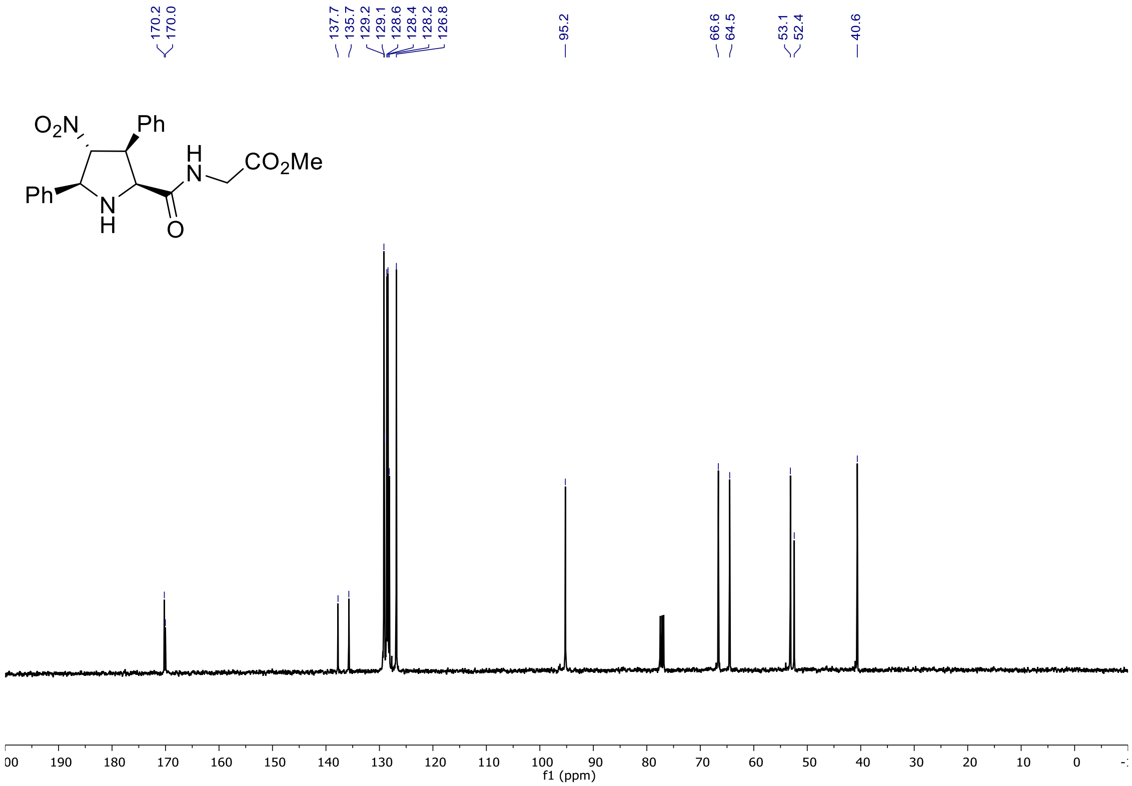

COSY ($CDCl_3$) of 14

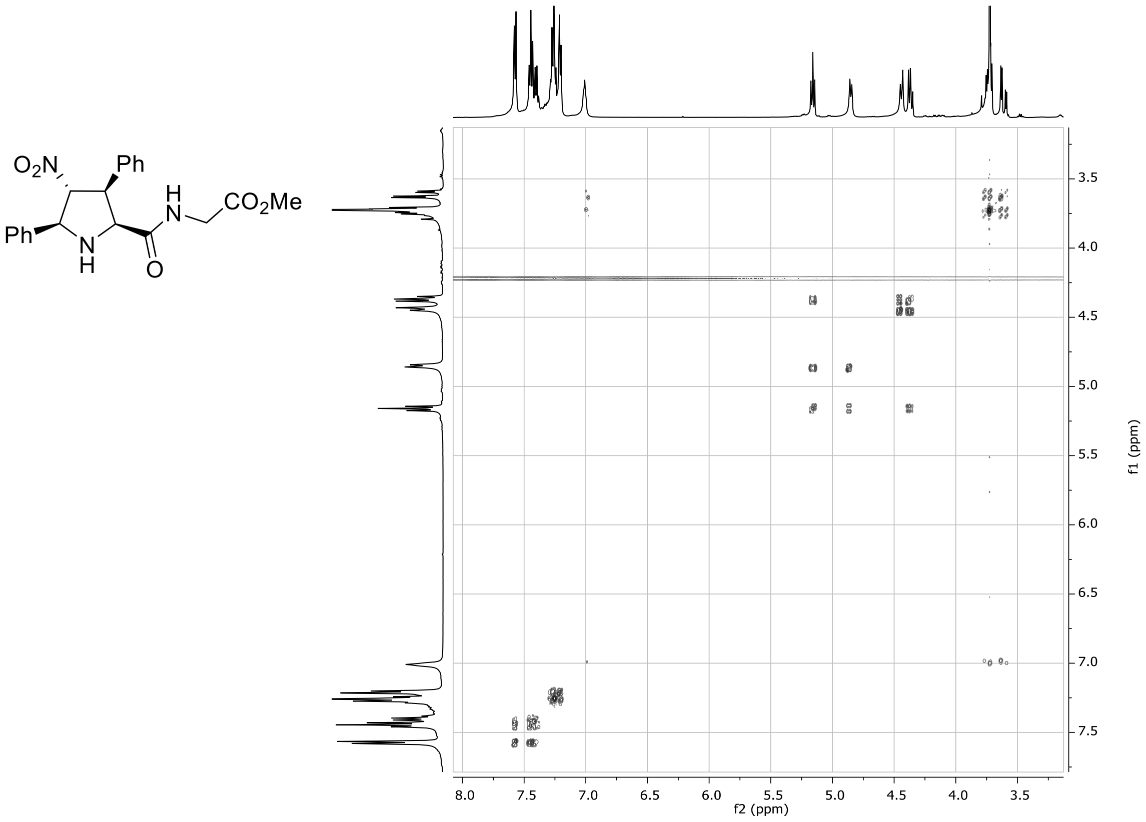

Compound **10aaa**

^{1}H NMR (400 MHz, $CDCl_3$) of 10aaa

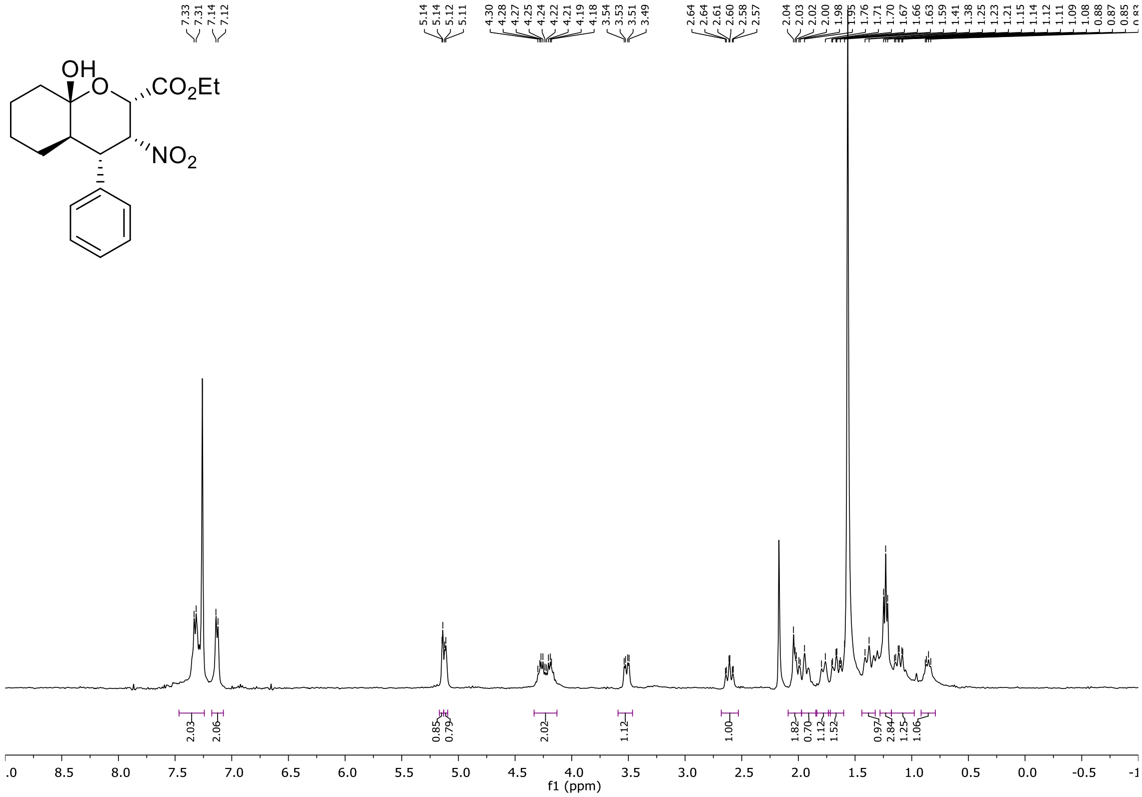

$^{13}C\{H\}$ NMR (101 MHz, $CDCl_3$) of 10aaa

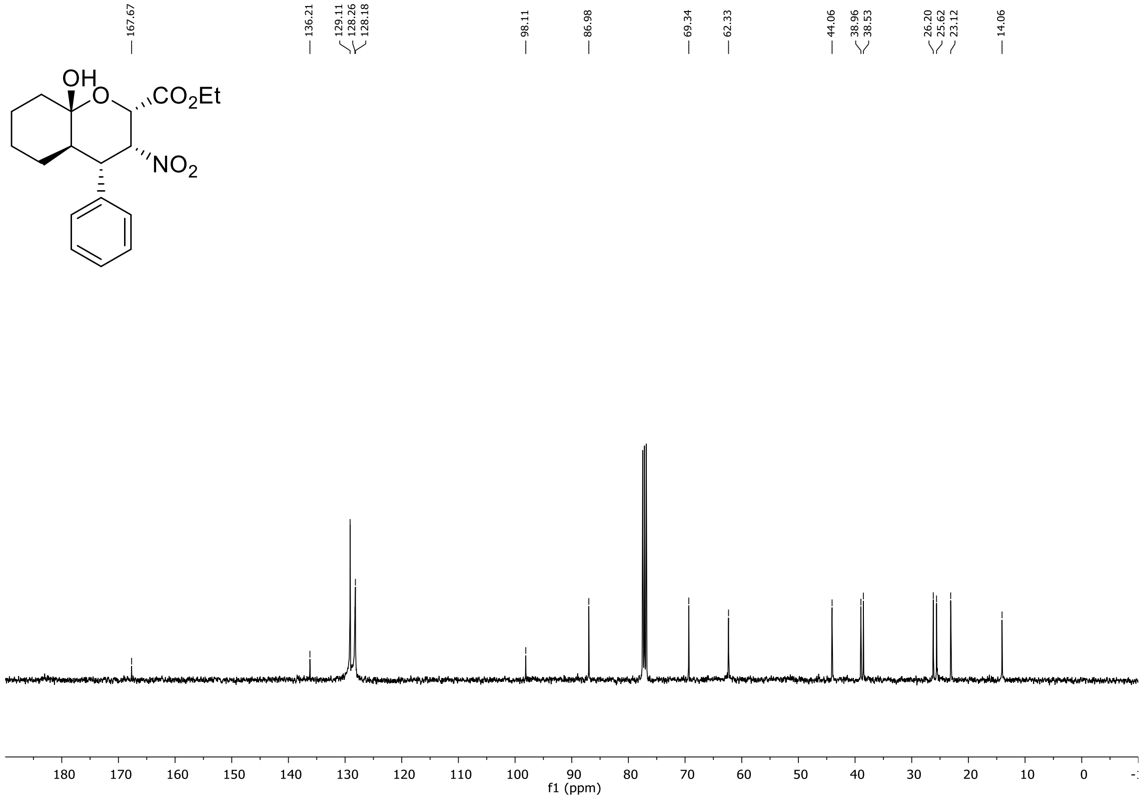

COSY ($CDCl_3$) of 10aaa

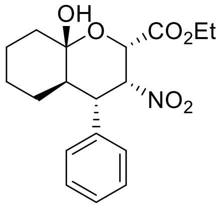

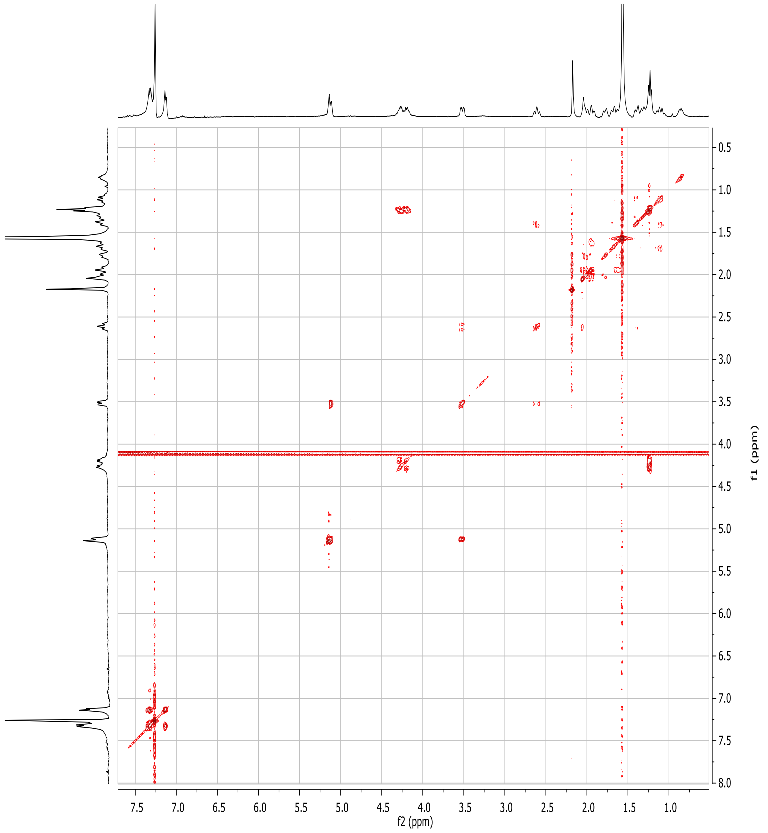

HSQC ($CDCl_3$) of 10aaa

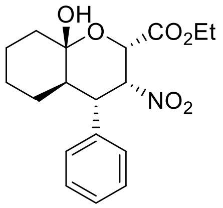

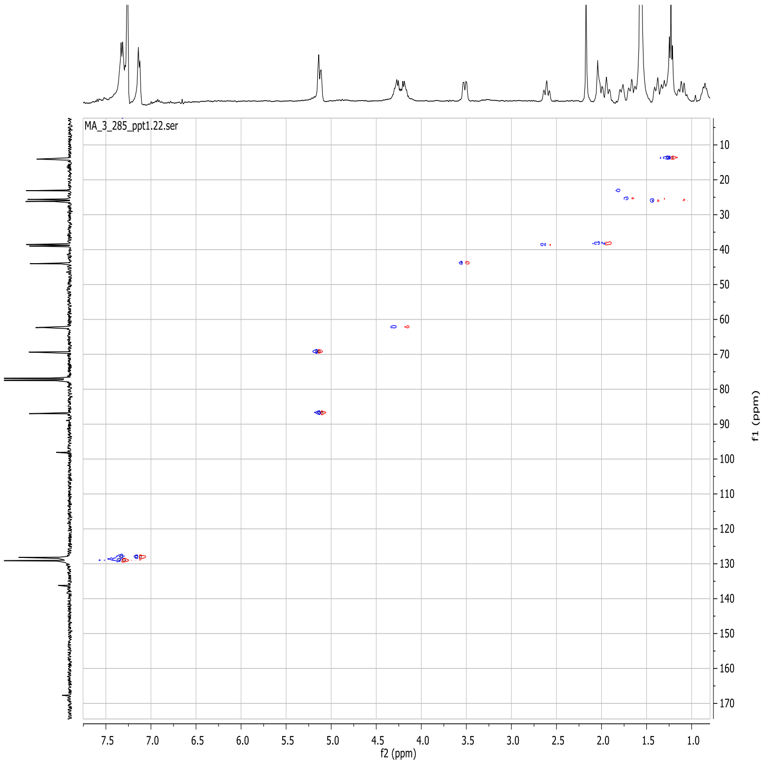

Compound **10aaa’**

^{1}H NMR (400 MHz, $CDCl_3$) of 10aaa’

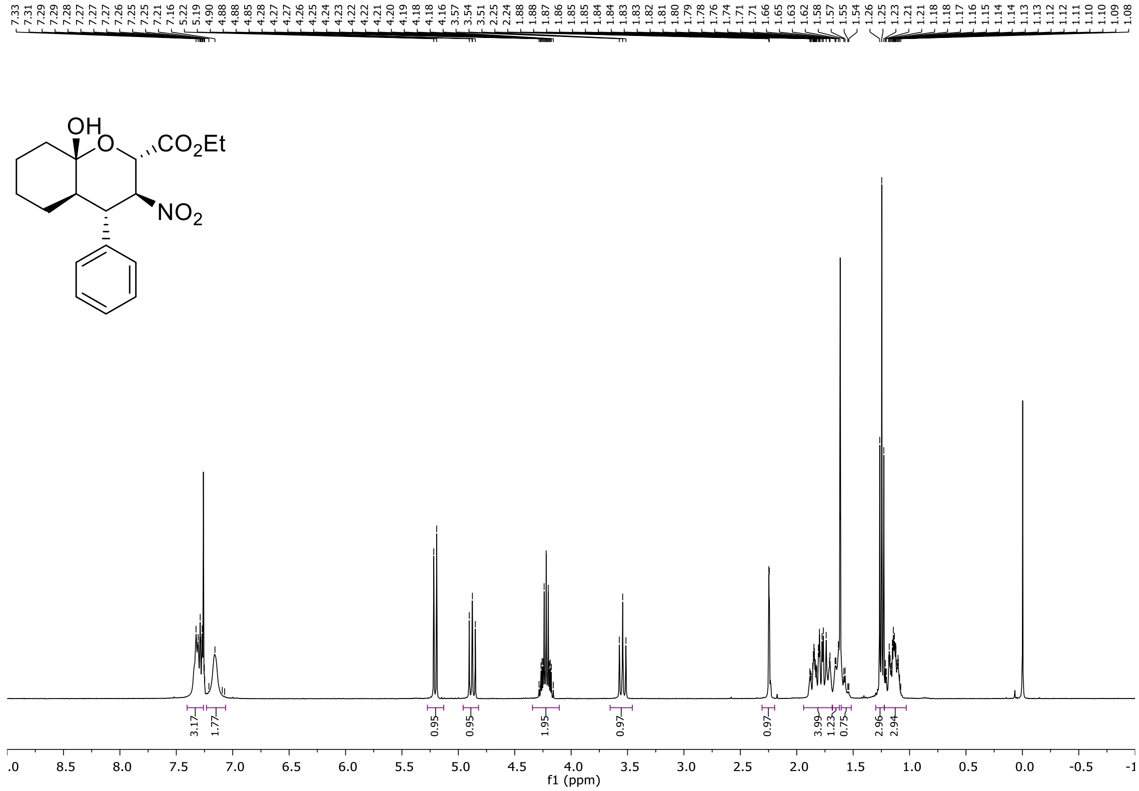

^{13}C{H} NMR (101 MHz, $CDCl_3$) of 10aaa’

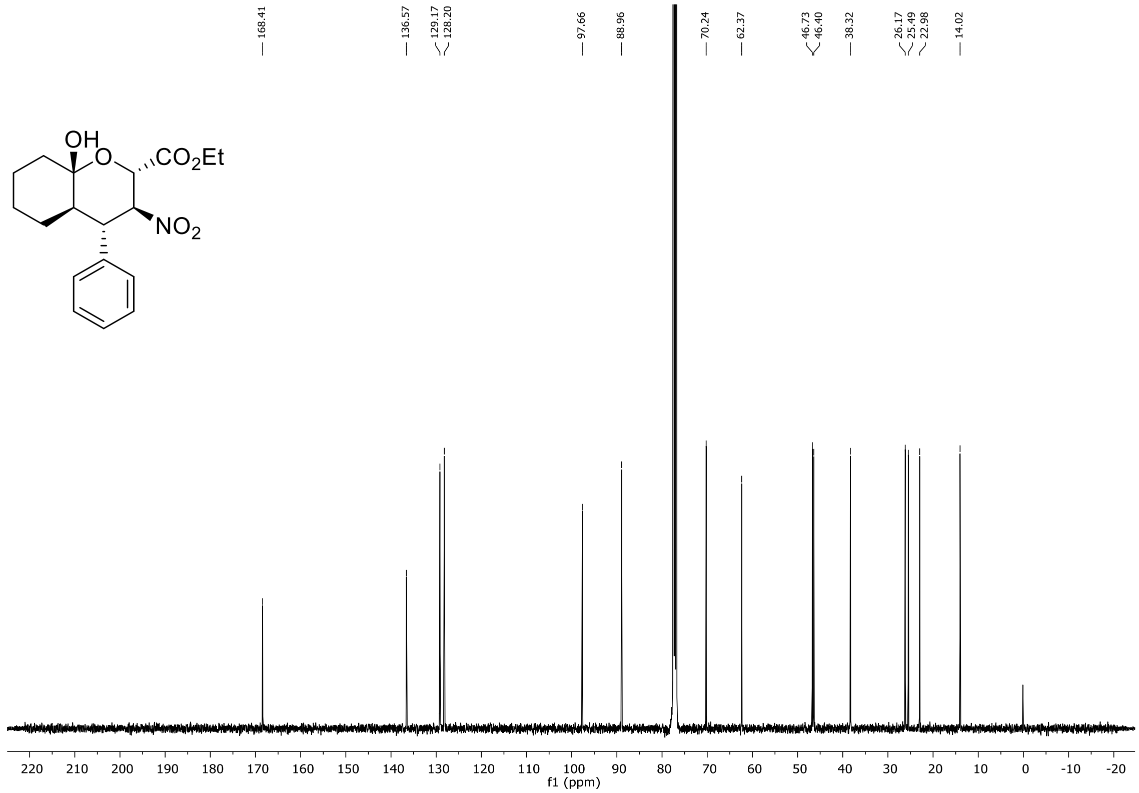

COSY ($CDCl_3$) of 10aaa’

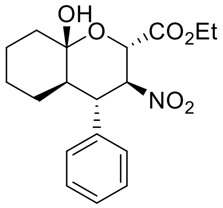

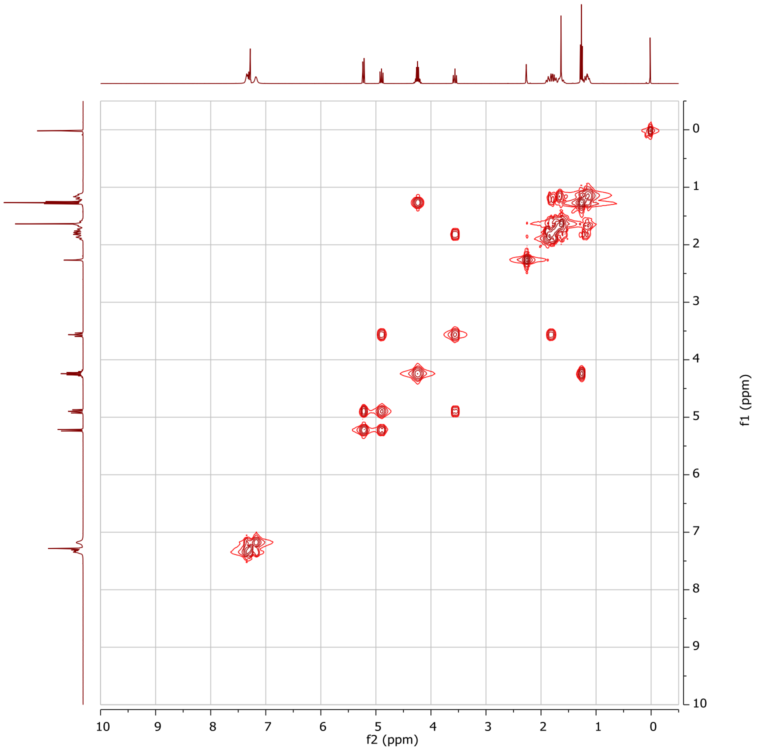

HSQC ($CDCl_3$) of 10aaa’

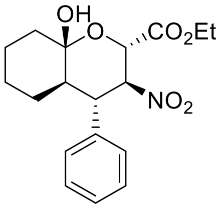

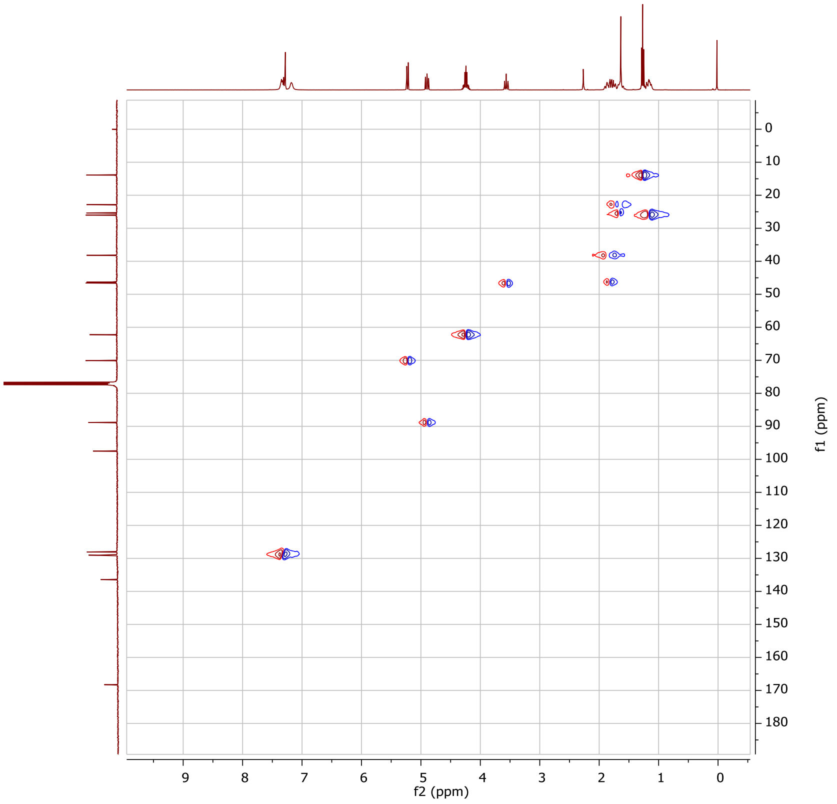

Compound **10aba**

^{1}H NMR (400 MHz, $CDCl_3$) of 10aba

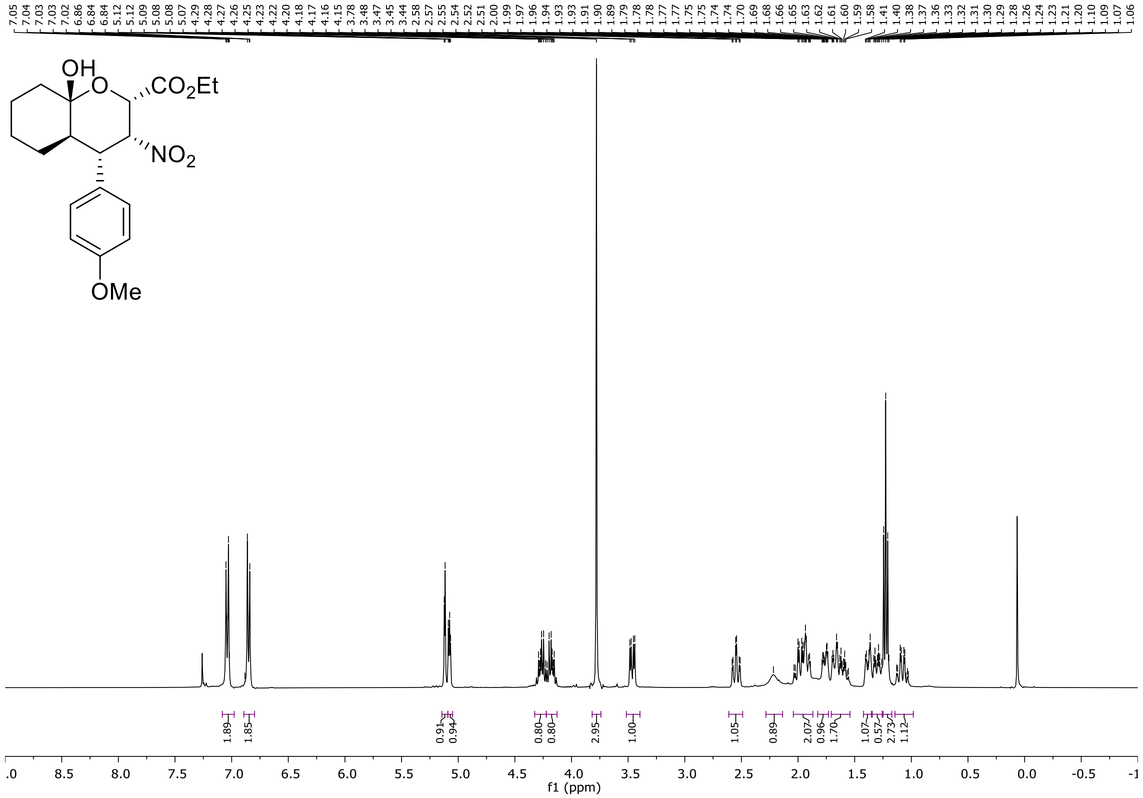

^{13}C{H} NMR (126 MHz, $CDCl_3$) of 10aba

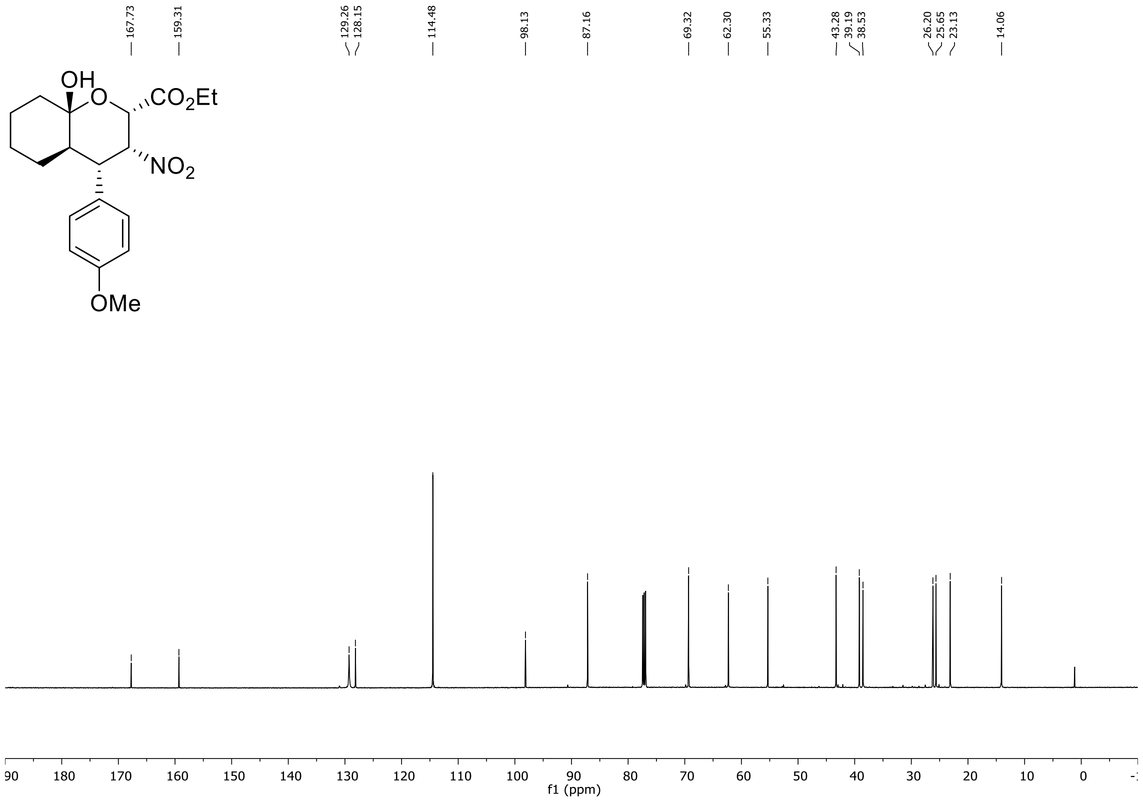

Compound **10aca**

^{1}H NMR (400 MHz, $CDCl_3$) of 10aca

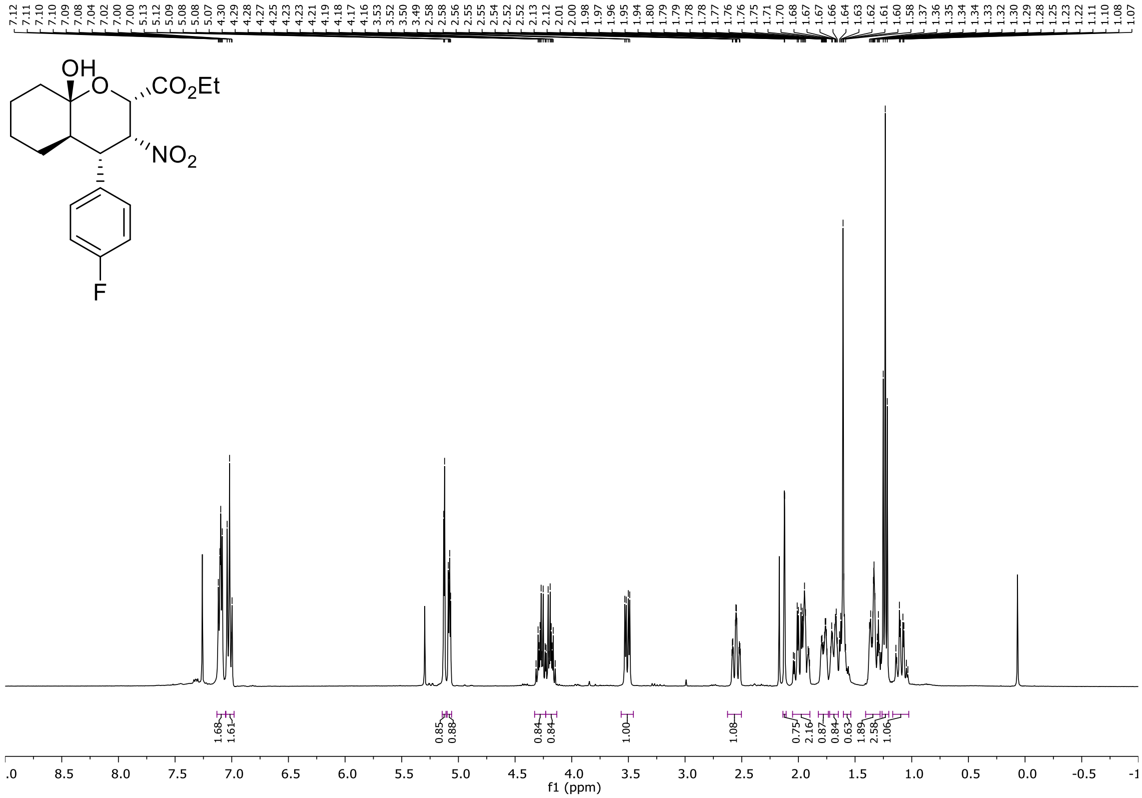

^{13}C{H} NMR (126 MHz, $CDCl_3$) of 10aca

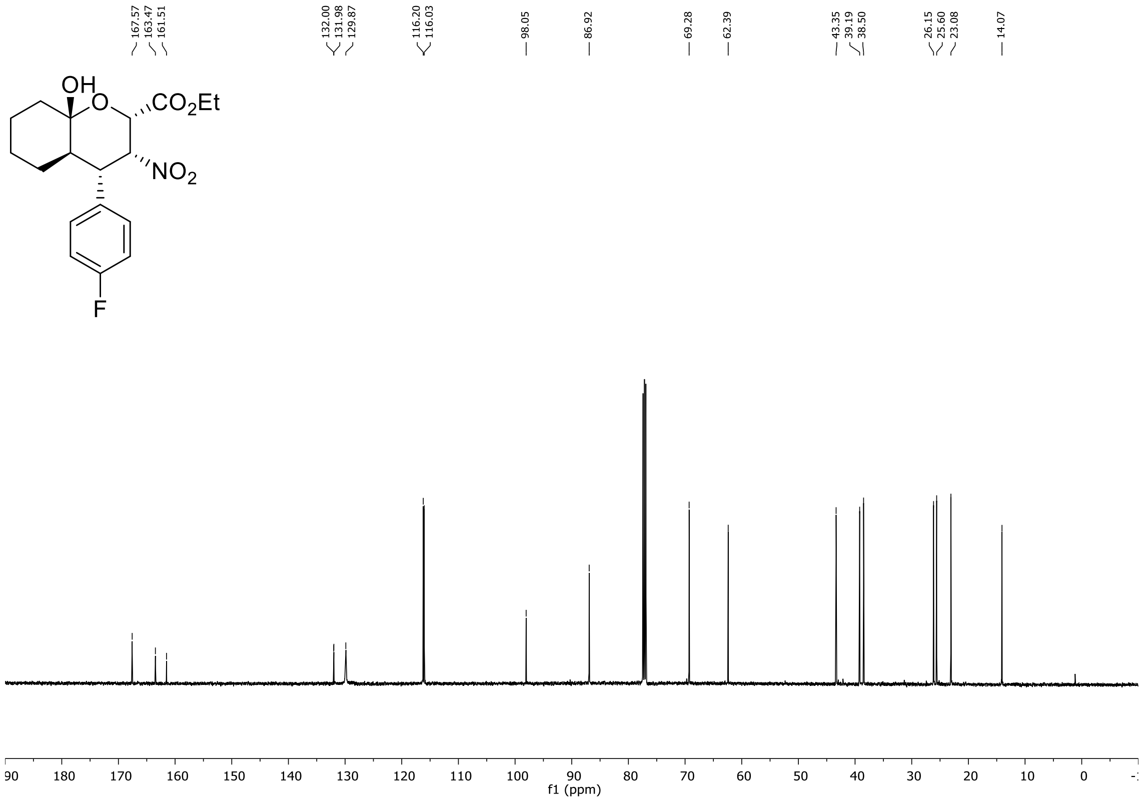

^{19}F NMR (376 MHz, $CDCl_3$) of 10aca

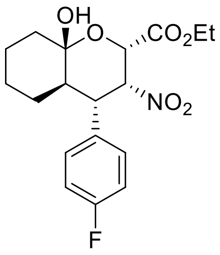

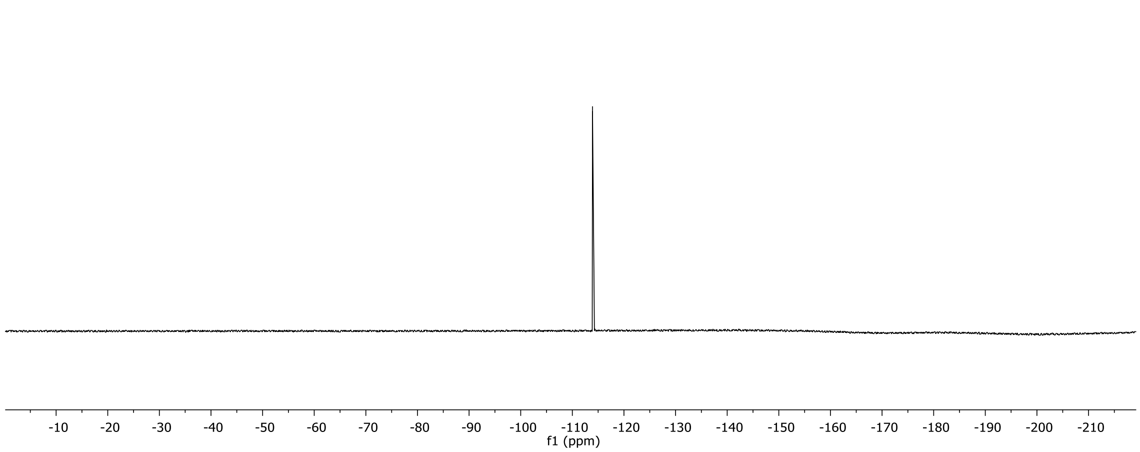

Compound **10ada**

^{1}H NMR (500 MHz, $CDCl_3$) of 10ada

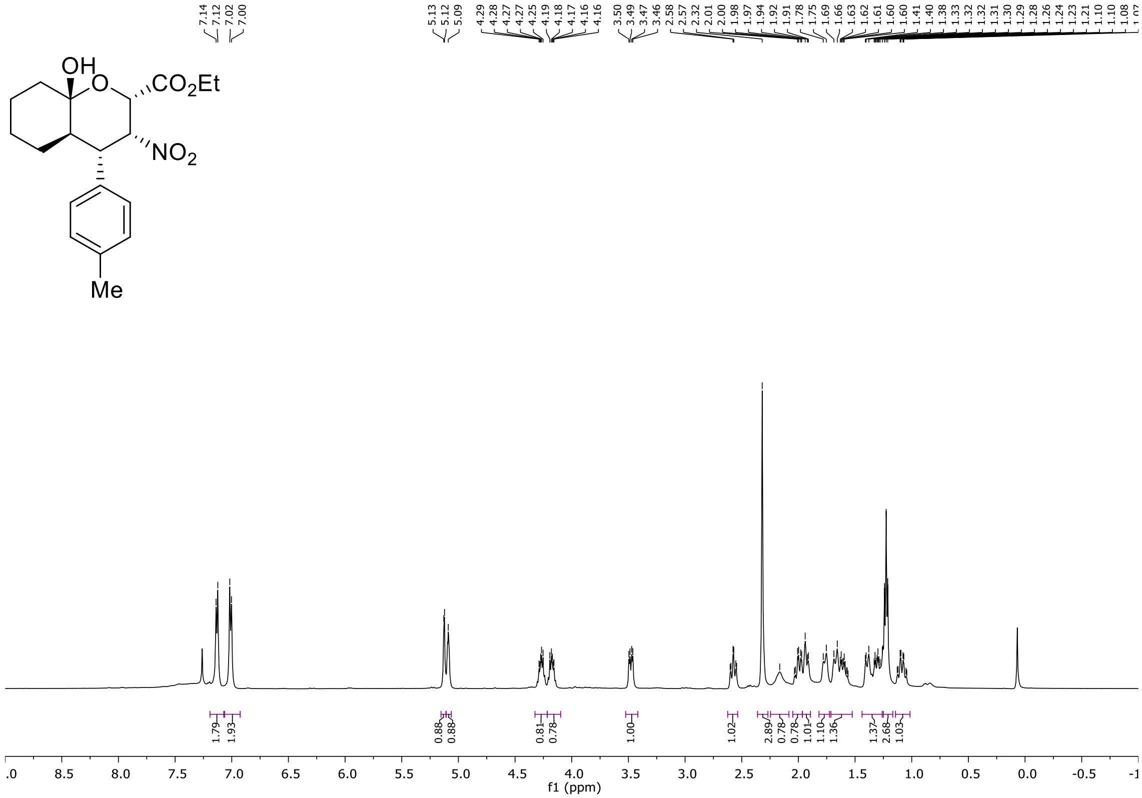

^{13}C{H} NMR (126 MHz, $CDCl_3$) of 10ada

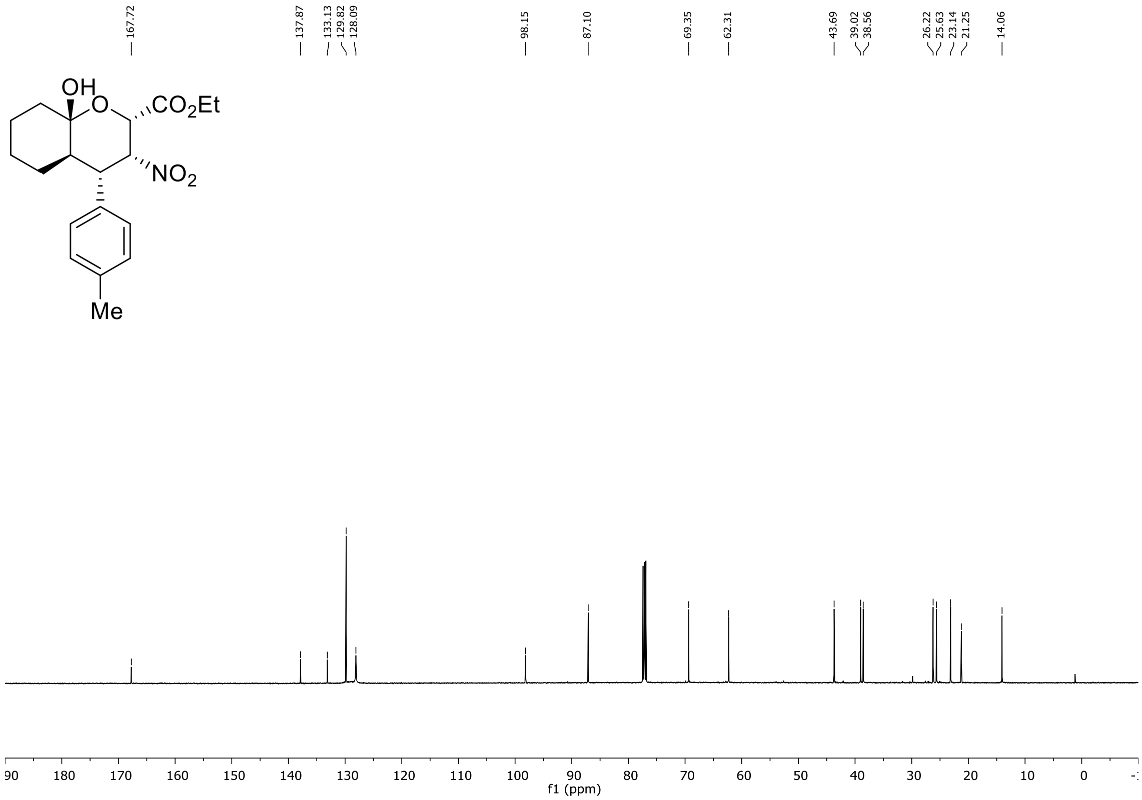

Compound **10aea**

^{1}H NMR (400 MHz, $CDCl_3$) of 10aea

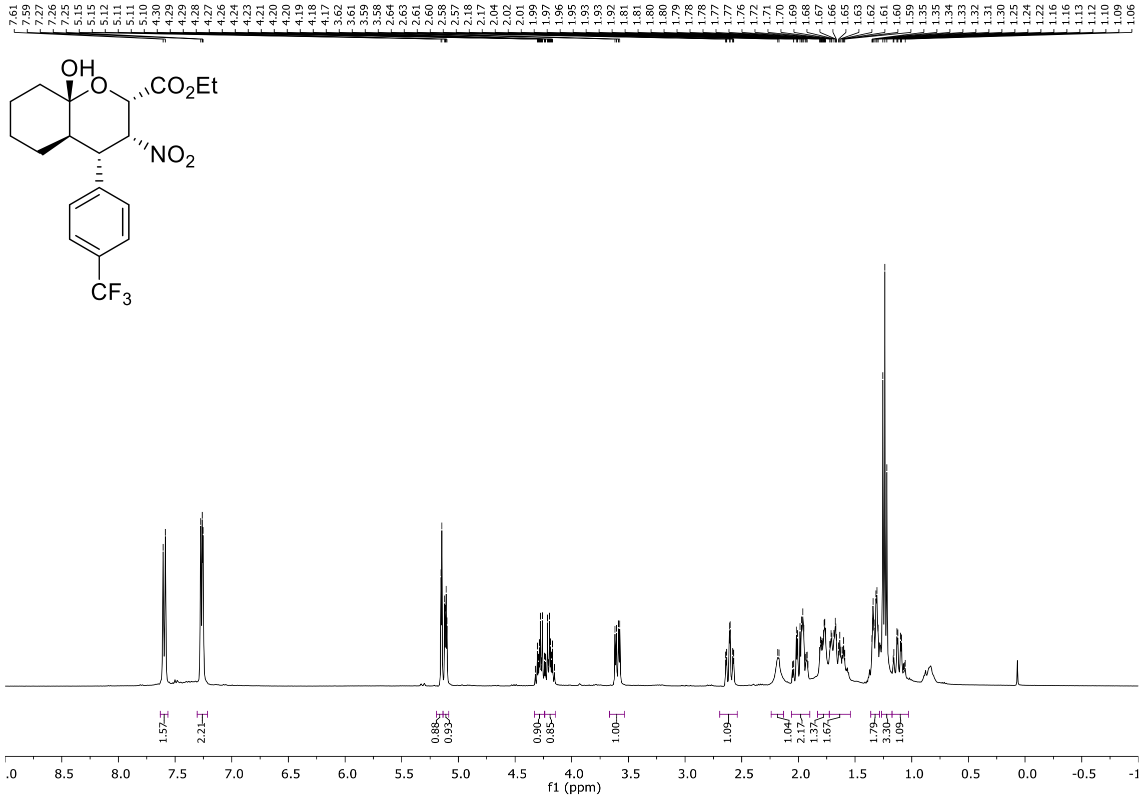

$^{13}C\{H\}$ NMR (126 MHz, $CDCl_3$) of 10aea

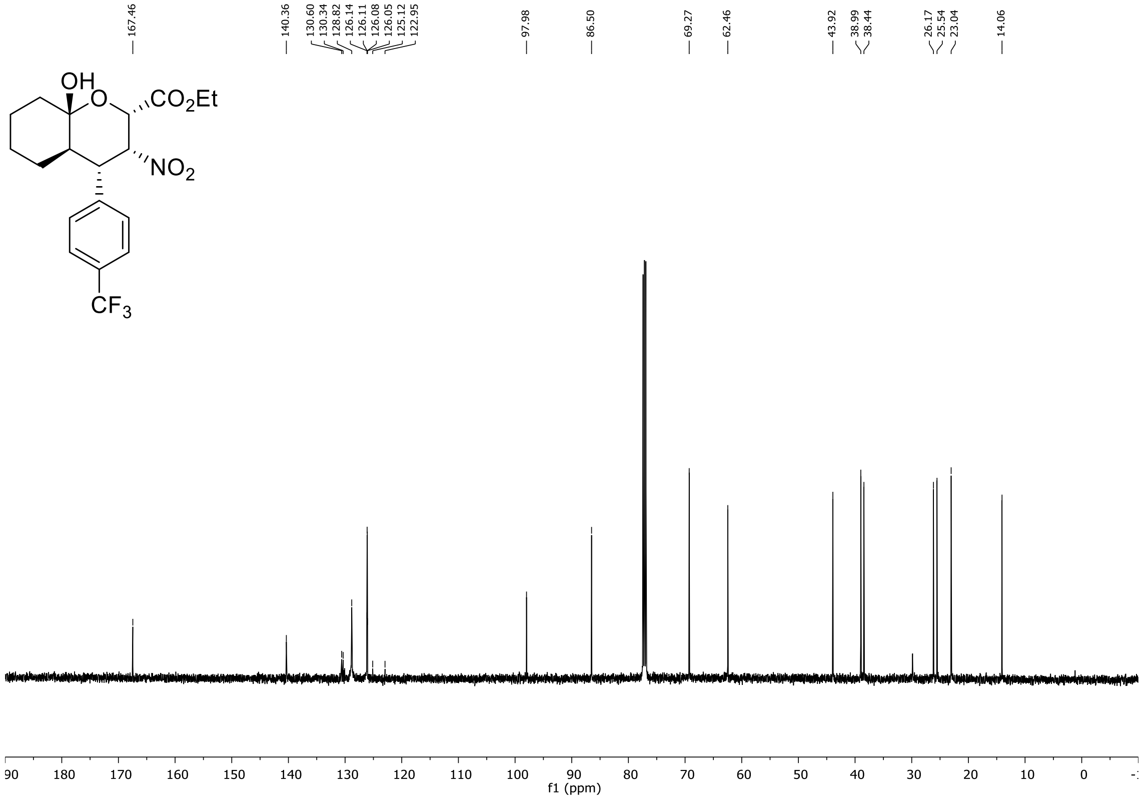

^{19}F NMR (376 MHz, $CDCl_3$) of 10aea

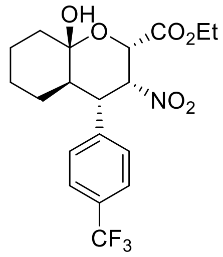

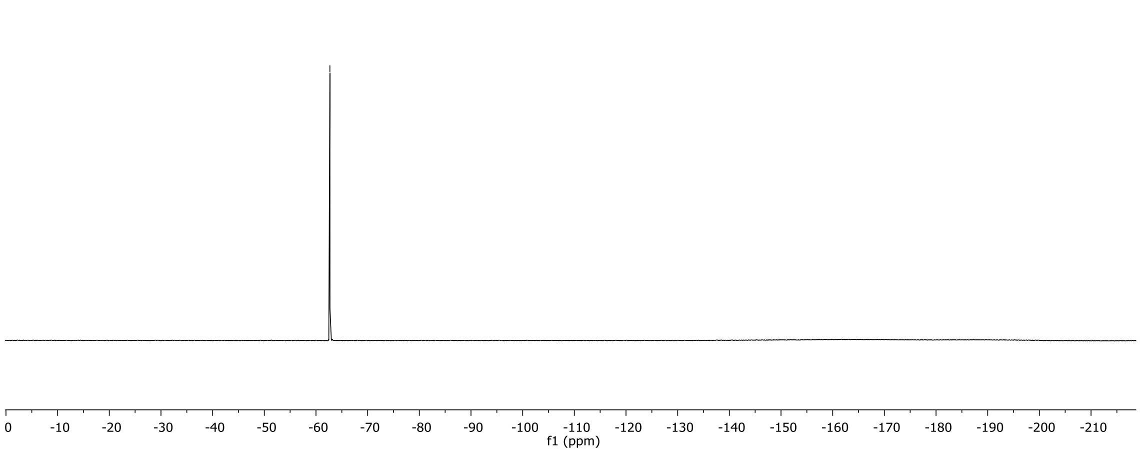

Compound **10afa**

^{1}H NMR (400 MHz, $CDCl_3$) of 10afa

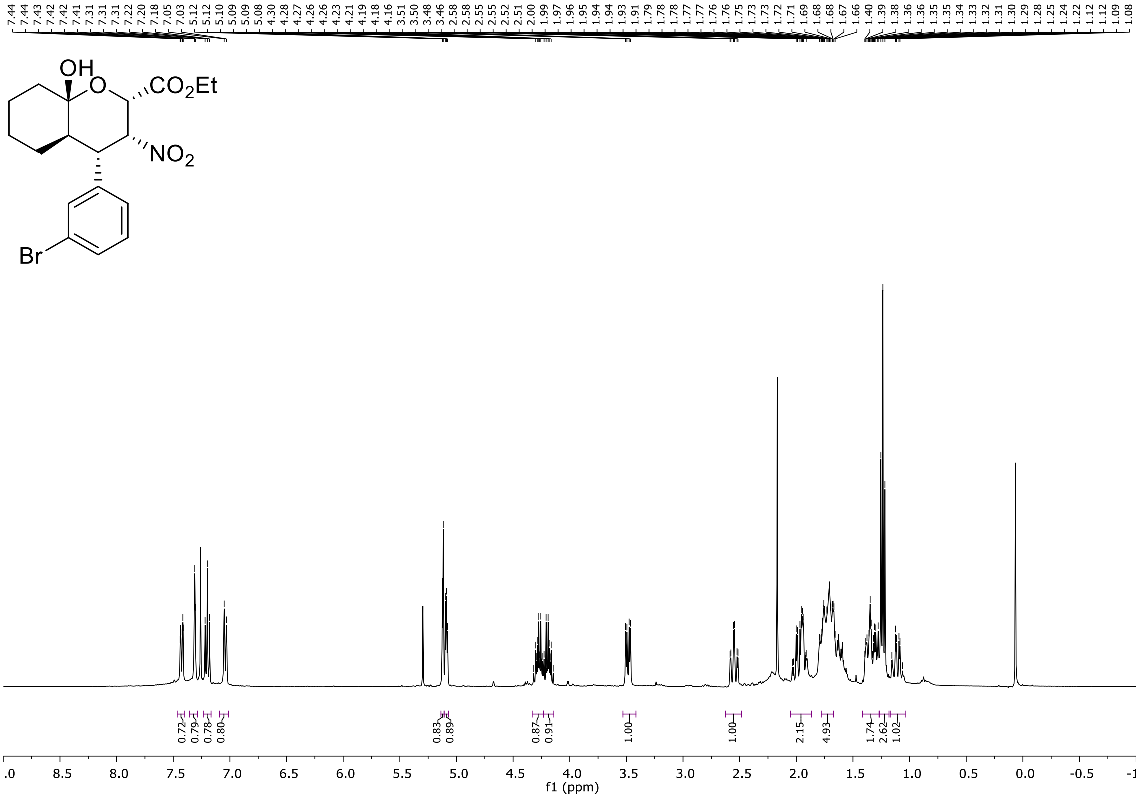

$^{13}C\{H\}$ NMR (126 MHz, $CDCl_3$) of 10afa

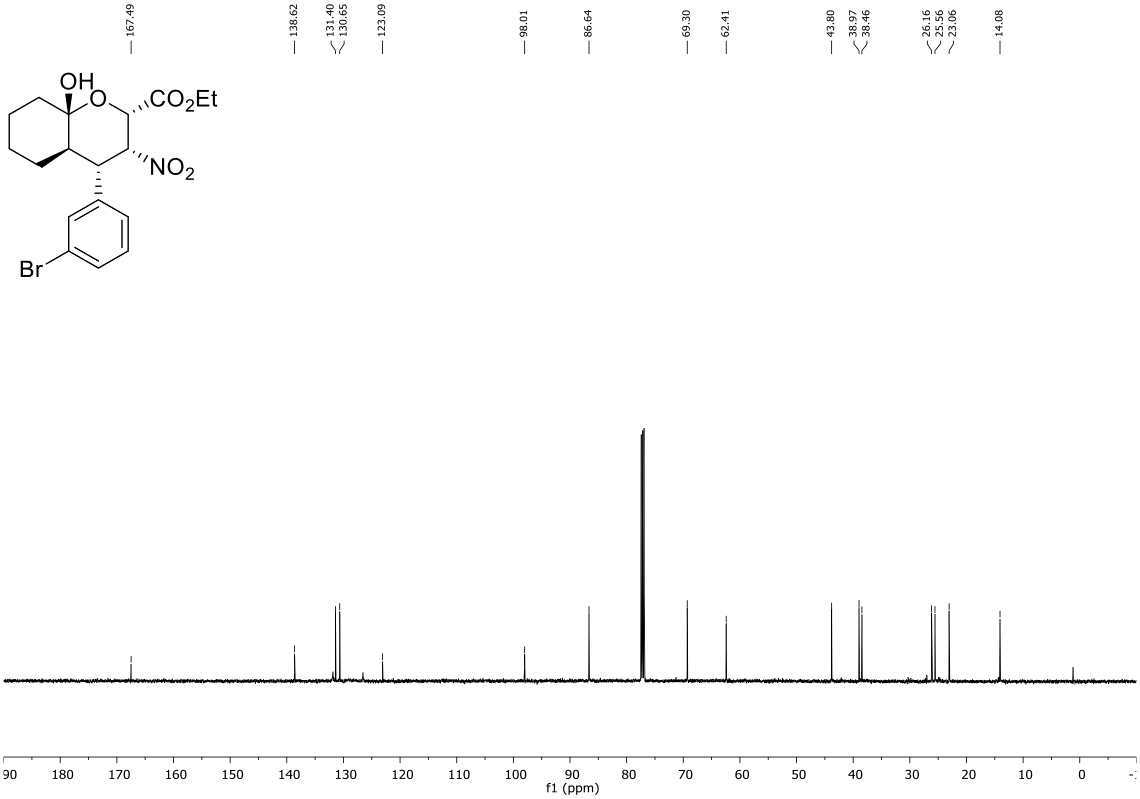

Compound **10aga**

^{1}H NMR (400 MHz, $CDCl_3$) of 10aga

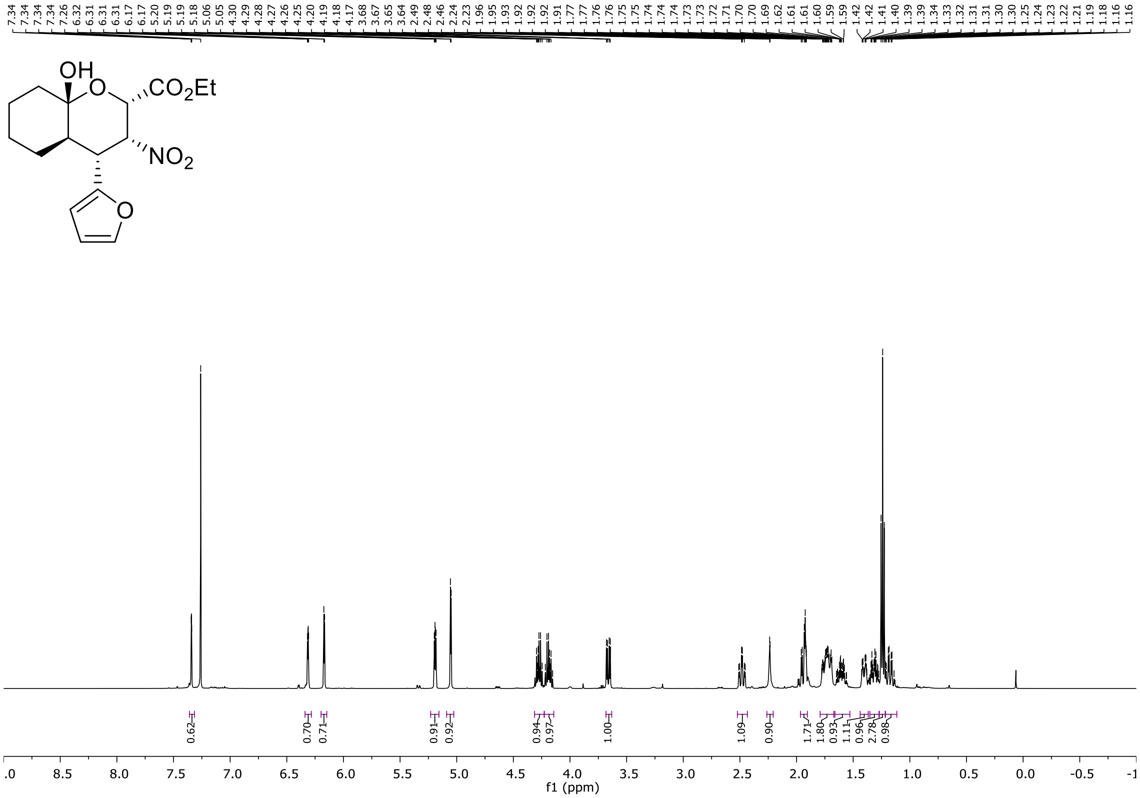

^{13}C{H} NMR (126 MHz, $CDCl_3$) of 10aga

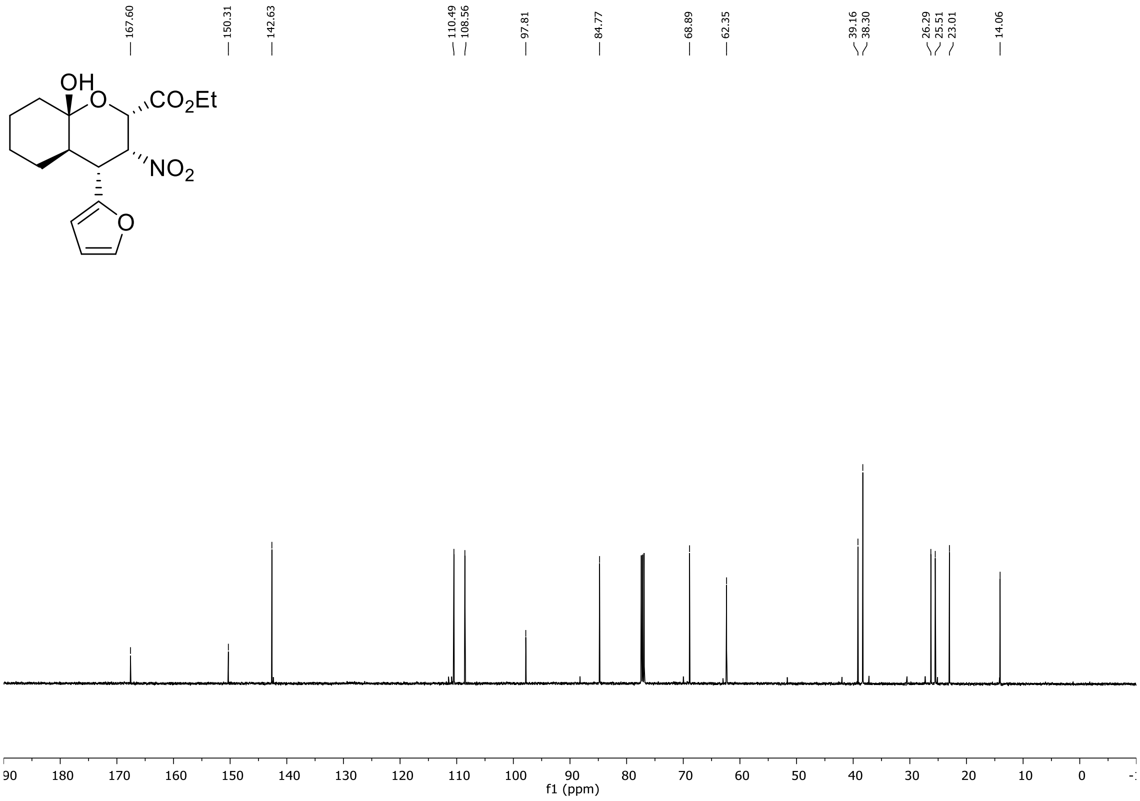

Compound **10aha**

^{1}H NMR (400 MHz, $CDCl_3$) of 10aha

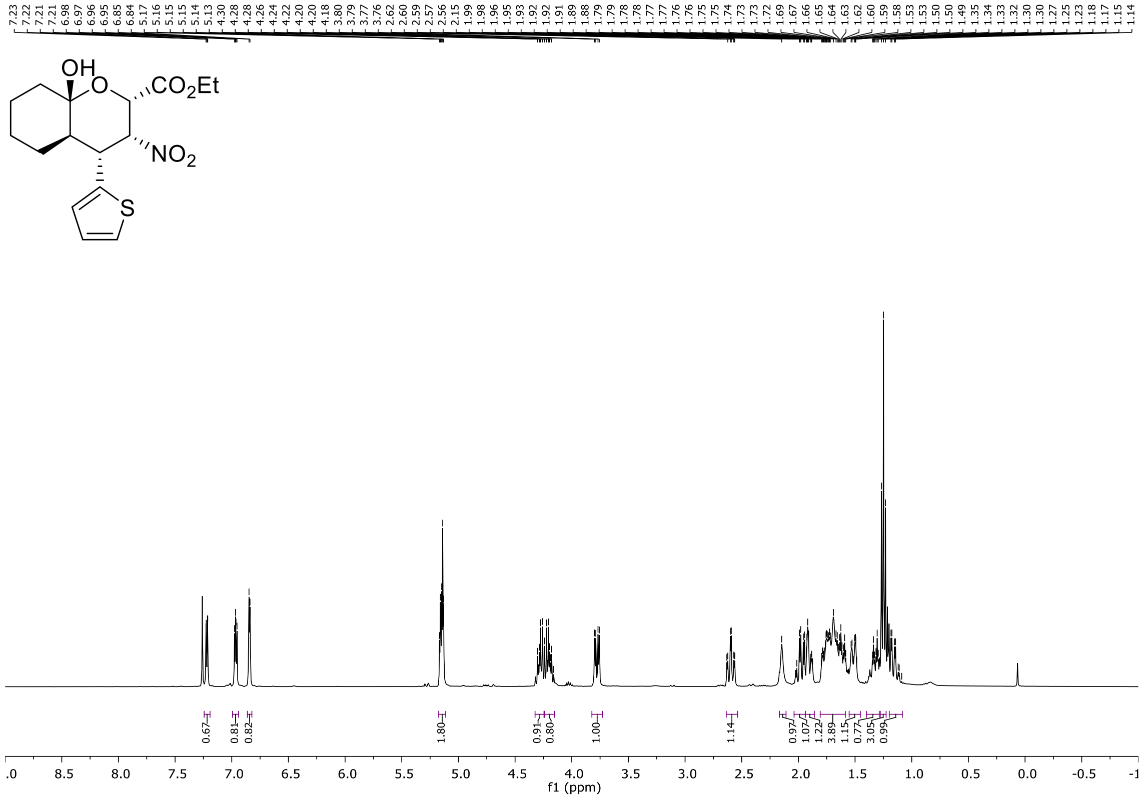

^{13}C{H} NMR (126 MHz, $CDCl_3$) of 10aha

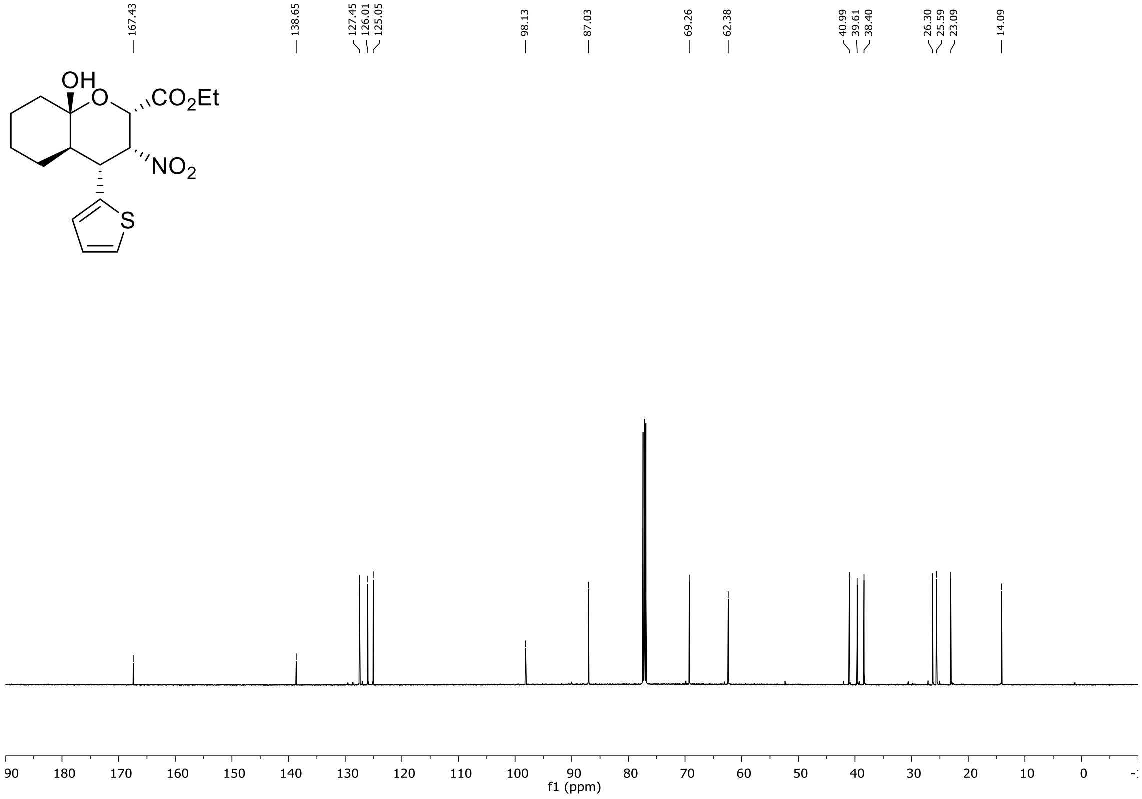

Compound **10aia**

^{1}H NMR (400 MHz, $CDCl_3$) of 10aia

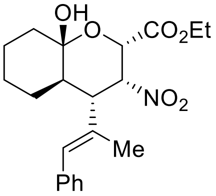

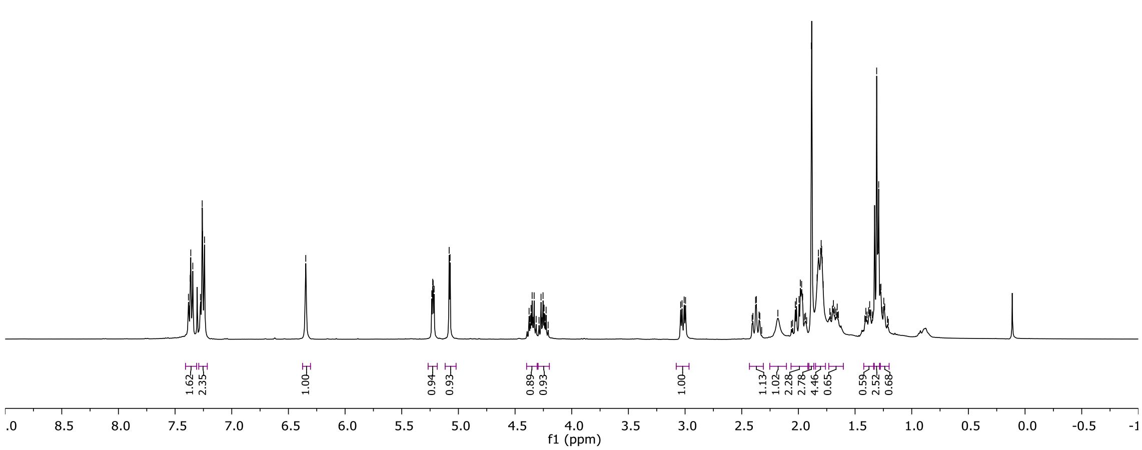

$^{13}C\{H\}$ NMR (126 MHz, $CDCl_3$) of 10aia

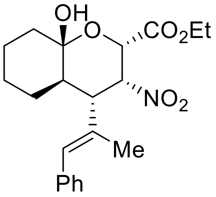

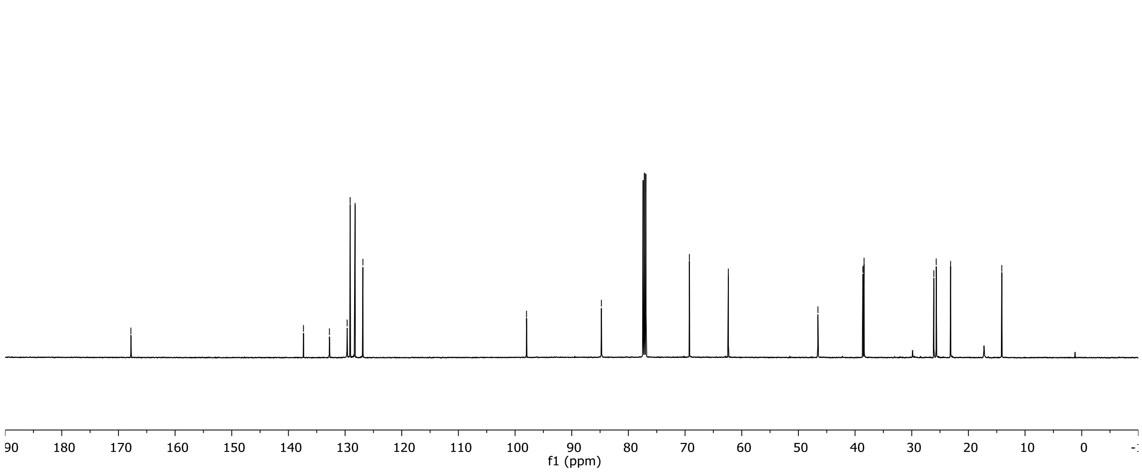

Compound **10baa**

^{1}H NMR (400 MHz, $CDCl_3$) of 10baa

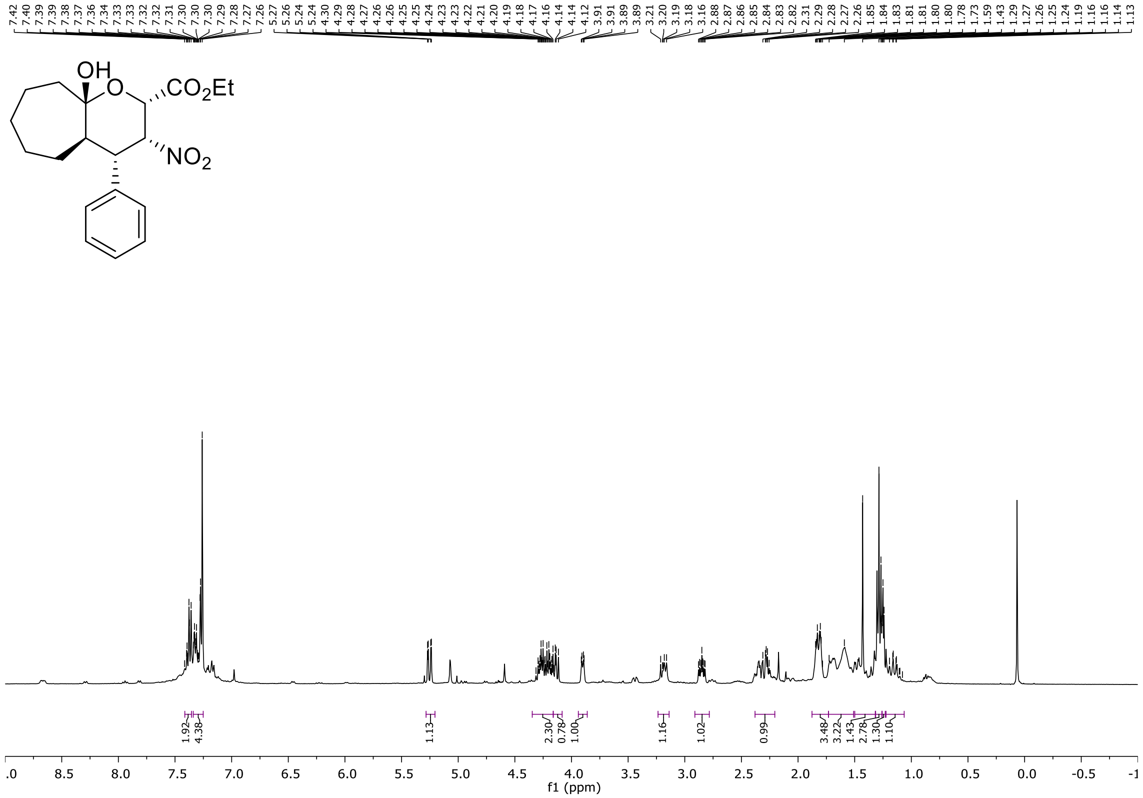

^{13}C{H} NMR (101 MHz, $CDCl_3$) of 10baa

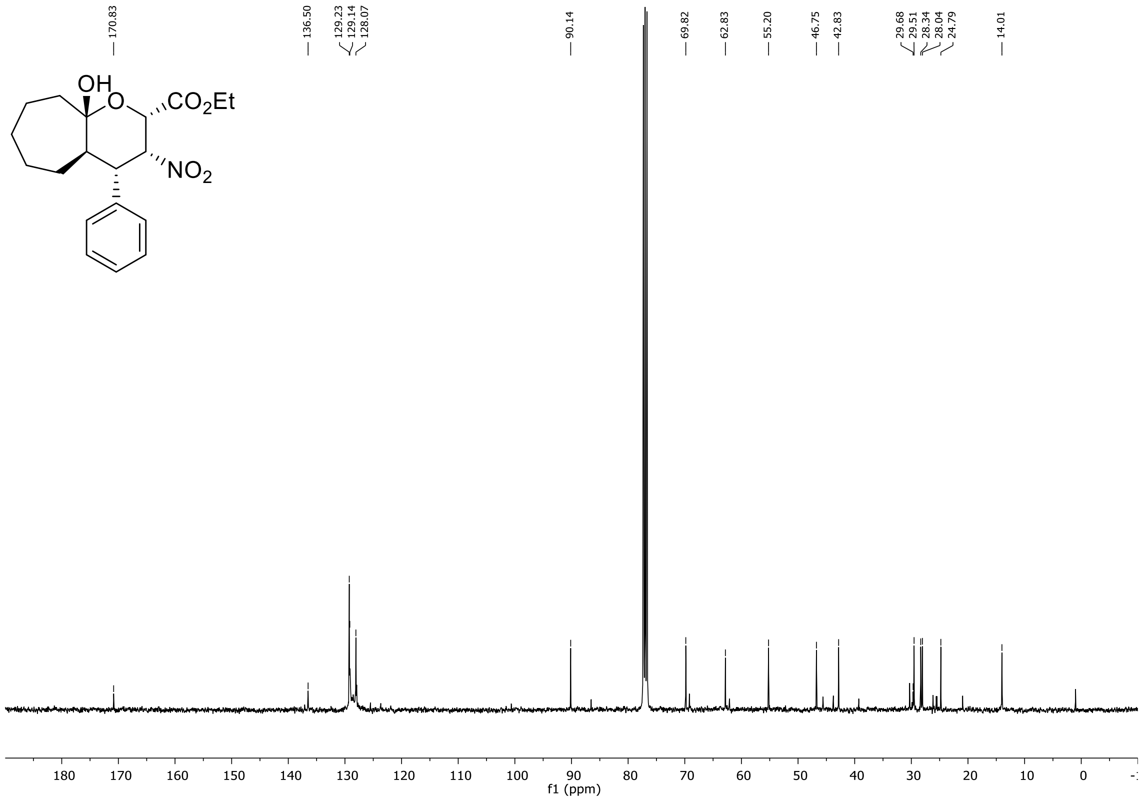

Compound **10caa**

^{1}H NMR (400 MHz, $CDCl_3$) of 10caa

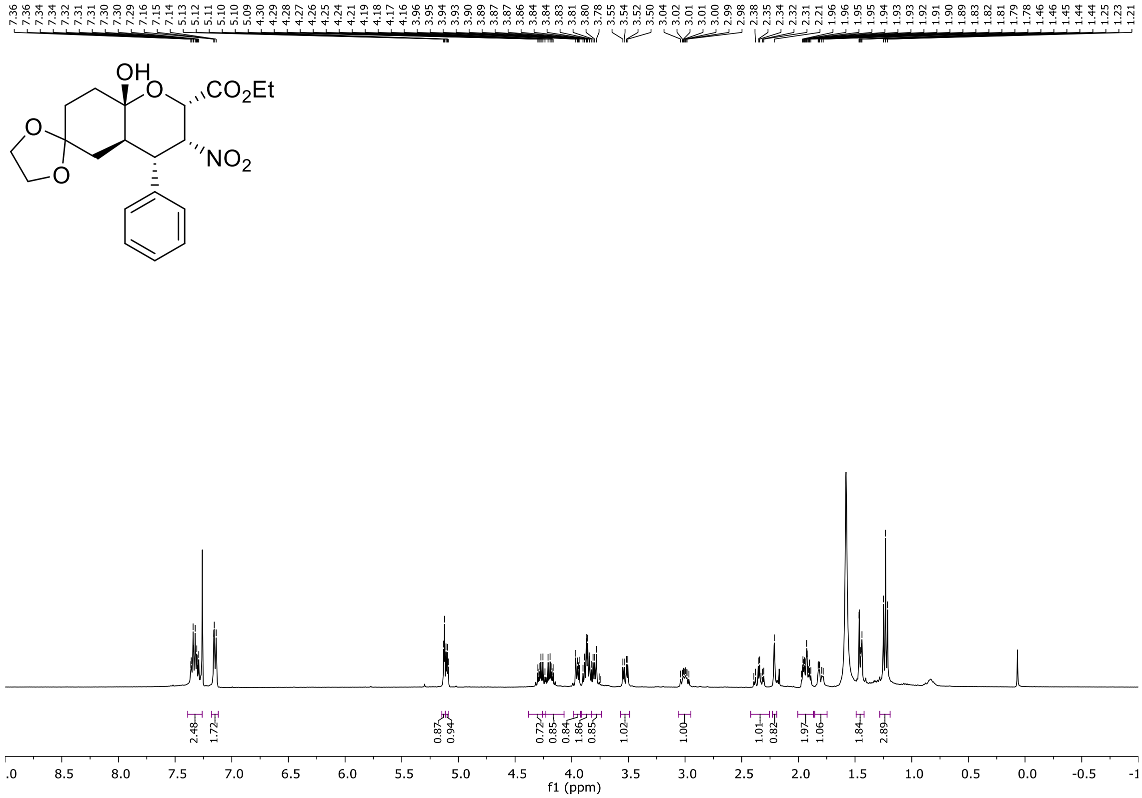

^{13}C{H} NMR (126 MHz, $CDCl_3$) of 10caa

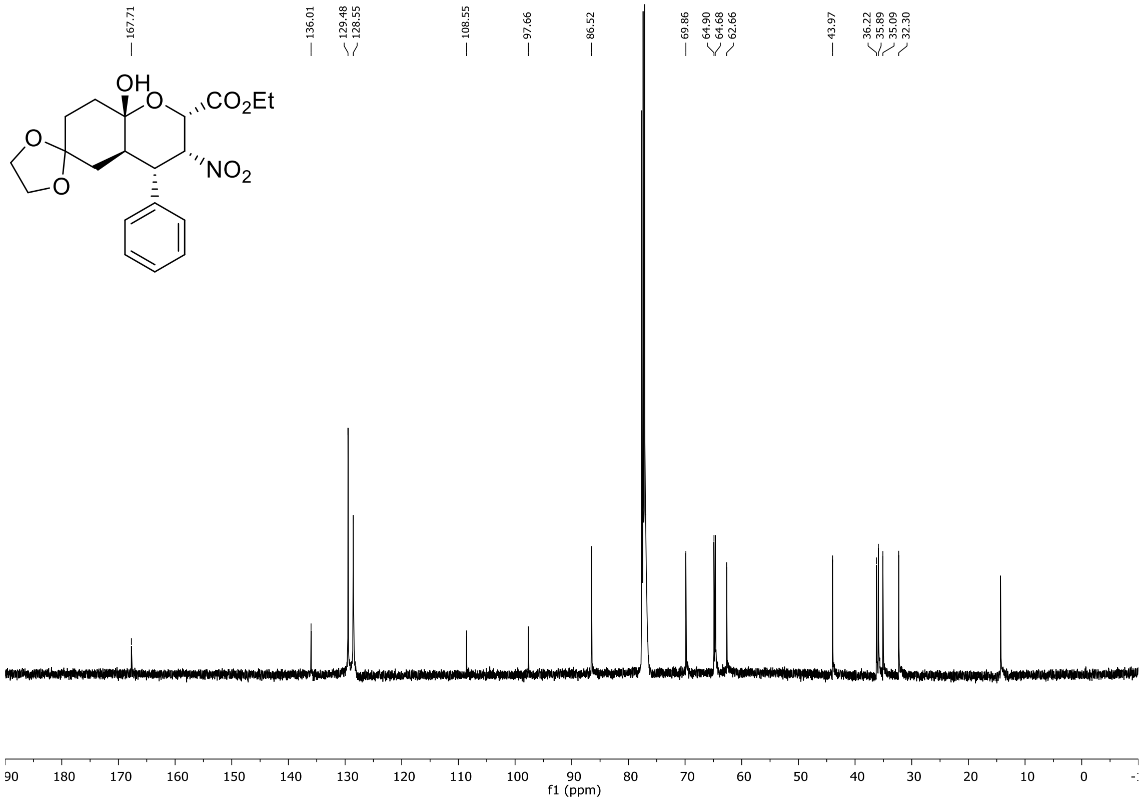

Compound **10aab**

^{1}H NMR (500 MHz, $CDCl_3$) of 10aab

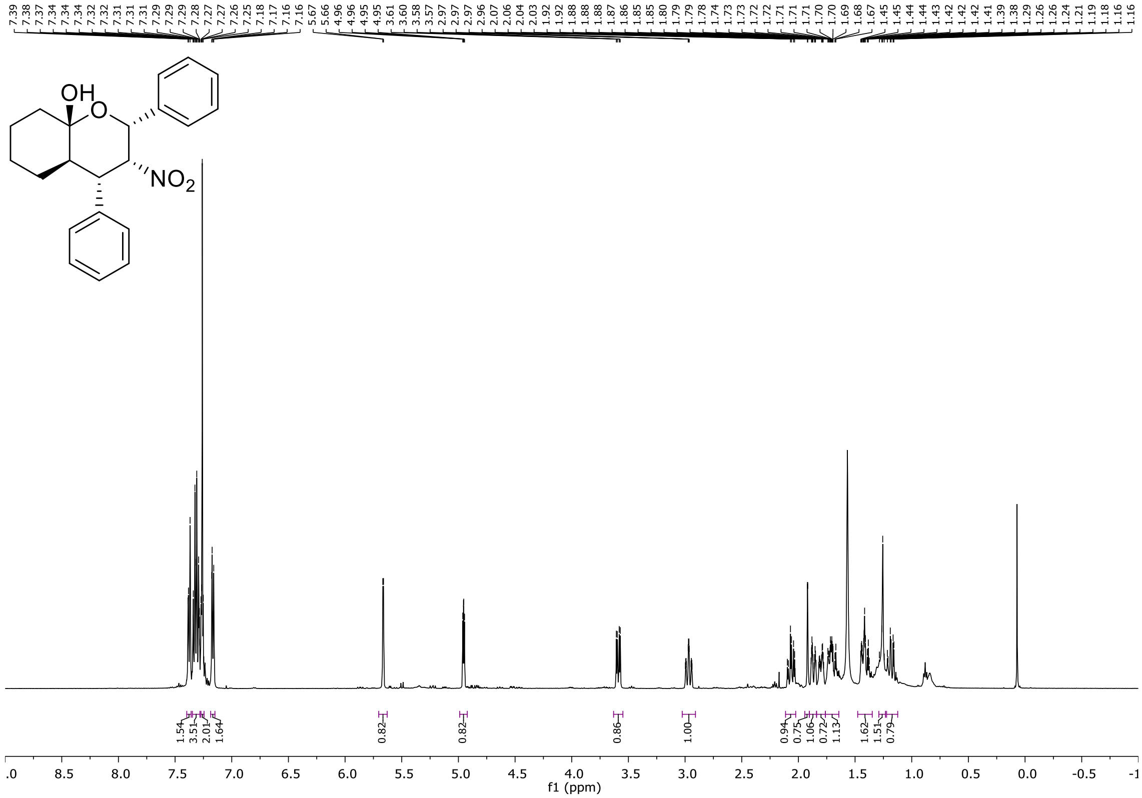

^{13}C{H} NMR (126 MHz, $CDCl_3$) of 10aab

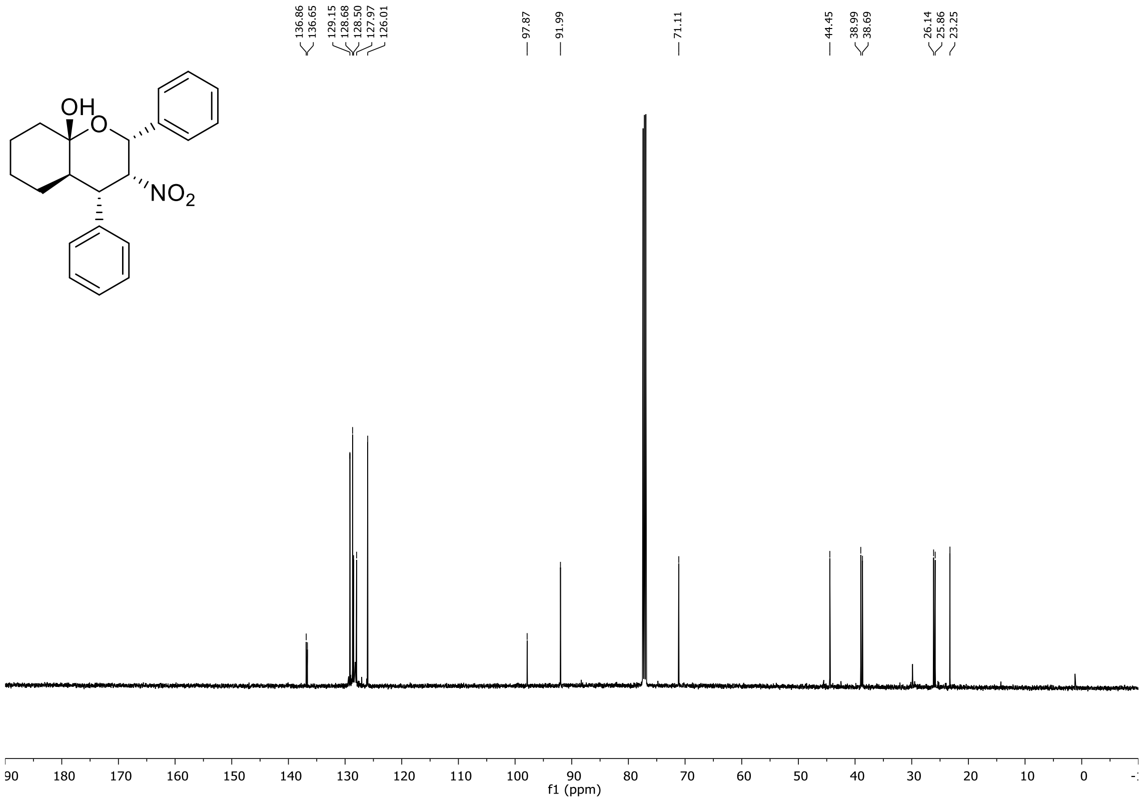

Compound **10aab'**

^{1}H NMR (400 MHz, CDCl$_3$) of 10aab'

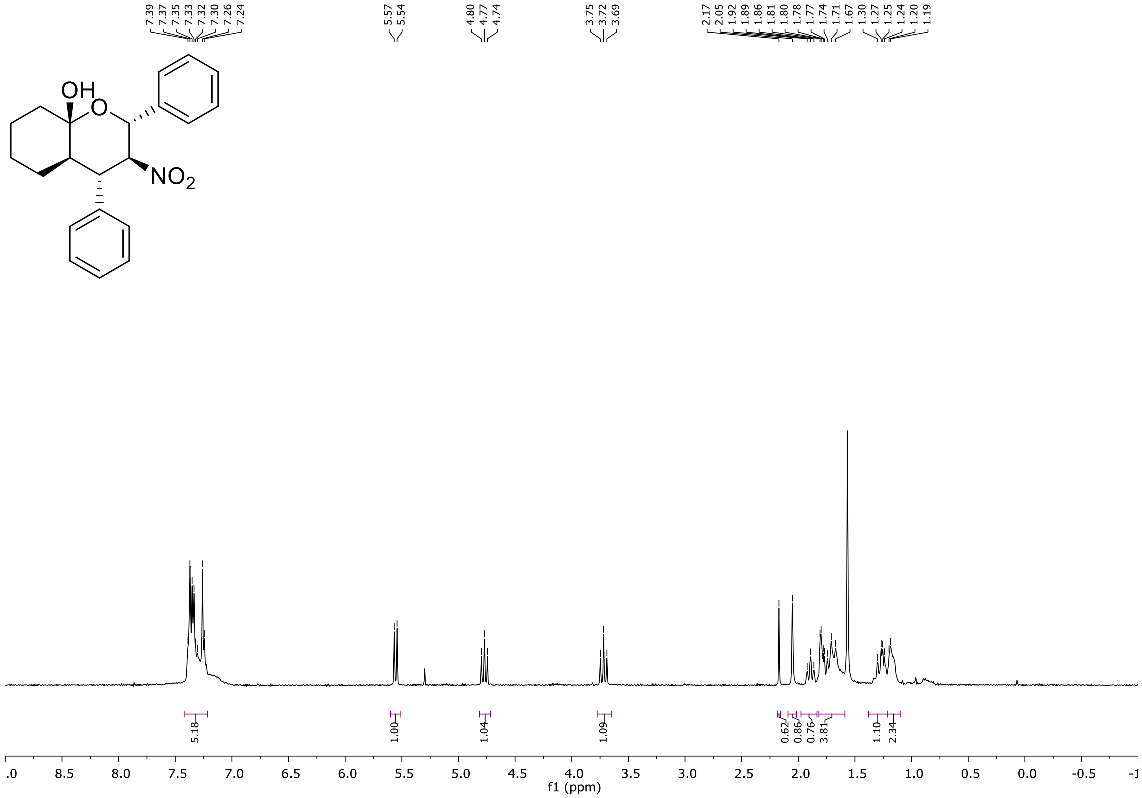

^{13}C{H} NMR (126 MHz, CDCl$_3$) of 10aab'

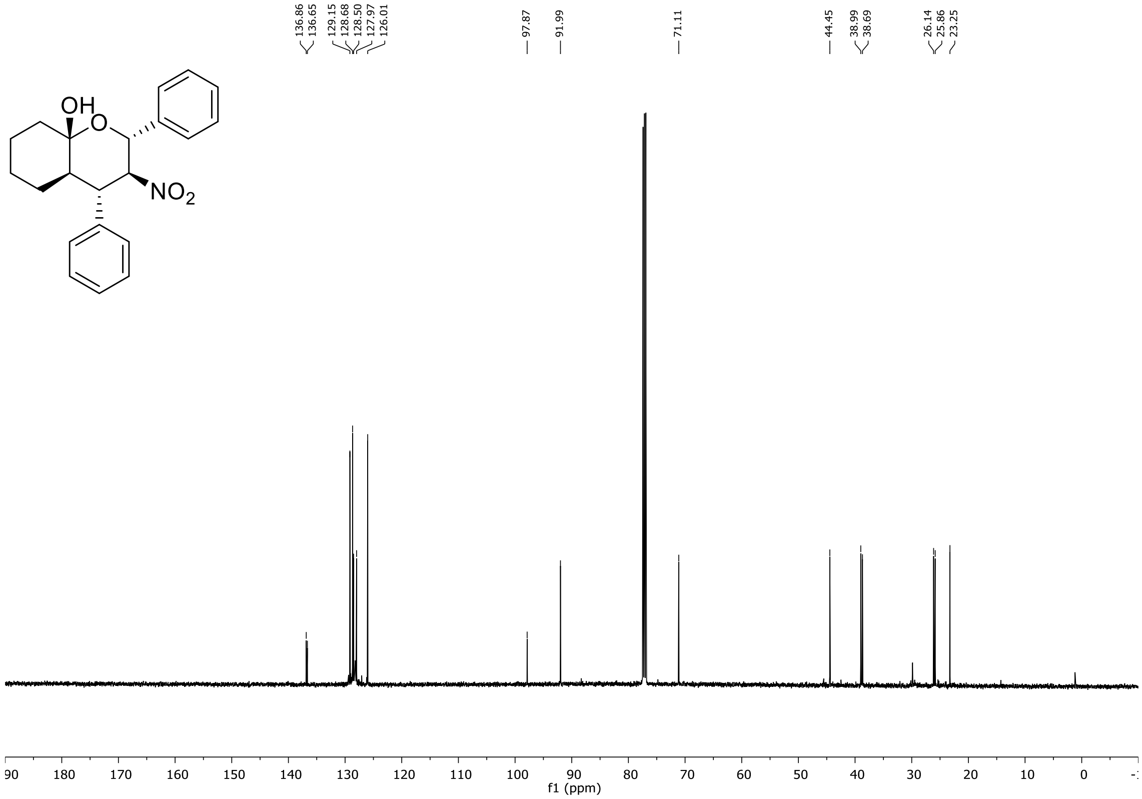

Compound **10aac**

^{1}H NMR (400 MHz, $CDCl_3$) of 10aac

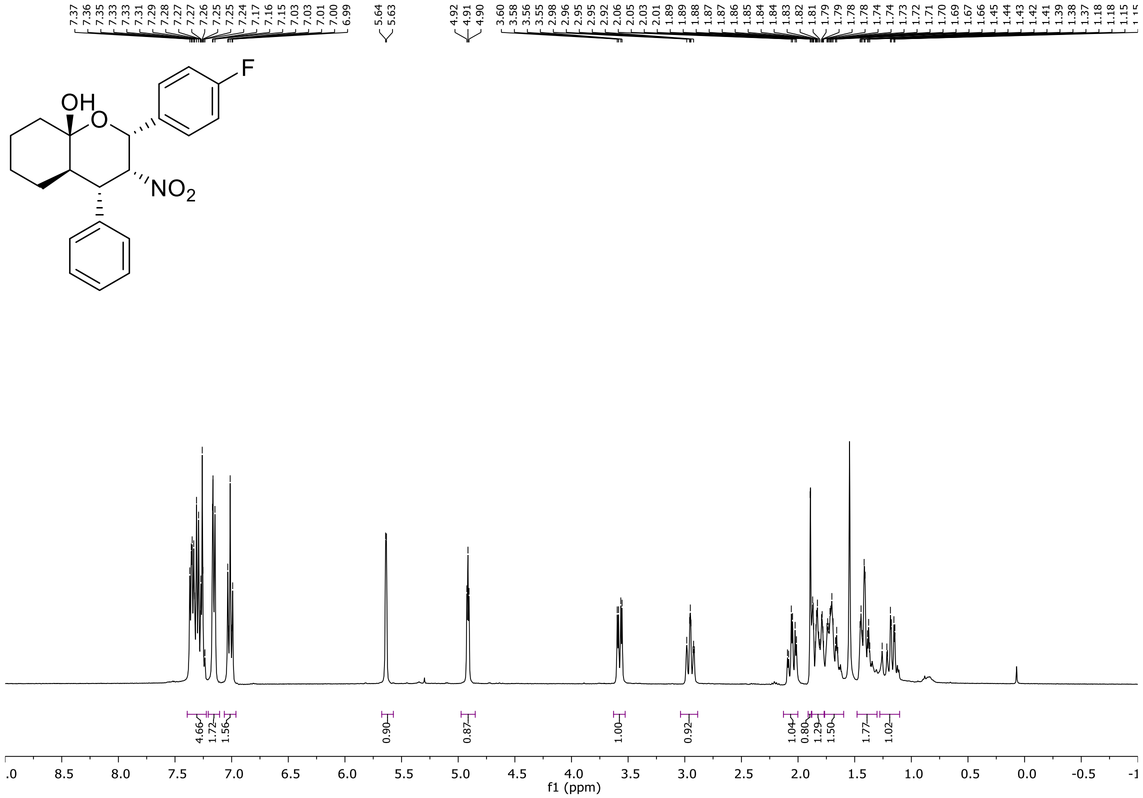

^{13}C{H} NMR (126 MHz, $CDCl_3$) of 10aac

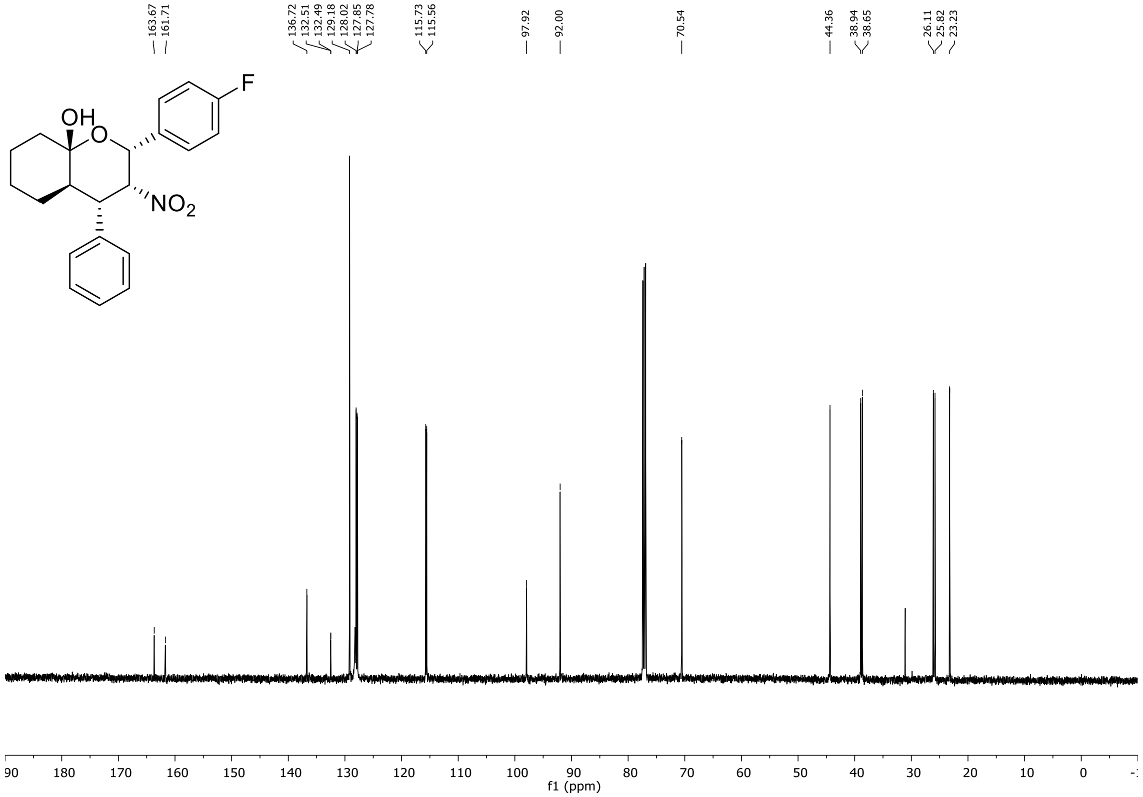

^{19}F NMR (376 MHz, $CDCl_3$) of 10aac

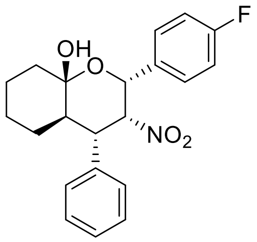

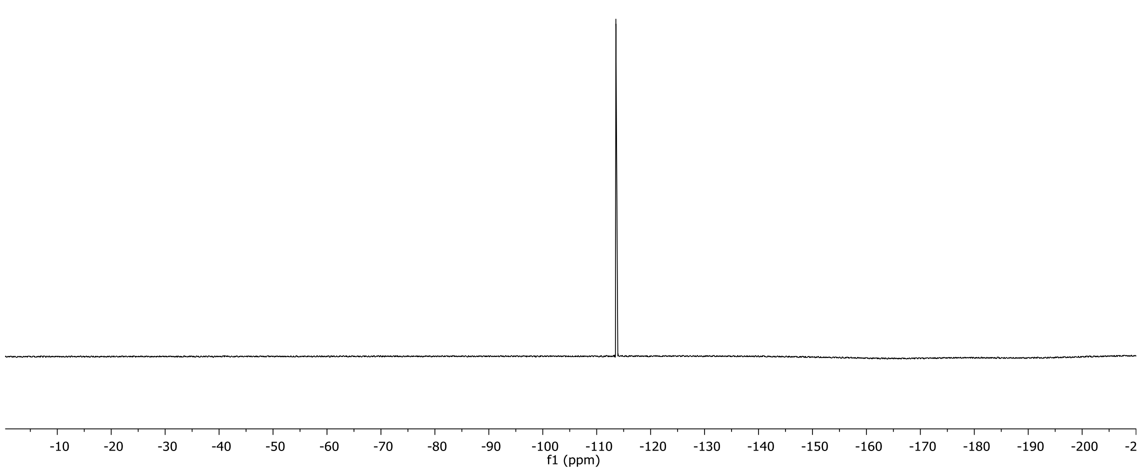

Compound **10aac’**

^{1}H NMR (400 MHz, $CDCl_3$) of 10aac’

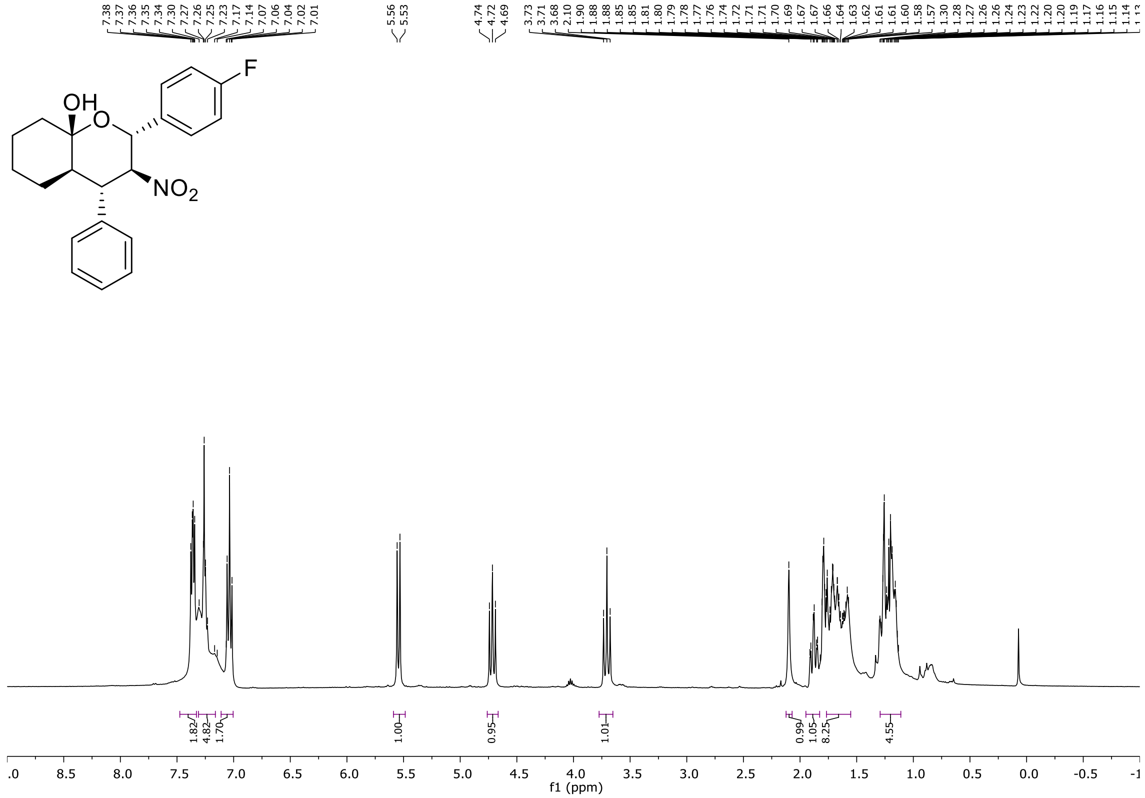

^{13}C{H} NMR (126 MHz, $CDCl_3$) of 10aac’

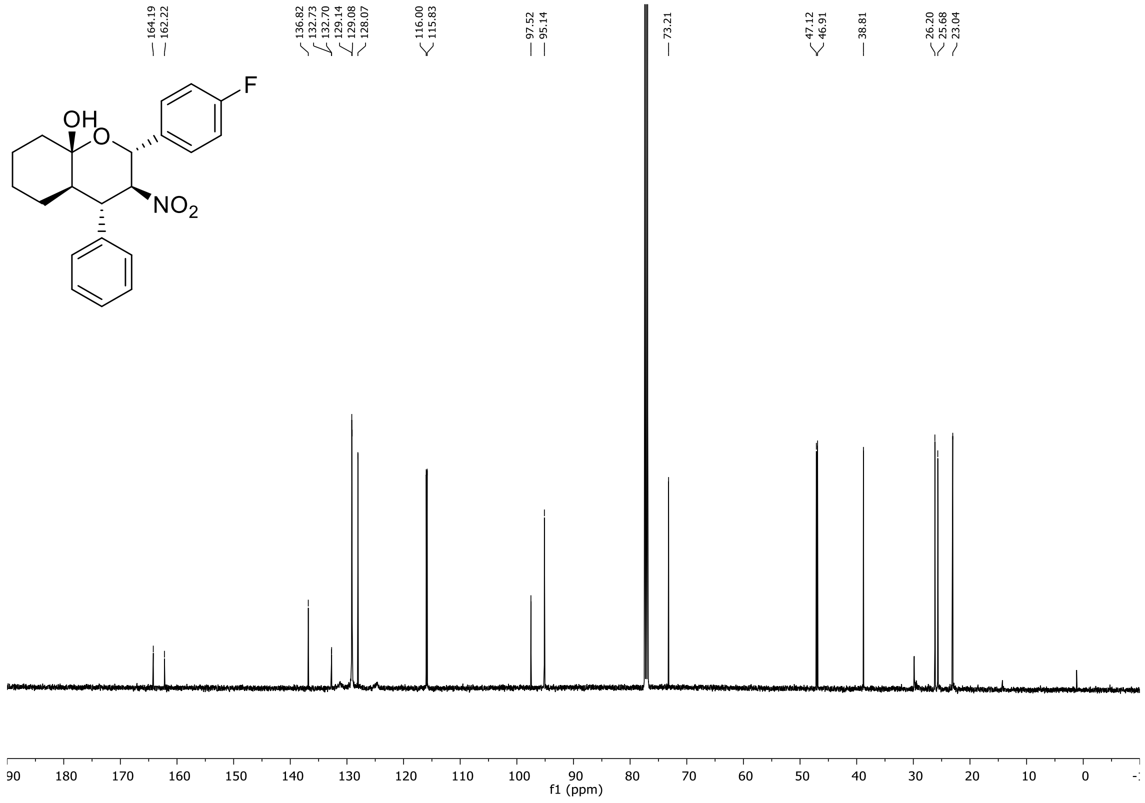

^{19}F NMR (376 MHz, $CDCl_3$) of 10aac’

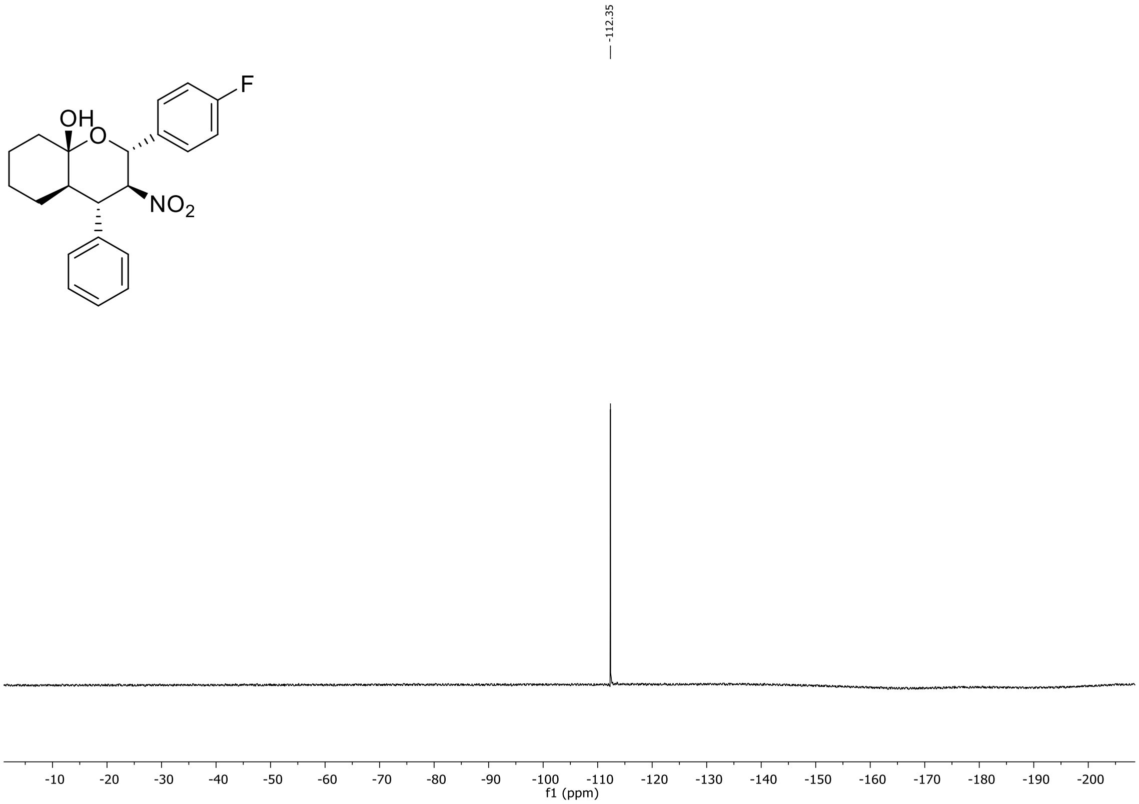

Compound **10aad’**

^{1}H NMR (500 MHz, $CDCl_3$) of 10aad’

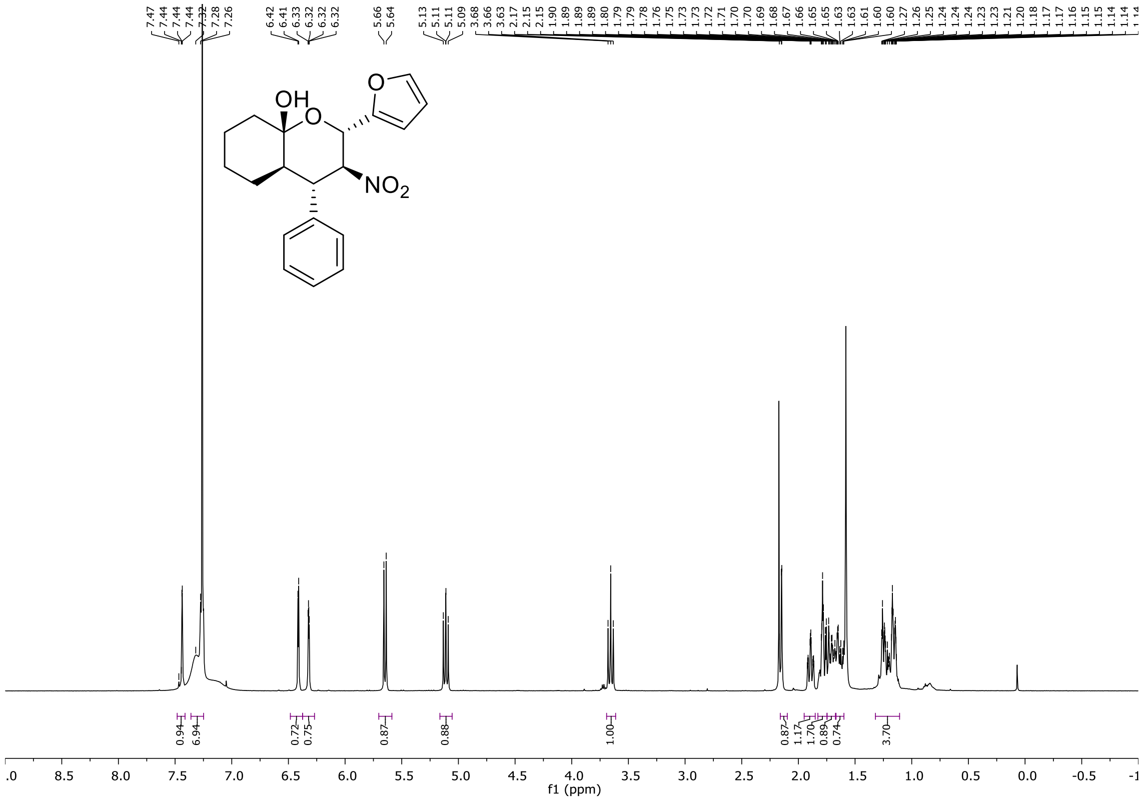

^{13}C{H} NMR (126 MHz, $CDCl_3$) of 10aad’

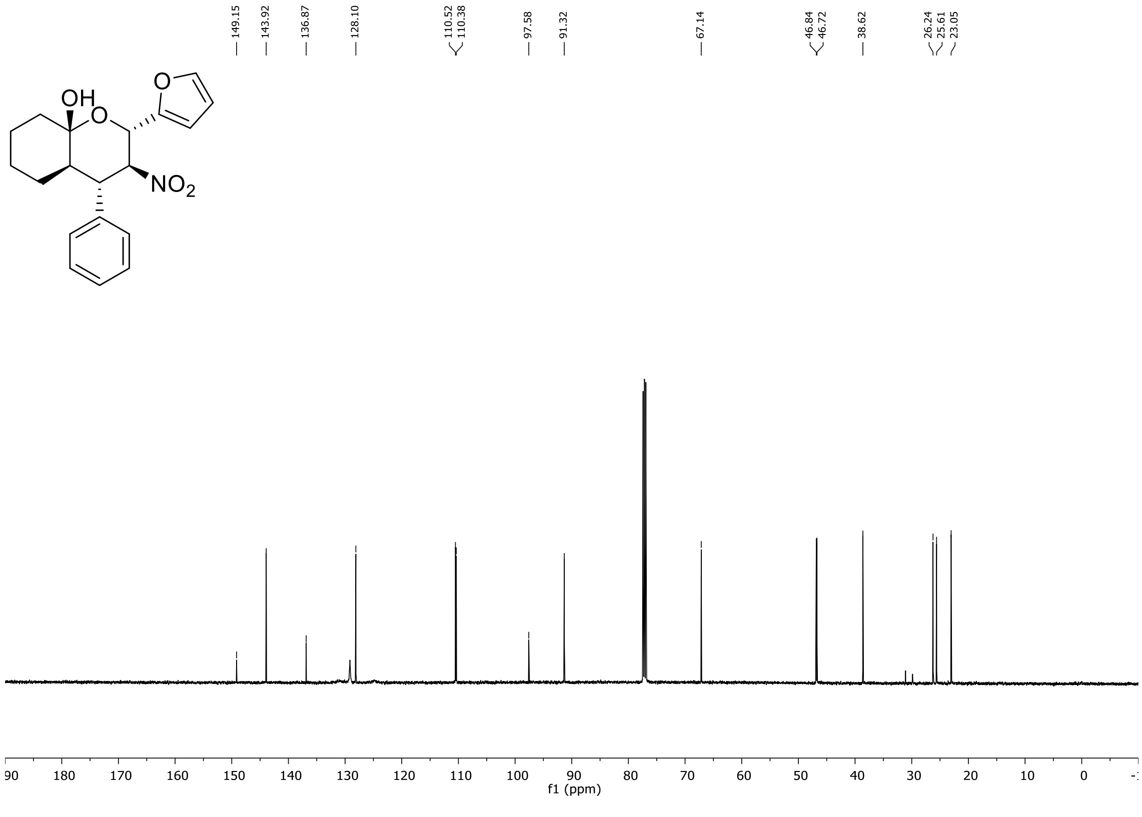

Compound **10aae**

^{1}H NMR (500 MHz, $CDCl_3$) of 10aae

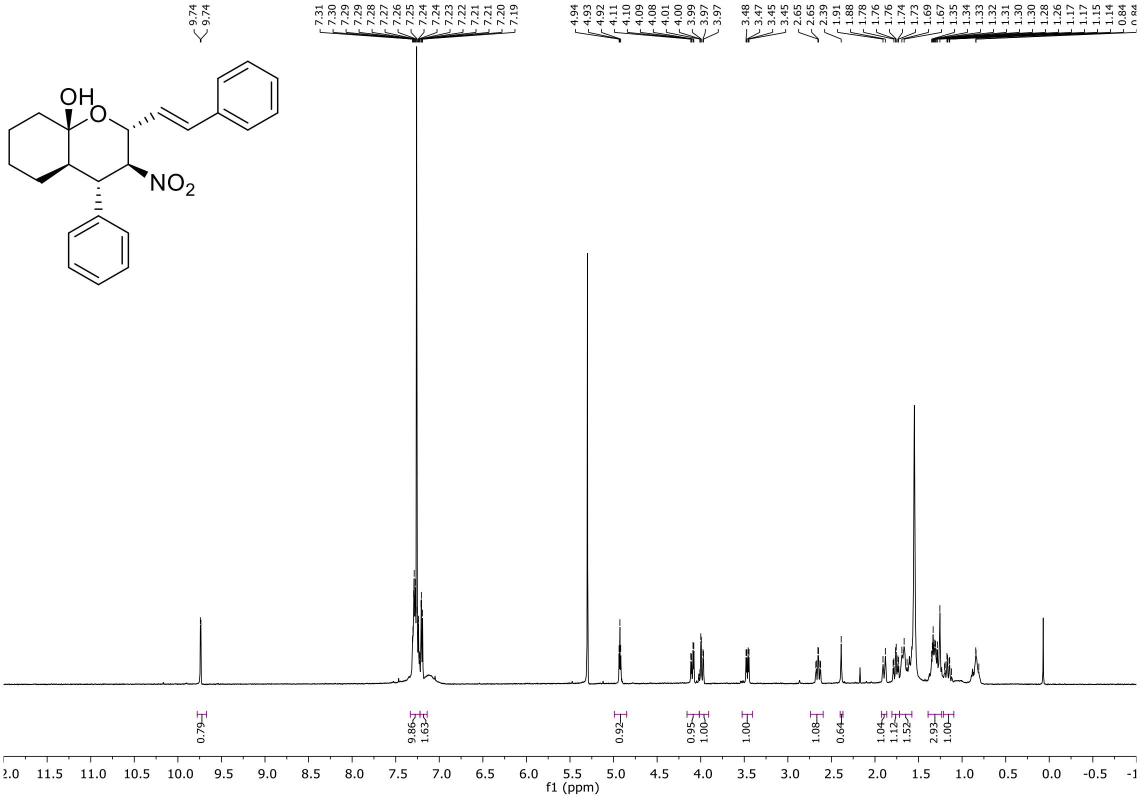

$^{13}C\{H\}$ NMR (126 MHz, $CDCl_3$) of 10aae

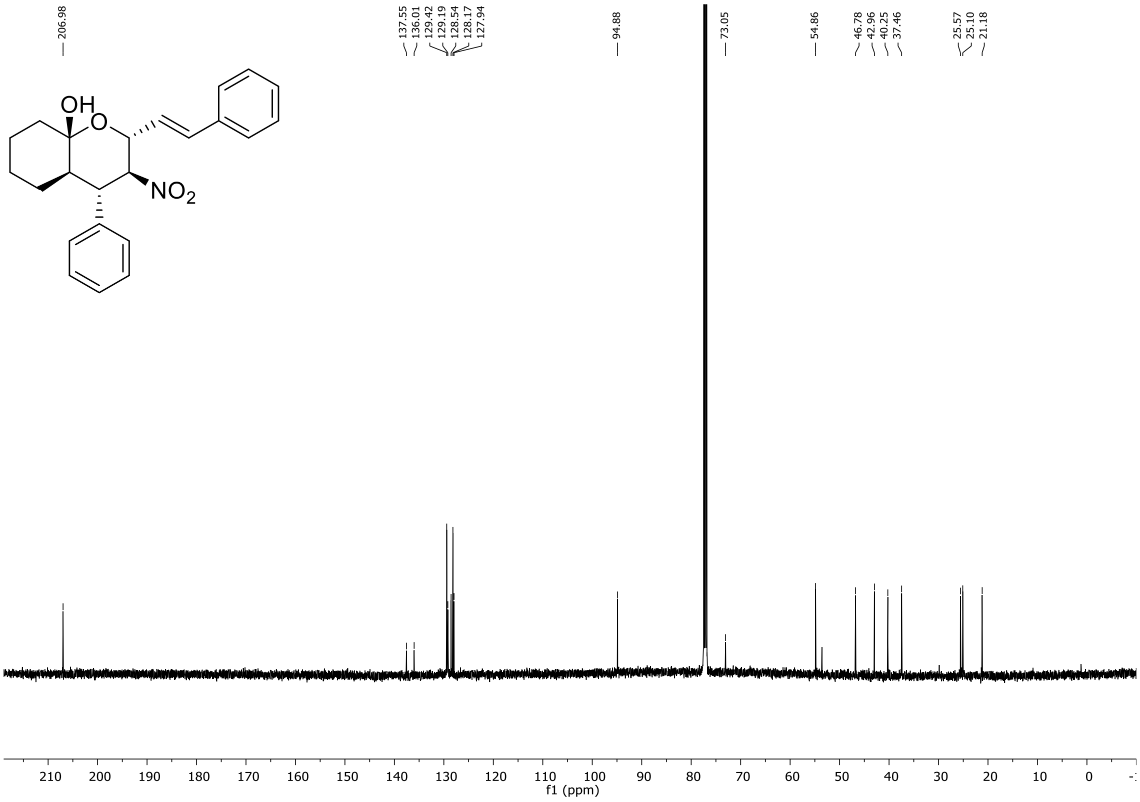

COSY ($CDCl_3$) of 10aae

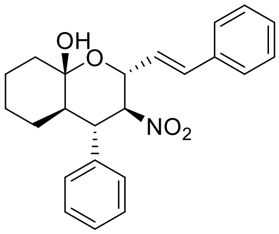

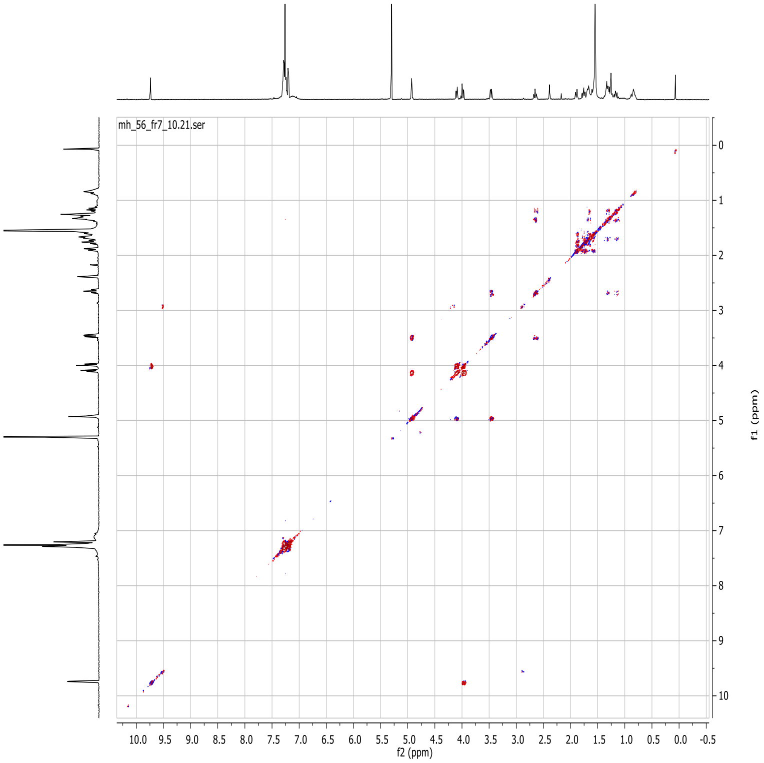

Compound **10aaf**

^{1}H NMR (400 MHz, $CDCl_3$) of 10aaf

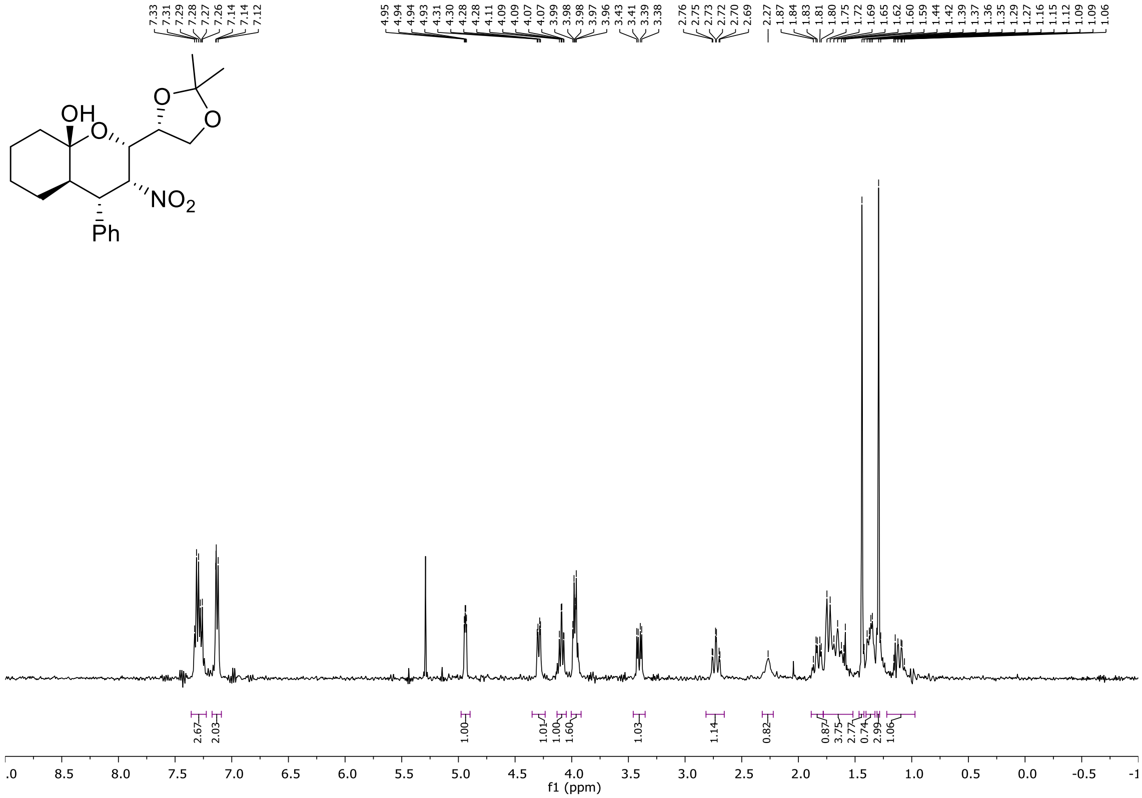

$^{13}C\{H\}$ NMR (126 MHz, $CDCl_3$) of 10aaf

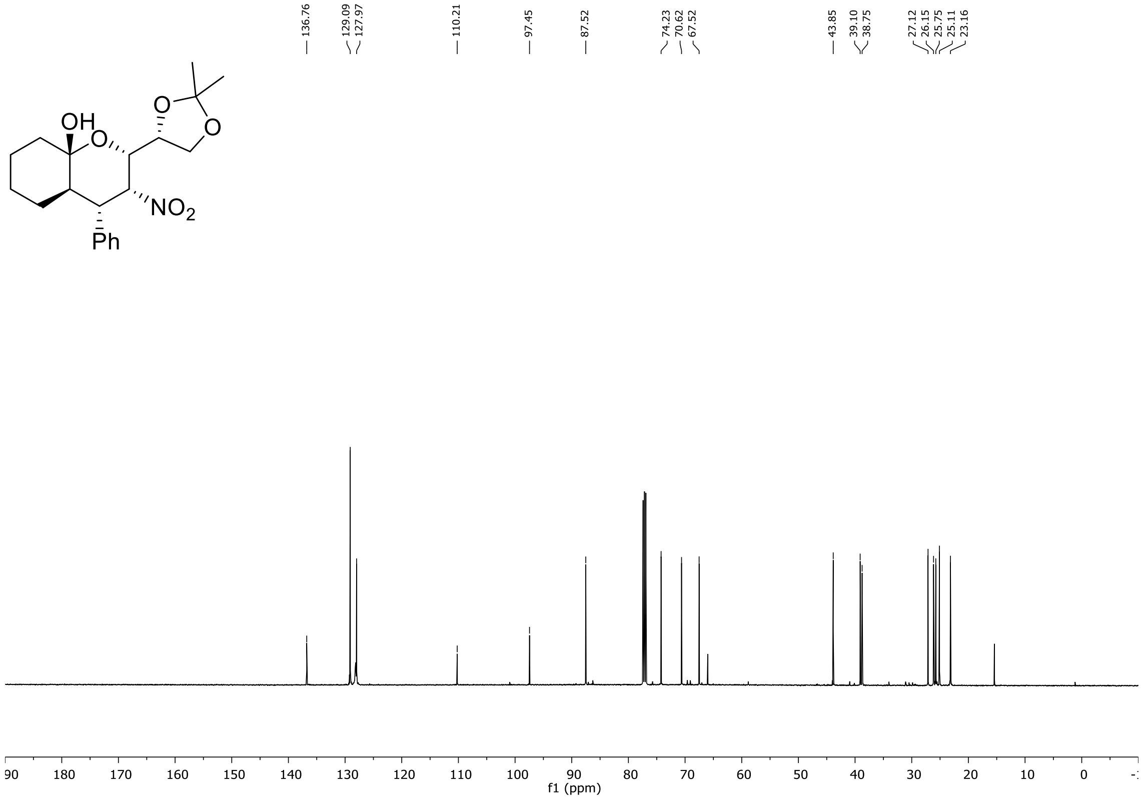

Compound **10caf**

^{1}H NMR (500 MHz, $CDCl_3$) of 10caf

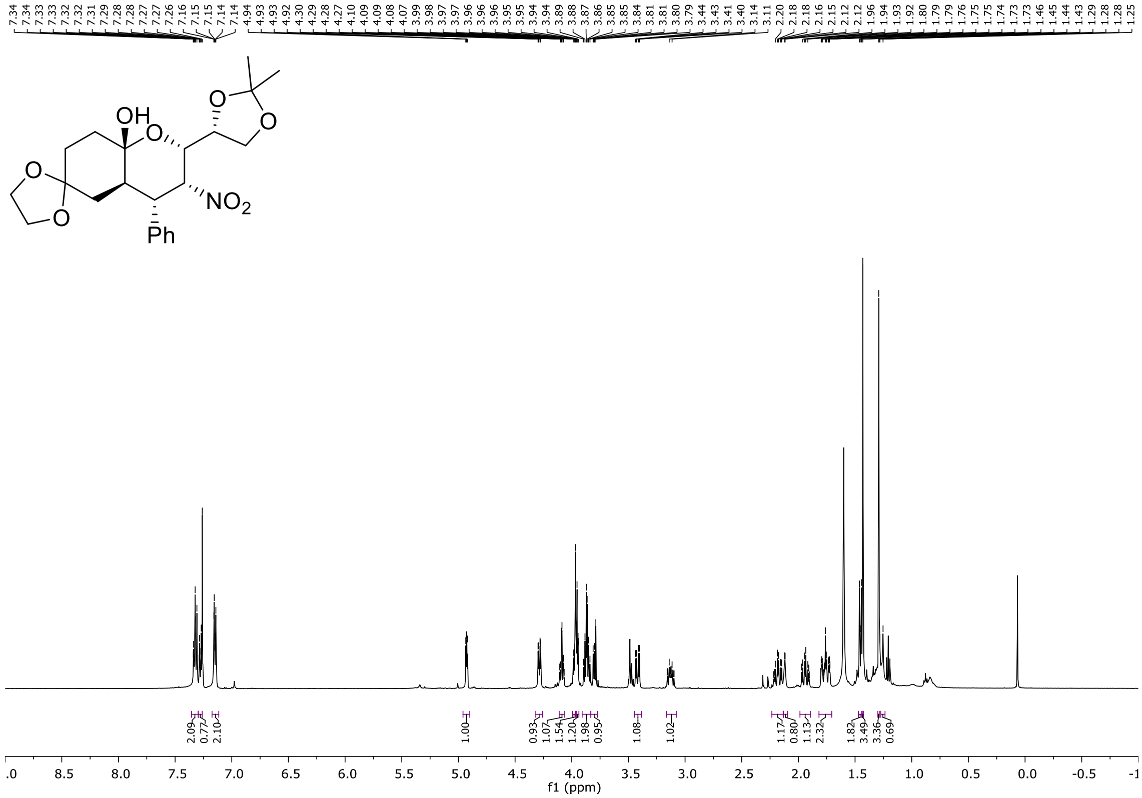

$^{13}C\{H\}$ NMR (126 MHz, $CDCl_3$) of 10caf

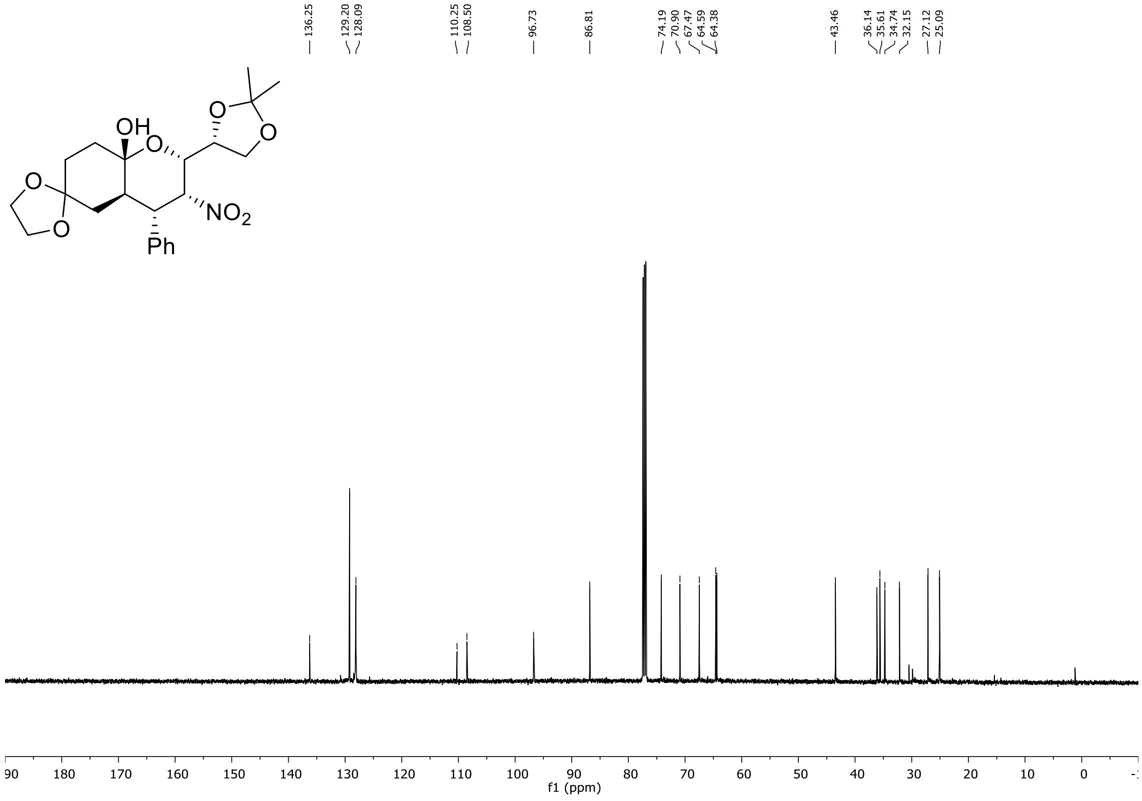

6 X-Ray diffraction structures

Compounds **10aaa** and **10aaa’** were recrystallized in a mixture of hexane and ethyl acetate. Crystal growth was performed by slow evaporation at room temperature of the solvent mixture.

6.1 X-Ray diffraction of 10aaa (CCDC 2090677)

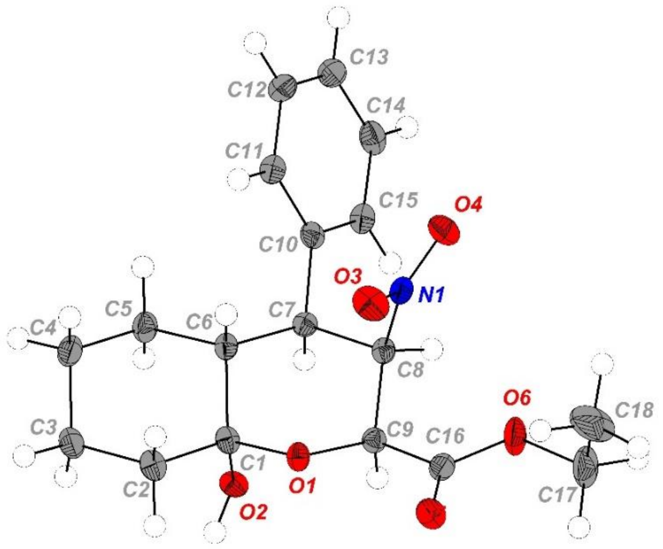

Figure S1. ORTEP diagram with thermal ellipsoids in 50% probability for (2*S*, 3*R*, 4*S*, 4a*R*, 8a*S*)-**10aaa.**

CheckCIF/PLATON report for (2*S*, 3*R*, 4*S*, 4a*R*, 8a*S*)-**10aaa.**

```
Bond precision:   C-C = 0.0051 A          Wavelength=1.54184

Cell:            a=12.2192(6)     b=5.5685(2)        c=12.6363(6)
                 alpha=90         beta=90.969(4)     gamma=90
Temperature:     100 K

                Calculated                 Reported
Volume          859.68(7)                  859.68(7)
Space group     P 21                       P 1 21 1
Hall group      P 2yb                      P 2yb
Moiety formula  C18 H23 N O6               C18 H23 N1 O6
Sum formula     C18 H23 N O6               C18 H23 N O6
Mr              349.37                     349.37
Dx,g cm-3       1.350                      1.350
Z               2                          2
Mu (mm-1)       0.845                      0.844
F000            372.0                      372.0
F000'           373.26
h,k,lmax        14,6,15                    14,6,15
Nref            3197[ 1777]                5436
Tmin,Tmax       0.769,0.888                0.501,1.000
Tmin'           0.582

Correction method= # Reported T Limits: Tmin=0.501 Tmax=1.000
AbsCorr = MULTI-SCAN

Data completeness= 3.06/1.70      Theta(max)= 68.909

R(reflections)= 0.0438( 5245)     wR2(reflections)= 0.1231( 5436)

S = 1.040                    Npar= 229
```

6.2 X-Ray diffraction of 10aaa’ (CCDC 2090834)

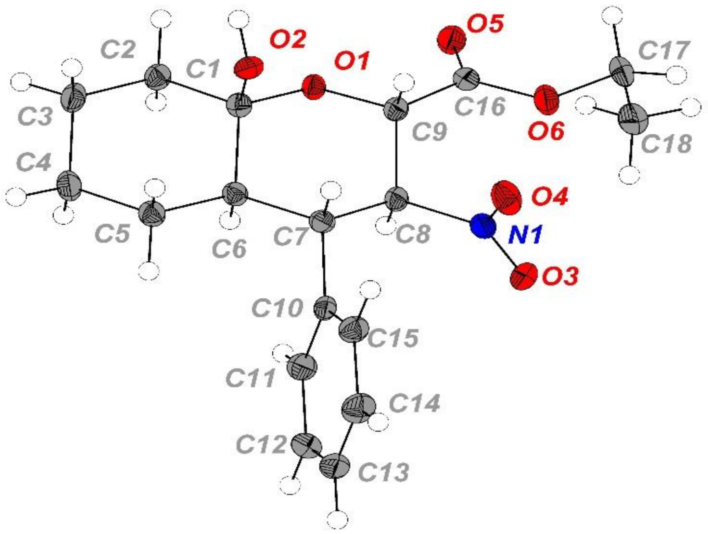

Figure S2. ORTEP diagram with thermal ellipsoids in 50% probability for (2*S*, 3*S*, 4*S*, 4a*R*, 8a*S*)-**10aaa’.**

CheckCIF/PLATON report for (2*S*, 3*S*, 4*S*, 4a*R*, 8a*S*)-**10aaa’**

```
Bond precision:   C-C = 0.0027 A          Wavelength=1.54184

Cell:             a=24.8420(5)    b=5.36209(11)       c=13.5597(2)
                  alpha=90        beta=93.6312(16)    gamma=90
Temperature:      100 K

                  Calculated                    Reported
Volume            1802.59(6)                    1802.59(6)
Space group       C 2                           C 1 2 1
Hall group        C 2y                          C 2y
Moiety formula    2(C18 H23 N O6), H2 O         C18 H23 N O6, 0.5(H2 O)
Sum formula       C36 H48 N2 O13                C18 H24 N O6.50
Mr                716.76                        358.38
Dx,g cm-3         1.321                         1.321
Z                 2                             4
Mu (mm-1)         0.839                         0.839
F000              764.0                         764.0
F000’             766.61
h,k,lmax          30,6,16                       30,6,17
Nref              3574[ 1988]                   3539
Tmin,Tmax         0.923,0.943                   0.677,1.000
Tmin’             0.686

Correction method= # Reported T Limits: Tmin=0.677 Tmax=1.000
AbsCorr = MULTI-SCAN

Data completeness= 1.78/0.99       Theta(max)= 72.374

R(reflections)= 0.0275( 3443)      wR2(reflections)= 0.0692( 3539)

S = 1.043                  Npar= 237
```

7 Computational studies

7.1 Michael-Henry-Hemiketalization Reaction

DFT calculations at the B3LYP-D3/6-31G(d) level of theory[15] were performed to understand the Henry-hemiketalization steps, for which no mechanistic studies are available. In these studies, solvent effects were tackled with the Continuum Polarization Method[16] (PCM, solvent=dichloromethane). All calculations were carried out with the Gaussian suite of programs.[17] Formation of adduct **10aaa** from cyclohexanone **6a**, *trans*-β-nitrostyrene **7a** and ethyl glyoxylate **8a** in the presence of trimethylamine and salicylic acid was chosen as model

system. The reaction profile obtained for this specific transformation is gathered in Figure S3.

Once Michael intermediate **9aaa** is generated, nitronate **INT1** is formed as a consequence of the interaction between **9aa** and trimethylamine, which is a suitable computational model of triethylamine. Nucleophilic addition of **INT1** on the aldehyde moiety of **8a** results in the formation of adduct **INT2** with a calculated activation energy of ca. 7 kcal/mol. All our attempts of connecting this latter alcohol adduct with hemiacetal **10aaa** met with no success. Instead, our calculations showed that participation of one equivalent of salicylic acid (SA) results in the activation of the nucleophilicity of the alcohol group of **INT2**, together with a slight enhancement of the electrophilicity of the ketone group (double HOMO-rising and LUMO-lowering activation). As a consequence, **10aaa** is formed from **INT2'** via **TS2**, with an activation energy similar to that found for the previous step via **TS1** (Figure S1). Reorganization of the acid and base additives leads to hemiacetal **10aaa** with a considerably exergonic thermodynamic balance from nitronate **INT1**. It is noteworthy that the C-O bond forming step takes place via an equatorial nucleophilic attack on the cyclohexanone moiety, which determines the (4*a*R,8*a*S) configuration of the *trans*-octahydro-2H-chromene moiety of **10aaa**, in which all the substituents occupy equatorial positions, with the only exception of the nitro group.

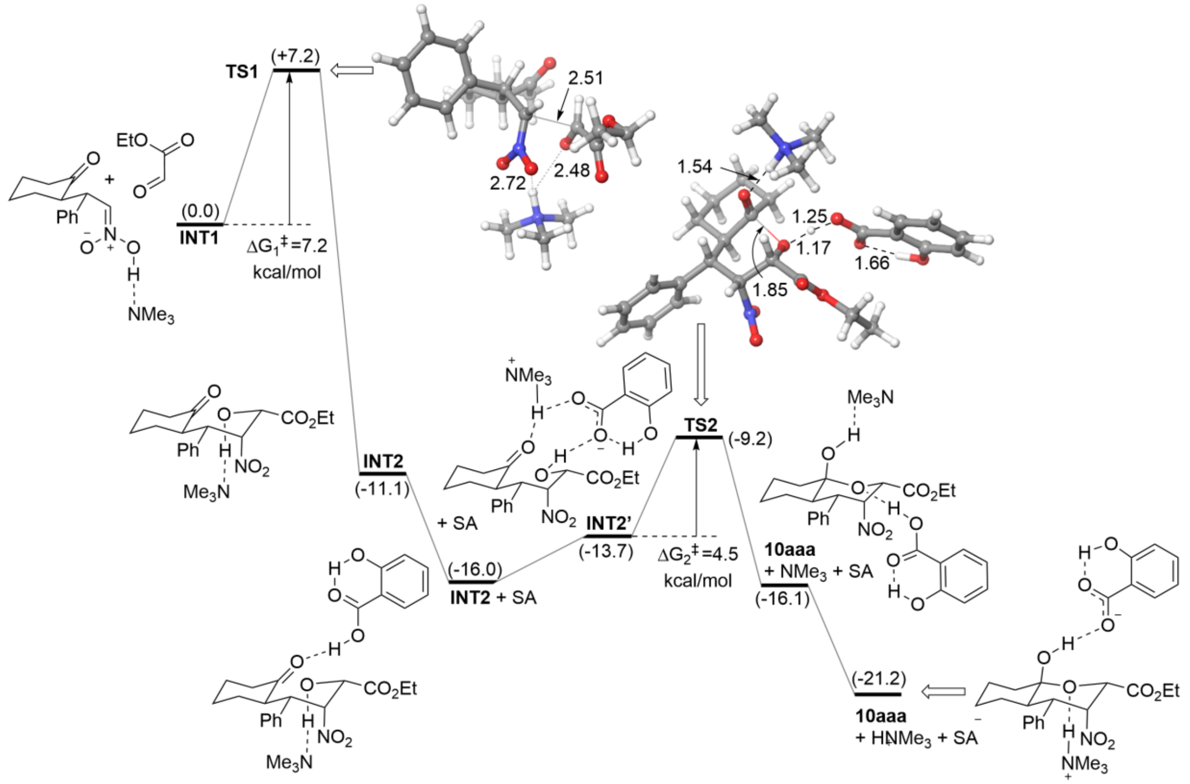

Figure S3. Reaction profile (B3LYP-D3(PCM)/6-31G(d) level of theory) associated with formation of bicyclic hemiacetal **10aaa** via nitronate intermediate **INT4** derived from **9aaa**. Trimethylamine has been used as a computational model of Et_3N. SA stands for salicylic acid. Calculations were performed in dichloromethane solution. Numbers in parentheses are relative Gibbs energies (298 K) with respect to **INT4** and are given in kcal/mol. Bond distances are given in Å.

7.2 Isomerization Reaction

This process is mediated by a suitable base such as DBU via nitronate **INTaad** shown in Figure S4. DFT calculations on these three local minima (B3LYP D3(PCM)/6 31G* level of theory[13-15] using acetonitrile as solvent) showed that all-equatorial isomer **10aad'** is ca. 4 kcal/mol more stable than **10aad**, the nitronate intermediate **INTaad** laying only ca. 2 kcal/mol above **10aad**. These results indicate that in the presence of DBU and a polar solvent such as acetonitrile the equilibrium is completely shifted towards the all-equatorial isomer, in nice agreement with our experimental findings.

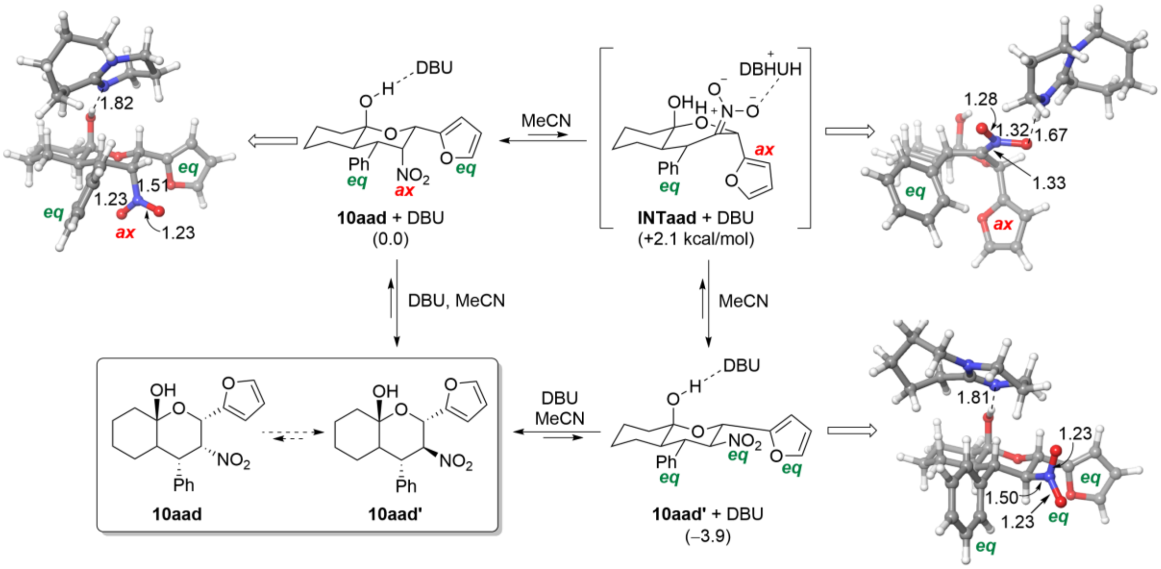

Figure S4. Reaction profile (B3LYP-D3(PCM,solvent=acetonitrile)/6-31G(d) level of theory) associated with isomerization of bicyclic hemiacetal **10aad** to all-equatorial diasteromer **10aad’** mediated by DBU. Numbers in parentheses are relative Gibbs energies (298 K), in kcal/mol. Optimized structures of both diasteremers and nitronate intermediate **INTaad** bound to DBU are also shown. Bond distances are given in Å.

Table S2.[a] Energies (E, in a. u.), Zero-Point Vibrational Energies (ZPVE, in a.u.), Thermal Corrections[b] for Gibbs Free Energies (TCGE, in a.u. at 298.15 K) and Number of Imaginary Frequencies[c] (NIMAG, imaginary wave numbers in cm^{-1}) of the Stationary Points Reported in Figures 3 and 4

Structure	E	ZPVE	TCGE	NIMAG
**INT1b**	-998.59667	0.41517	0.36108	0
**Ethyl glyoxalate b**	-381.70803	0.09996	0.06717	0
**TS1 b**	-1380.32681	0.52168	0.45880	1 (-46.74)
**INT2 b**	-1380.35850	0.52294	0.46133	0
**Salicylic acid (SA) b**	-496.06760	0.12042	0.08768	0
**INT2+SA b**	-1876.45725	0.64548	0.57238	0
**INT2' b**	-1876.45392	0.64782	0.57274	0
**TS2 b**	-1876.44136	0.64216	0.56743	1 (-707.28)
**10aaa+NMe3+SA b**	-1876.45874	0.64760	0.57388	0
**10aaa+N(+)HMe3+SA(-)b**	-1876.47380	0.65054	0.58079	0
**10aad+DBUc**	-1629.63603	0.62964	0.56430	0
**INTaad+DBUc**	-1629.62857	0.62873	0.56021	0
**10aad'+DBUc**	-1629.64177	0.62957	0.56386	0

[a]Calculations performed at the B3LYP-D3(PCM)/6-31(d) level of theory. [b]Results obtained using dichloromethane as solvent. [c]Results obtained using acetonitrile as solvent.

Cartesian coordinates of stationary points gathered in Figures 3 and 4

INT1

Center Number	Atomic Number	Atomic Type	Coordinates (Angstroms) X	Y	Z
1	6	0	-2.432174	-0.095978	0.235440
2	6	0	-4.140504	-0.138668	-1.659308
3	6	0	-4.529494	-1.579940	0.391841
4	6	0	-4.719130	-1.458622	-1.134768
5	6	0	-2.656370	-0.003970	-1.294811
6	1	0	-4.700659	0.704184	-1.229983
7	1	0	-5.129351	-0.802542	0.888132
8	1	0	-4.212394	-2.299506	-1.627673
9	1	0	-2.090497	-0.796770	-1.801743
10	1	0	-2.995765	0.738194	0.681911
11	1	0	-4.261802	-0.079176	-2.747394
12	1	0	-4.856238	-2.551860	0.772649
13	1	0	-5.784360	-1.542828	-1.377936
14	1	0	-2.252697	0.948803	-1.653060
15	6	0	-3.080429	-1.363062	0.794675
16	8	0	-2.483224	-2.140478	1.522027
17	6	0	-0.960193	0.060471	0.689937
18	6	0	-0.449048	1.469961	0.385175
19	6	0	0.392439	4.102901	-0.156090
20	6	0	0.293038	1.761336	-0.765998
21	6	0	-0.764947	2.517546	1.259790
22	6	0	-0.351005	3.823702	0.993776
23	6	0	0.714131	3.065783	-1.034303
24	1	0	0.554965	0.958821	-1.450021
25	1	0	-1.334799	2.305171	2.162080
26	1	0	-0.602201	4.621296	1.688125
27	1	0	1.295180	3.269593	-1.930061
28	1	0	0.721105	5.117814	-0.362728
29	1	0	-0.947176	-0.055896	1.781951
30	6	0	-0.082306	-1.017844	0.128906
31	1	0	-0.385378	-1.731502	-0.621612
32	7	0	1.134006	-1.137754	0.578343
33	8	0	1.685128	-0.405975	1.448886
34	8	0	1.882641	-2.183524	0.049213
35	1	0	2.835290	-1.763673	-0.068013
36	7	0	4.218317	-1.023318	-0.360201
37	6	0	5.029606	-1.791661	-1.312256
38	1	0	4.465232	-1.933726	-2.238864
39	1	0	5.257108	-2.774001	-0.887757
40	1	0	5.977045	-1.281750	-1.550280
41	6	0	4.897242	-0.869778	0.934137
42	1	0	4.212748	-0.378747	1.628618
43	1	0	5.822621	-0.278885	0.842857
44	1	0	5.149037	-1.858573	1.329368
45	6	0	3.831443	0.283912	-0.913845
46	1	0	3.163560	0.783003	-0.208100
47	1	0	3.294298	0.131732	-1.855302
48	1	0	4.708724	0.921604	-1.106957

Ethyl glyoxalate

Center Number	Atomic Number	Atomic Type	Coordinates (Angstroms) X	Y	Z
1	6	0	-1.383451	-1.495460	0.000000
2	1	0	-1.355234	-2.601259	0.000000
3	8	0	-2.404854	-0.851105	0.000000
4	6	0	0.022302	-0.880699	0.000000
5	8	0	1.010463	-1.588247	0.000000
6	8	0	0.000000	0.447424	0.000000
7	6	0	1.298458	1.111254	0.000000
8	1	0	1.849850	0.782572	0.886222
9	1	0	1.849850	0.782572	-0.886222
10	6	0	1.039975	2.603522	0.000000
11	1	0	1.995406	3.138316	0.000000
12	1	0	0.475779	2.900766	0.889344
13	1	0	0.475779	2.900766	-0.889344

TS1

Center Number	Atomic Number	Atomic Type	Coordinates (Angstroms) X	Y	Z
1	6	0	4.498099	-2.704792	0.298027
2	6	0	4.426048	-2.768300	1.690147
3	6	0	3.385042	-2.111466	2.354628
4	6	0	2.426023	-1.399351	1.634517
5	6	0	2.489610	-1.326598	0.232766
6	6	0	3.536004	-1.989145	-0.420822
7	6	0	1.452498	-0.551250	-0.582797
8	6	0	0.066723	-1.105731	-0.296663
9	7	0	-0.637766	-0.853598	0.822923
10	8	0	-0.393082	0.217971	1.514939
11	6	0	1.600065	0.999107	-0.504185
12	6	0	2.991151	1.509580	-0.032623
13	6	0	3.025805	3.042002	0.060341
14	6	0	2.668099	3.703673	-1.275955
15	6	0	1.306952	3.190173	-1.793712
16	6	0	1.319960	1.673625	-1.844096
17	8	0	1.196924	1.066291	-2.900021
18	6	0	-1.511599	0.389610	-1.551537
19	6	0	-2.669073	-0.579797	-1.354444
20	8	0	-2.511446	-1.654682	-2.139843
21	6	0	-3.447668	-2.757388	-1.991908
22	6	0	-2.799314	-3.850085	-1.157043
23	8	0	-1.482487	1.463713	-0.954581
24	8	0	-1.608358	-1.595020	1.179895
25	8	0	-3.611832	-0.369745	-0.615237
26	7	0	-2.691851	1.553576	1.991463
27	6	0	-2.001187	2.315176	3.073488
28	6	0	-3.524857	0.443793	2.549844
29	6	0	-3.478869	2.442965	1.087659
30	1	0	2.639481	4.795315	-1.178231

31	1	0	3.770302	1.155945	-0.722643
32	1	0	0.865098	1.382673	0.205827
33	1	0	0.508956	3.500087	-1.107543
34	1	0	2.310834	3.368960	0.829260
35	1	0	3.443357	3.470242	-2.018770
36	1	0	3.219240	1.073310	0.943735
37	1	0	1.079393	3.577501	-2.791234
38	1	0	4.018799	3.371261	0.390471
39	1	0	1.634018	-0.807213	-1.632267
40	1	0	3.598941	-1.945054	-1.505958
41	1	0	1.623564	-0.886354	2.155092
42	1	0	3.320292	-2.154342	3.439077
43	1	0	5.299996	-3.212961	-0.231648
44	1	0	5.171235	-3.324005	2.253220
45	1	0	-0.193359	-2.057500	-0.741480
46	1	0	-0.837823	0.150941	-2.384693
47	1	0	-4.365114	-2.382724	-1.533844
48	1	0	-3.661187	-3.087914	-3.011242
49	1	0	-2.508215	-3.448597	-0.182368
50	1	0	-1.904024	-4.235814	-1.656293
51	1	0	-3.501989	-4.679092	-1.016604
52	1	0	-1.915159	1.091259	1.456226
53	1	0	-2.860138	-0.241519	3.072950
54	1	0	-3.986438	-0.080837	1.716278
55	1	0	-4.274215	0.870167	3.219968
56	1	0	-2.810495	3.196807	0.673105
57	1	0	-4.276221	2.911782	1.668695
58	1	0	-3.876640	1.829375	0.282563
59	1	0	-1.375700	3.083331	2.616347
60	1	0	-1.376381	1.618220	3.631910
61	1	0	-2.747934	2.773869	3.724384

INT2

Center Number	Atomic Number	Atomic Type	Coordinates (Angstroms) X	Y	Z
1	1	0	-2.834640	-4.866030	-0.701660
2	6	0	-2.773320	-3.809082	-0.985210
3	6	0	-3.223872	-1.419595	-0.206078
4	6	0	-1.717174	-1.064731	-0.373732
5	6	0	-1.296878	-3.422649	-1.248303
6	6	0	-3.396629	-2.917168	0.098421
7	1	0	-3.765729	-1.152015	-1.124336
8	1	0	-1.199663	-1.401106	0.525608
9	1	0	-0.691915	-3.630603	-0.357165
10	1	0	-2.922386	-3.136739	1.065583
11	1	0	-3.336662	-3.695304	-1.921228
12	1	0	-3.659736	-0.824578	0.600113
13	1	0	-0.882892	-3.975216	-2.096283
14	1	0	-4.462113	-3.152206	0.208417
15	6	0	-1.257588	-1.939427	-1.543347
16	8	0	-1.060774	-1.501953	-2.668313
17	6	0	-1.419584	0.432012	-0.630306
18	1	0	-1.564781	0.576935	-1.707684
19	6	0	-2.329174	1.438016	0.069458

20	6	0	-3.953925	3.396568	1.285652
21	6	0	-2.867815	2.494817	-0.678041
22	6	0	-2.621189	1.379021	1.441401
23	6	0	-3.427273	2.347142	2.042429
24	6	0	-3.671149	3.467571	-0.079596
25	1	0	-2.655508	2.556314	-1.743155
26	1	0	-2.219011	0.571245	2.042556
27	1	0	-3.643898	2.279305	3.105186
28	1	0	-4.077688	4.276008	-0.681319
29	1	0	-4.581099	4.149353	1.755305
30	6	0	0.059058	0.858779	-0.434042
31	1	0	0.168103	1.887445	-0.776312
32	6	0	1.125864	-0.011583	-1.180016
33	1	0	0.769614	-0.045412	-2.215310
34	7	0	0.498754	0.920438	1.012695
35	8	0	1.221297	-1.315045	-0.714504
36	6	0	2.484310	0.708520	-1.193524
37	8	0	3.504214	0.257398	-0.714868
38	8	0	2.386073	1.889103	-1.822221
39	6	0	3.571567	2.734551	-1.834777
40	1	0	4.445927	2.107775	-2.026203
41	1	0	3.412601	3.402046	-2.683696
42	6	0	3.699055	3.498183	-0.526861
43	1	0	3.843622	2.807876	0.308125
44	1	0	2.798509	4.089944	-0.336876
45	1	0	4.559267	4.174563	-0.578153
46	1	0	1.748600	-1.374165	0.153405
47	7	0	2.612549	-1.910478	1.506329
48	6	0	3.184532	-0.851040	2.344774
49	1	0	2.377881	-0.237989	2.753591
50	1	0	3.822317	-0.215449	1.726816
51	1	0	3.780574	-1.264296	3.176511
52	6	0	3.665469	-2.699874	0.858135
53	1	0	3.206211	-3.455860	0.213322
54	1	0	4.313863	-3.208820	1.591281
55	1	0	4.271191	-2.036176	0.237071
56	6	0	1.694883	-2.762508	2.267633
57	1	0	1.236363	-3.494747	1.594332
58	1	0	0.903732	-2.144468	2.699053
59	1	0	2.207413	-3.305865	3.079788
60	8	0	1.304445	1.801659	1.311362
61	8	0	0.104471	0.059262	1.798008

Salicylic acid (SA)

Center Number	Atomic Number	Atomic Type	Coordinates (Angstroms) X	Y	Z
1	1	0	0.671710	-3.025229	0.000000
2	6	0	0.867643	-1.957824	0.000000
3	6	0	1.324179	0.801704	-0.000000
4	6	0	-0.228934	-1.081687	0.000000
5	6	0	2.162083	-1.456359	-0.000000
6	6	0	2.400224	-0.072033	-0.000000
7	6	0	0.000000	0.319190	-0.000000
8	1	0	2.999827	-2.148383	-0.000000

9	1	0	3.415922	0.310173	-0.000000
10	1	0	1.487097	1.873912	-0.000000
11	8	0	-1.463189	-1.623437	0.000000
12	1	0	-2.109859	-0.875138	0.000000
13	6	0	-1.152258	1.227696	-0.000000
14	8	0	-2.329970	0.857288	0.000000
15	8	0	-0.835410	2.534409	-0.000000
16	1	0	-1.673771	3.034466	-0.000000

INT2 + SA

Center Number	Atomic Number	Atomic Type	Coordinates (Angstroms) X	Y	Z
1	6	0	5.824560	1.606089	1.508781
2	6	0	6.499699	1.540121	0.288607
3	6	0	5.887174	0.920590	-0.803403
4	6	0	4.608377	0.375385	-0.678774
5	6	0	3.919539	0.434442	0.542708
6	6	0	4.547297	1.055292	1.631321
7	6	0	2.505452	-0.109958	0.717019
8	6	0	1.481159	0.965489	0.259992
9	7	0	1.604018	1.379416	-1.188856
10	8	0	1.860043	0.526905	-2.039321
11	6	0	2.263997	-1.534876	0.164395
12	6	0	3.432171	-2.541738	0.390693
13	6	0	3.109178	-3.893390	-0.269988
14	6	0	1.783824	-4.491508	0.227162
15	6	0	0.621144	-3.471506	0.097869
16	6	0	1.072590	-2.225146	0.814812
17	8	0	0.674810	-1.934839	1.944570
18	6	0	-0.011994	0.584214	0.487495
19	6	0	-0.936381	1.797637	0.341206
20	8	0	-0.660147	2.719045	1.273992
21	6	0	-1.454203	3.940207	1.239471
22	6	0	-0.944629	4.885187	0.163738
23	8	0	-0.442350	-0.491107	-0.281150
24	8	0	1.350827	2.554445	-1.451581
25	8	0	-1.814435	1.891167	-0.490878
26	7	0	-1.245741	-0.176839	-2.808482
27	6	0	-0.555690	-1.275692	-3.489318
28	6	0	-1.032736	1.099183	-3.500882
29	6	0	-2.675371	-0.460479	-2.638229
30	1	0	1.534392	-5.401427	-0.329841
31	1	0	3.596529	-2.670496	1.469483
32	1	0	2.076387	-1.485857	-0.908708
33	1	0	0.439140	-3.243834	-0.958138
34	1	0	3.054178	-3.748162	-1.358083
35	1	0	1.880981	-4.776945	1.282998
36	1	0	4.357140	-2.134236	-0.024523
37	1	0	-0.300478	-3.855366	0.541632
38	1	0	3.927513	-4.599579	-0.086572
39	1	0	2.319429	-0.170941	1.796091
40	1	0	4.029830	1.107119	2.586684
41	1	0	4.145698	-0.102462	-1.535269
42	1	0	6.405190	0.859867	-1.756741

43	1	0	6.291406	2.081068	2.367429
44	1	0	7.495213	1.964074	0.189845
45	1	0	1.680921	1.886360	0.808196
46	1	0	-0.058529	0.290877	1.538869
47	1	0	-2.499906	3.663180	1.086059
48	1	0	-1.333902	4.359537	2.240175
49	1	0	-1.074846	4.443435	-0.827441
50	1	0	0.117756	5.101203	0.314114
51	1	0	-1.502724	5.826963	0.205022
52	1	0	-0.698060	-0.217356	-1.232282
53	1	0	0.037582	1.317579	-3.536546
54	1	0	-1.530255	1.893360	-2.941251
55	1	0	-1.429687	1.075490	-4.530098
56	1	0	-2.797644	-1.395742	-2.085623
57	1	0	-3.197793	-0.549676	-3.605741
58	1	0	-3.130083	0.339960	-2.051469
59	1	0	-0.695837	-2.200577	-2.919872
60	1	0	0.513793	-1.054169	-3.537451
61	1	0	-0.934443	-1.435592	-4.513130
62	1	0	-1.062449	-2.223410	2.131323
63	8	0	-2.004291	-2.276484	1.811150
64	6	0	-2.548905	-1.063483	1.880229
65	8	0	-2.049517	-0.161316	2.570242
66	6	0	-3.769160	-0.872204	1.083656
67	6	0	-4.302074	-1.908123	0.294480
68	6	0	-5.423470	-1.700711	-0.495339
69	6	0	-6.025485	-0.432682	-0.512575
70	6	0	-5.519685	0.607284	0.256984
71	6	0	-4.393022	0.402638	1.067757
72	1	0	-3.810020	-2.874469	0.309637
73	1	0	-5.824823	-2.507576	-1.100212
74	1	0	-6.898467	-0.256915	-1.135347
75	1	0	-5.976038	1.591914	0.250919
76	8	0	-3.951408	1.442784	1.804717
77	1	0	-3.143419	1.126399	2.284114

INT2’

Center Number	Atomic Number	Atomic Type	Coordinates (Angstroms) X	Y	Z
1	6	0	3.166170	-0.575038	1.561582
2	6	0	3.798431	-0.197723	0.367536
3	6	0	5.098570	-0.695674	0.099256
4	6	0	5.715302	-1.563492	1.014672
5	6	0	5.058863	-1.925654	2.186736
6	6	0	3.778417	-1.430587	2.471096
7	6	0	3.101982	0.689821	-0.625654
8	8	0	3.724530	1.011674	-1.680701
9	8	0	1.919576	1.082952	-0.349512
10	8	0	-0.167832	-0.176942	-1.336903
11	6	0	-1.478864	1.858913	-0.375420
12	6	0	-2.735052	1.004753	-0.502388
13	6	0	-2.727732	-0.213612	0.449418
14	6	0	-1.743130	-1.348156	0.057193
15	6	0	-0.282742	-0.907875	-0.144616

16	6	0	-1.309982	2.848010	-1.503214
17	6	0	-2.472538	3.865496	-1.360675
18	6	0	-3.832991	3.150279	-1.316548
19	6	0	-3.882287	2.031427	-0.260185
20	8	0	-0.812942	1.921148	0.654850
21	6	0	0.677932	-2.103610	-0.035387
22	8	0	1.543585	-2.148372	-1.045617
23	6	0	2.599088	-3.144458	-0.960509
24	6	0	3.493253	-2.935795	-2.166310
25	7	0	-2.128863	-2.128565	-1.194123
26	8	0	-1.735785	-3.293014	-1.243951
27	6	0	-4.093405	-0.808377	0.778777
28	6	0	-4.383279	-1.134632	2.111142
29	6	0	-5.602513	-1.716649	2.463348
30	6	0	-6.560003	-1.978936	1.481761
31	6	0	-6.286299	-1.654831	0.150840
32	6	0	-5.064169	-1.077634	-0.198264
33	8	0	0.669733	-2.859986	0.917498
34	8	0	-2.747824	-1.574485	-2.097133
35	7	0	1.704115	3.500555	0.891629
36	6	0	0.638001	4.424475	1.383973
37	6	0	2.509213	4.110783	-0.214642
38	6	0	2.576854	3.029832	2.012703
39	1	0	-0.334118	3.336943	-1.438839
40	1	0	-2.810333	0.663134	-1.534883
41	1	0	-4.040030	2.711377	-2.302302
42	1	0	-3.784715	2.452685	0.750243
43	1	0	-2.328281	4.434792	-0.432148
44	1	0	-1.376901	2.333509	-2.467404
45	1	0	-4.628534	3.879142	-1.121821
46	1	0	-4.849616	1.524518	-0.300473
47	1	0	-2.437677	4.583847	-2.187042
48	1	0	-2.307690	0.148286	1.396117
49	1	0	-1.753787	-2.105981	0.841118
50	1	0	-0.037811	-0.283134	0.723591
51	1	0	2.137103	-4.136511	-0.947071
52	1	0	3.134649	-2.995873	-0.019032
53	1	0	3.913282	-1.926215	-2.165097
54	1	0	2.934046	-3.084084	-3.095715
55	1	0	4.320188	-3.653311	-2.139013
56	1	0	-4.865062	-0.833828	-1.235610
57	1	0	-3.644310	-0.930114	2.882958
58	1	0	-5.803686	-1.960055	3.503156
59	1	0	-7.025654	-1.851405	-0.620958
60	1	0	-7.511666	-2.428879	1.751060
61	1	0	1.266431	2.642031	0.496657
62	1	0	0.036137	3.898043	2.122475
63	1	0	0.007236	4.715965	0.545220
64	1	0	1.113427	5.303860	1.821937
65	1	0	3.179482	3.353350	-0.619305
66	1	0	3.065551	4.961259	0.182597
67	1	0	1.827134	4.443848	-0.998016
68	1	0	3.304804	2.324175	1.616736
69	1	0	1.948607	2.530892	2.751356
70	1	0	3.075882	3.891436	2.459163
71	1	0	0.662245	0.357953	-1.219561
72	8	0	5.759993	-0.376013	-1.033140

73	1	0	6.706273	-1.941085	0.781010
74	1	0	5.547334	-2.601194	2.884421
75	1	0	3.265861	-1.719873	3.383474
76	1	0	2.170949	-0.187395	1.751280
77	1	0	5.129691	0.241932	-1.523275

TS2

Center Number	Atomic Number	Atomic Type	Coordinates (Angstroms) X	Y	Z
1	6	0	-4.659446	0.135058	-0.945959
2	6	0	-4.286202	-0.194220	0.368751
3	6	0	-5.214504	-0.877664	1.194895
4	6	0	-6.485236	-1.199243	0.693256
5	6	0	-6.829369	-0.855364	-0.608506
6	6	0	-5.916988	-0.187863	-1.439644
7	6	0	-2.932906	0.123721	0.890637
8	8	0	-2.590538	-0.219507	2.041254
9	8	0	-2.129828	0.758729	0.076425
10	8	0	0.169360	0.113921	0.367968
11	6	0	1.284683	1.568231	0.595258
12	6	0	2.575028	0.777153	0.851851
13	6	0	2.979659	-0.013768	-0.409209
14	6	0	1.901890	-1.035630	-0.864147
15	6	0	0.481056	-0.420363	-0.887155
16	6	0	0.699090	2.148779	1.871256
17	6	0	1.726023	3.176293	2.394722
18	6	0	3.113886	2.537407	2.583957
19	6	0	3.614531	1.820501	1.315538
20	8	0	1.235044	2.264920	-0.475454
21	6	0	-0.565446	-1.429376	-1.375192
22	8	0	-1.147432	-2.070798	-0.362200
23	6	0	-2.193974	-3.021340	-0.700416
24	6	0	-2.683801	-3.617209	0.603850
25	7	0	1.849268	-2.293839	-0.019220
26	8	0	1.664824	-3.349878	-0.625782
27	6	0	4.352054	-0.671415	-0.369804
28	6	0	5.140512	-0.677240	-1.529106
29	6	0	6.388364	-1.302606	-1.548444
30	6	0	6.872270	-1.930864	-0.398851
31	6	0	6.098625	-1.927971	0.764138
32	6	0	4.848588	-1.306539	0.777963
33	8	0	-0.804212	-1.612158	-2.552668
34	8	0	1.937524	-2.205161	1.201116
35	7	0	-0.796823	3.662407	-1.323249
36	6	0	-0.113036	4.872160	-1.861974
37	6	0	-1.845152	4.012493	-0.321565
38	6	0	-1.343217	2.797788	-2.410360
39	1	0	-0.265270	2.623163	1.662006
40	1	0	2.385455	0.084771	1.673453
41	1	0	3.060727	1.805725	3.402987
42	1	0	3.775080	2.546925	0.507392
43	1	0	1.798456	3.995570	1.666699
44	1	0	0.530721	1.355312	2.606735
45	1	0	3.837469	3.302171	2.891658
46	1	0	4.576857	1.337118	1.512213

47	1	0	1.374095	3.611161	3.337488
48	1	0	2.985846	0.713720	-1.230373
49	1	0	2.142257	-1.408631	-1.860151
50	1	0	0.523557	0.363708	-1.656265
51	1	0	-1.773896	-3.773973	-1.374238
52	1	0	-2.984096	-2.480827	-1.230648
53	1	0	-3.062458	-2.837196	1.269328
54	1	0	-1.872624	-4.146920	1.113272
55	1	0	-3.492650	-4.327344	0.402769
56	1	0	4.255699	-1.317219	1.685786
57	1	0	4.772357	-0.184429	-2.426517
58	1	0	6.982577	-1.293852	-2.458303
59	1	0	6.467126	-2.411470	1.664923
60	1	0	7.844676	-2.415663	-0.408142
61	1	0	-0.016641	3.080286	-0.837020
62	1	0	0.682691	4.551606	-2.535865
63	1	0	0.316074	5.430853	-1.028522
64	1	0	-0.833962	5.493046	-2.398449
65	1	0	-2.268303	3.085629	0.066776
66	1	0	-2.619232	4.613342	-0.805286
67	1	0	-1.382285	4.584223	0.484539
68	1	0	-1.771967	1.905731	-1.952912
69	1	0	-0.526065	2.521035	-3.078300
70	1	0	-2.108222	3.348496	-2.962968
71	1	0	-1.011914	0.505017	0.322988
72	8	0	-4.914033	-1.252990	2.456317
73	1	0	-7.178371	-1.723313	1.343915
74	1	0	-7.815892	-1.113764	-0.984414
75	1	0	-6.189684	0.071156	-2.458112
76	1	0	-3.935204	0.643422	-1.573083
77	1	0	-3.977672	-0.940642	2.611663

10aaa + NMe_3 + SA

Center Number	Atomic Number	Atomic Type	Coordinates (Angstroms) X	Y	Z
1	6	0	5.078040	0.214738	0.183163
2	6	0	4.278038	-0.520115	-0.713550
3	6	0	4.848301	-1.613489	-1.415401
4	6	0	6.196557	-1.940340	-1.205596
5	6	0	6.964366	-1.196708	-0.318929
6	6	0	6.410610	-0.113774	0.382798
7	6	0	2.858025	-0.199539	-0.920023
8	8	0	2.124424	-0.824991	-1.695237
9	8	0	2.396369	0.815513	-0.184258
10	8	0	-0.249679	0.640140	0.073556
11	6	0	-1.218502	1.477694	-0.640655
12	6	0	-2.447673	0.617040	-0.978342
13	6	0	-3.045222	0.012264	0.308938
14	6	0	-1.971071	-0.705033	1.169472
15	6	0	-0.719294	0.186199	1.332470
16	6	0	-0.509681	1.937105	-1.915348
17	6	0	-1.481336	2.758593	-2.776478
18	6	0	-2.771218	1.978056	-3.076568
19	6	0	-3.442729	1.457171	-1.794857

20	8	0	-1.602274	2.536499	0.174209
21	6	0	0.420192	-0.516317	2.073494
22	8	0	1.054934	-1.367998	1.271413
23	6	0	2.125121	-2.180439	1.831133
24	6	0	2.311186	-3.353189	0.889154
25	7	0	-1.553368	-2.054541	0.615442
26	8	0	-1.503061	-2.984397	1.419456
27	6	0	-4.266620	-0.873141	0.111437
28	6	0	-5.329848	-0.780974	1.020408
29	6	0	-6.454138	-1.598635	0.897184
30	6	0	-6.533277	-2.523708	-0.146023
31	6	0	-5.482230	-2.621956	-1.060560
32	6	0	-4.357150	-1.805178	-0.932840
33	8	0	0.665216	-0.322076	3.245810
34	8	0	-1.244618	-2.142615	-0.568552
35	7	0	0.569484	3.955049	1.088773
36	6	0	-0.110948	5.160211	1.578112
37	6	0	1.620721	4.298299	0.123732
38	6	0	1.116320	3.168707	2.201380
39	1	0	0.367575	2.534408	-1.644402
40	1	0	-2.076379	-0.193794	-1.609066
41	1	0	-2.532223	1.122279	-3.724502
42	1	0	-3.791141	2.297477	-1.181538
43	1	0	-1.731081	3.679367	-2.234793
44	1	0	-0.150228	1.053276	-2.454671
45	1	0	-3.470839	2.611083	-3.635697
46	1	0	-4.319692	0.851437	-2.046651
47	1	0	-0.987848	3.055130	-3.709176
48	1	0	-3.347960	0.856079	0.941322
49	1	0	-2.373075	-0.943839	2.154118
50	1	0	-1.028508	1.032679	1.950996
51	1	0	1.835929	-2.492213	2.837759
52	1	0	3.017273	-1.550092	1.899221
53	1	0	2.585402	-3.012898	-0.112673
54	1	0	1.381447	-3.926640	0.819067
55	1	0	3.104163	-4.007426	1.265441
56	1	0	-3.548077	-1.897512	-1.649184
57	1	0	-5.276434	-0.058687	1.832071
58	1	0	-7.267563	-1.509215	1.612120
59	1	0	-5.535891	-3.336364	-1.877549
60	1	0	-7.407912	-3.160287	-0.247602
61	1	0	-0.775053	3.063126	0.416208
62	1	0	-0.919847	4.871294	2.256283
63	1	0	-0.544273	5.701752	0.731623
64	1	0	0.575261	5.836121	2.115411
65	1	0	2.110413	3.384537	-0.224455
66	1	0	2.387152	4.957119	0.566225
67	1	0	1.176074	4.810412	-0.735266
68	1	0	1.589688	2.262947	1.813730
69	1	0	0.308139	2.878115	2.879287
70	1	0	1.867430	3.734290	2.778051
71	1	0	1.401161	0.798255	-0.228278
72	8	0	4.140197	-2.376727	-2.273545
73	1	0	6.612577	-2.782077	-1.749813
74	1	0	8.007018	-1.462356	-0.167185
75	1	0	7.018226	0.459613	1.075513
76	1	0	4.628776	1.044065	0.718472

77	1	0	3.226201	-1.993070	-2.308970

10aaa + N(+)HMe$_3$ + SA(-)

Center Number	Atomic Number	Atomic Type	Coordinates (Angstroms) X	Y	Z
1	6	0	-6.215513	1.028281	-1.240996
2	6	0	-6.713723	0.975260	0.062602
3	6	0	-5.891003	0.521370	1.095955
4	6	0	-4.578717	0.126202	0.830199
5	6	0	-4.066957	0.176527	-0.475073
6	6	0	-4.903810	0.630296	-1.504012
7	6	0	-2.632136	-0.210840	-0.798863
8	6	0	-1.666189	0.984027	-0.589101
9	7	0	-1.736483	1.584461	0.798091
10	8	0	-1.639481	0.831796	1.773181
11	6	0	-2.127572	-1.502755	-0.120067
12	6	0	-2.960147	-2.744841	-0.475782
13	6	0	-2.410684	-3.986443	0.246853
14	6	0	-0.915533	-4.205729	-0.037783
15	6	0	-0.093659	-2.950112	0.293148
16	6	0	-0.651300	-1.755981	-0.479505
17	8	0	-0.543324	-1.934551	-1.854543
18	6	0	-0.204812	0.545984	-0.844711
19	6	0	0.789478	1.653528	-0.545977
20	8	0	0.686535	2.631008	-1.441153
21	6	0	1.522909	3.810685	-1.228279
22	6	0	0.903079	4.724687	-0.184788
23	8	0	0.131551	-0.582303	-0.067067
24	8	0	-1.820106	2.805437	0.884344
25	8	0	1.532956	1.654410	0.416837
26	7	0	1.187688	0.009288	2.712532
27	6	0	0.418064	-0.969877	3.540561
28	6	0	1.091606	1.386156	3.301385
29	6	0	2.612269	-0.414760	2.520383
30	1	0	-0.538159	-5.058433	0.539060
31	1	0	-2.926730	-2.897148	-1.561381
32	1	0	-2.153034	-1.367962	0.964637
33	1	0	-0.155816	-2.742484	1.366501
34	1	0	-2.555704	-3.859426	1.330220
35	1	0	-0.776288	-4.444799	-1.098716
36	1	0	-4.007935	-2.579557	-0.201600
37	1	0	0.959710	-3.070846	0.023343
38	1	0	-2.985019	-4.874031	-0.045378
39	1	0	-2.568247	-0.383832	-1.880062
40	1	0	-4.523219	0.669614	-2.522165
41	1	0	-3.952173	-0.219298	1.645517
42	1	0	-6.270229	0.473113	2.113204
43	1	0	-6.847513	1.374449	-2.054303
44	1	0	-7.735201	1.281339	0.270994
45	1	0	-1.924545	1.813758	-1.245662
46	1	0	-0.126879	0.312818	-1.909211
47	1	0	2.522629	3.469116	-0.954144
48	1	0	1.558408	4.281182	-2.212226
49	1	0	0.863181	4.229352	0.788977

50	1	0	-0.113639	5.007527	-0.473925
51	1	0	1.506370	5.634100	-0.090308
52	1	0	0.768230	0.032878	1.765048
53	1	0	0.044048	1.686605	3.294844
54	1	0	1.678408	2.065782	2.688887
55	1	0	1.478116	1.349386	4.321097
56	1	0	2.624265	-1.404572	2.067241
57	1	0	3.105450	-0.434156	3.493482
58	1	0	3.096147	0.291574	1.848001
59	1	0	0.532044	-1.963948	3.111499
60	1	0	-0.631195	-0.676113	3.538543
61	1	0	0.817570	-0.957307	4.555605
62	1	0	0.431335	-2.082256	-2.055698
63	8	0	2.090468	-2.216193	-2.003586
64	6	0	2.580185	-1.053959	-1.932920
65	8	0	2.141559	-0.043320	-2.580917
66	6	0	3.738384	-0.817984	-0.997267
67	6	0	4.243909	-1.860028	-0.210110
68	6	0	5.279192	-1.651916	0.698788
69	6	0	5.825828	-0.367780	0.827644
70	6	0	5.351695	0.685263	0.049723
71	6	0	4.315891	0.471347	-0.874488
72	1	0	3.794074	-2.841070	-0.327871
73	1	0	5.656708	-2.473420	1.301023
74	1	0	6.630103	-0.187138	1.536739
75	1	0	5.770780	1.683803	0.133999
76	8	0	3.897184	1.507532	-1.631066
77	1	0	3.132381	1.114968	-2.173513

10aad+DBU

Center Number	Atomic Number	Atomic Type	Coordinates (Angstroms) X	Y	Z
1	6	0	-1.539675	-0.272892	2.073957
2	6	0	-1.204472	-1.645582	4.160444
3	6	0	0.093881	-2.187723	2.040786
4	6	0	-0.540579	-2.750785	3.323084
5	6	0	-0.957855	-1.423360	1.220224
6	6	0	-2.208168	-0.831868	3.330661
7	1	0	-0.431862	-0.964602	4.537447
8	1	0	0.915600	-1.512355	2.307022
9	1	0	-1.295702	-3.500691	3.045350
10	1	0	-1.798391	-2.097205	1.034673
11	1	0	-3.055515	-1.457054	3.024986
12	1	0	-1.707772	-2.079227	5.032930
13	1	0	0.524115	-2.996376	1.440362
14	1	0	0.218002	-3.272165	3.919733
15	1	0	-2.606589	0.010818	3.906777
16	8	0	-0.586330	0.669055	2.488592
17	6	0	-0.456451	-0.891931	-0.138397
18	1	0	0.338237	-0.163855	0.061529
19	6	0	-1.529413	-0.026618	-0.845948
20	1	0	-1.083722	0.515266	-1.680036
21	6	0	-2.155298	0.995176	0.139282
22	1	0	-1.337864	1.694017	0.356967

23	6	0	-3.259127	1.804330	-0.445817
24	6	0	-3.332159	3.111400	-0.825762
25	1	0	-2.544447	3.845845	-0.728036
26	6	0	-4.645593	3.304698	-1.370968
27	1	0	-5.062216	4.218901	-1.769967
28	6	0	-5.272791	2.099789	-1.280238
29	1	0	-6.258885	1.748961	-1.544780
30	8	0	-4.443150	1.170303	-0.719798
31	6	0	0.118305	-1.926291	-1.091983
32	6	0	1.206803	-3.749360	-2.941001
33	6	0	-0.396440	-3.225388	-1.200669
34	6	0	1.178259	-1.553341	-1.930797
35	6	0	1.721722	-2.453951	-2.847437
36	6	0	0.145933	-4.130380	-2.116055
37	1	0	-1.222807	-3.535552	-0.569500
38	1	0	1.580541	-0.545355	-1.862046
39	1	0	2.547930	-2.145439	-3.482208
40	1	0	-0.262105	-5.135297	-2.182923
41	1	0	1.629150	-4.456123	-3.649908
42	7	0	-2.599759	-0.865524	-1.499977
43	8	0	-3.215912	-1.679987	-0.814811
44	8	0	-2.806061	-0.674948	-2.697750
45	1	0	0.217446	0.747543	1.904976
46	7	0	1.600345	1.552715	1.040036
47	6	0	2.752363	1.230405	0.517585
48	6	0	1.152762	2.929754	0.844800
49	1	0	1.705278	3.606393	1.516677
50	1	0	0.098596	2.996574	1.129869
51	6	0	1.384957	3.354864	-0.603602
52	1	0	0.843480	2.672099	-1.269835
53	1	0	1.026669	4.370416	-0.801282
54	6	0	2.884252	3.280499	-0.862552
55	1	0	3.095462	3.265898	-1.939841
56	1	0	3.384220	4.165964	-0.446433
57	6	0	3.233541	-0.185422	0.818770
58	1	0	2.325515	-0.784388	0.903911
59	1	0	3.684031	-0.192782	1.820498
60	6	0	4.186820	-0.838080	-0.190465
61	1	0	3.868472	-0.581868	-1.207582
62	1	0	4.093310	-1.927079	-0.113233
63	6	0	5.647470	-0.422980	0.023164
64	1	0	6.239178	-0.704757	-0.856982
65	1	0	6.069942	-0.969127	0.876184
66	6	0	5.779759	1.085359	0.275859
67	1	0	6.822041	1.393088	0.134304
68	1	0	5.527044	1.330801	1.314628
69	6	0	4.908797	1.934752	-0.651254
70	1	0	4.974535	1.563792	-1.685155
71	1	0	5.308405	2.955759	-0.664497
72	7	0	3.493390	2.076921	-0.264460
73	8	0	-2.600142	0.375567	1.331540

INTaad+DBU

Center Number	Atomic Number	Atomic Type	Coordinates (Angstroms) X	Y	Z

1	6	0	-2.782125	-1.960714	-1.067130
2	6	0	-4.832865	-2.536686	-2.457864
3	6	0	-4.140901	-0.122964	-2.167789
4	6	0	-5.322648	-1.087907	-2.329769
5	6	0	-3.250557	-0.491217	-0.970478
6	6	0	-3.960254	-2.917762	-1.256969
7	1	0	-4.247087	-2.645120	-3.379490
8	1	0	-3.526957	-0.146444	-3.078319
9	1	0	-5.983852	-1.004123	-1.454643
10	1	0	-3.842431	-0.421836	-0.050229
11	1	0	-4.554216	-2.887833	-0.335914
12	1	0	-5.681864	-3.227117	-2.532430
13	1	0	-4.499484	0.905659	-2.049790
14	1	0	-5.919736	-0.806036	-3.205993
15	1	0	-3.556339	-3.931462	-1.356930
16	8	0	-1.916247	-2.153289	-2.184117
17	6	0	-2.026132	0.456701	-0.838718
18	1	0	-1.608616	0.606149	-1.844677
19	6	0	-1.264883	-1.465332	0.803080
20	1	0	-0.311214	-1.974458	0.967149
21	6	0	-1.823620	-1.154450	2.167102
22	6	0	-1.214439	-0.873657	3.355310
23	1	0	-0.149973	-0.909561	3.537277
24	6	0	-2.255167	-0.497929	4.271720
25	1	0	-2.145243	-0.214898	5.309726
26	6	0	-3.421878	-0.580129	3.575676
27	1	0	-4.458989	-0.418523	3.827670
28	8	0	-3.177522	-0.978412	2.287663
29	6	0	-2.437649	1.840710	-0.337802
30	6	0	-3.300449	4.378317	0.543365
31	6	0	-2.583665	2.114321	1.029059
32	6	0	-2.723255	2.862551	-1.253078
33	6	0	-3.152688	4.119782	-0.821275
34	6	0	-3.009882	3.369746	1.466289
35	1	0	-2.343983	1.348163	1.758155
36	1	0	-2.601584	2.672068	-2.316998
37	1	0	-3.365447	4.897137	-1.550895
38	1	0	-3.112879	3.559749	2.531872
39	1	0	-3.631347	5.355816	0.884200
40	1	0	-1.125898	-1.608564	-2.026319
41	8	0	-2.143326	-2.386940	0.131696
42	6	0	-0.978754	-0.222083	0.000625
43	7	0	0.223611	0.333353	0.099119
44	8	0	1.126467	-0.181335	0.905688
45	8	0	0.527619	1.384015	-0.569771
46	1	0	2.548397	0.512732	0.357095
47	7	0	3.477336	0.846845	0.000055
48	6	0	4.348153	-0.060543	-0.388430
49	7	0	5.531859	0.267824	-0.923524
50	6	0	5.920656	1.683315	-1.051710
51	1	0	6.602005	1.764121	-1.902069
52	1	0	6.466470	1.992184	-0.151061
53	6	0	4.682243	2.552846	-1.265466
54	1	0	4.236843	2.316287	-2.238165
55	1	0	4.970873	3.607305	-1.273934
56	6	0	3.668183	2.288193	-0.156268

57	1	0	2.685647	2.707609	-0.382730
58	1	0	4.012465	2.709672	0.796090
59	6	0	3.996897	-1.515400	-0.199437
60	1	0	4.119676	-2.018655	-1.163430
61	1	0	2.935986	-1.569753	0.054595
62	6	0	4.857517	-2.216770	0.898441
63	1	0	5.074363	-3.238703	0.568115
64	1	0	4.250608	-2.300629	1.805992
65	6	0	6.165984	-1.497294	1.268604
66	1	0	5.921014	-0.508573	1.678963
67	1	0	6.650747	-2.046357	2.083900
68	6	0	7.173167	-1.326510	0.119422
69	1	0	7.637801	-2.289309	-0.126370
70	1	0	7.977666	-0.659981	0.451513
71	6	0	6.541387	-0.775216	-1.173864
72	1	0	7.303084	-0.337480	-1.821895
73	1	0	6.073661	-1.581216	-1.744714

10aad’+DBU

Center Number	Atomic Number	Atomic Type	Coordinates (Angstroms) X	Y	Z
1	6	0	1.099071	-2.232123	0.033450
2	6	0	-0.061775	-4.243369	1.012547
3	6	0	-0.696148	-1.903851	1.802376
4	6	0	-0.574497	-3.388378	2.181762
5	6	0	0.628343	-1.377333	1.236182
6	6	0	1.258918	-3.693878	0.454868
7	1	0	-0.807608	-4.249340	0.208553
8	1	0	-1.488583	-1.769515	1.062837
9	1	0	0.120877	-3.482079	3.028762
10	1	0	1.394403	-1.514522	2.012382
11	1	0	2.050627	-3.754088	1.211019
12	1	0	0.075192	-5.282743	1.334221
13	1	0	-0.980240	-1.312355	2.678962
14	1	0	-1.545566	-3.763035	2.528291
15	1	0	1.589175	-4.263968	-0.420429
16	8	0	0.239408	-2.220279	-1.069622
17	6	0	0.622696	0.125507	0.859726
18	1	0	-0.018355	0.260617	-0.016671
19	6	0	2.066530	0.454570	0.437163
20	6	0	2.499317	-0.435037	-0.757786
21	1	0	1.822480	-0.249447	-1.600332
22	6	0	3.883515	-0.132698	-1.208026
23	6	0	4.397343	0.309522	-2.390138
24	1	0	3.836270	0.497116	-3.295139
25	6	0	5.808263	0.473640	-2.185494
26	1	0	6.541241	0.807214	-2.906565
27	6	0	6.045383	0.122272	-0.891585
28	1	0	6.935124	0.075751	-0.281913
29	8	0	4.883552	-0.247385	-0.275764
30	6	0	0.086395	1.020560	1.959696
31	6	0	-0.998021	2.634671	3.993524
32	6	0	0.810149	1.271123	3.133514
33	6	0	-1.189435	1.585224	1.822820

34	6	0	-1.730379	2.385739	2.830723
35	6	0	0.273548	2.073859	4.141747
36	1	0	1.797740	0.838269	3.267954
37	1	0	-1.766395	1.388742	0.922901
38	1	0	-2.721169	2.813880	2.705127
39	1	0	0.849413	2.260734	5.044046
40	1	0	-1.413817	3.259758	4.778857
41	1	0	-0.245317	-1.367702	-1.269699
42	7	0	-1.360763	-0.134955	-1.981010
43	6	0	-2.604814	-0.097508	-1.583426
44	6	0	-0.830220	1.035774	-2.676881
45	1	0	-1.116849	0.988470	-3.739548
46	1	0	0.260849	1.002854	-2.636738
47	6	0	-1.348336	2.332317	-2.062016
48	1	0	-0.981646	2.411812	-1.034124
49	1	0	-0.984924	3.210579	-2.605744
50	6	0	-2.869377	2.309379	-2.085203
51	1	0	-3.270805	3.069591	-1.403977
52	1	0	-3.246632	2.544074	-3.090382
53	6	0	-3.138775	-1.402223	-1.013067
54	1	0	-2.257252	-2.009096	-0.804369
55	1	0	-3.674264	-1.936265	-1.809208
56	6	0	-4.035990	-1.275347	0.228736
57	1	0	-3.682731	-0.437623	0.843846
58	1	0	-3.927048	-2.172119	0.849295
59	6	0	-5.514253	-1.077119	-0.139956
60	1	0	-6.056431	-0.669574	0.722538
61	1	0	-5.971705	-2.047924	-0.369232
62	6	0	-5.694417	-0.148701	-1.349553
63	1	0	-6.741933	0.169109	-1.409317
64	1	0	-5.485173	-0.677769	-2.286489
65	6	0	-4.838788	1.116934	-1.295275
66	1	0	-4.905394	1.572437	-0.295190
67	1	0	-5.264503	1.844719	-1.995564
68	7	0	-3.416526	1.001559	-1.675053
69	8	0	2.429383	-1.793587	-0.343878
70	1	0	2.771748	0.314896	1.255369
71	7	0	2.199622	1.903085	0.058910
72	8	0	1.557768	2.299716	-0.914142
73	8	0	2.943313	2.608980	0.735051

----

8 References

[1] a) Tsakos, M.; Trifonidou, M.; Kokotos, C. G. *Tetrahedron* **2012**, 8630-8635. b) Tsakos, M.; Kokotos, C. G.Eur. *J. Org. Chem.* **2012**, 576-580.

[2] Leyes, A. E.; Poulter, C. D. *Org. Lett*. **1999**, *1*, 1067-1070.

[3] Conde, E.; Bello, D.; de Cózar, A.; Sanchez, M.; Vazquez, M. A.; Cossío, F. P. *Chem. Sci.* **2012**, *3*, 1486–1491.

[4] Retamosa, M. G.; de Cózar, A.; Sánchez, M.; Miranda, J. I.; Sansano, J. M.; Castelló, L. M.; Nájera, C.; Jiménez, A. I.; Sayago, F. J.; Cativiela, C.; Cossío, F. P. *Eur. J. Org. Chem*. **2015**, 2503-2516.

[5] Retamosa, M. G.; Ruiz-Olalla, A.; Bello, T.; de Cózar, A.; Cossío, F. P. *Angew. Chem., Int. Ed*. **2018**, *57*, 668-672.

[6] Retamosa, M. G.; Ruiz-Olalla, A.; Agirre, M.; de Cózar, A.; Bello, T.; Cossío, F. P. *Chem. Eur. J*. **2021**, *27*, 15671–15687.

[7] CrysAlisPro, Agilent Technologies, Version 1.171.37.31.

[8] Palatinus, L.; Chapuis, G. *J. Appl. Cryst*. **2007**, *40*, 786-790.

[9] a) Sheldrick, G. M. *Acta Cryst*. **2008**, A64, 112-122. b) Sheldrick, G. M. *Acta Cryst*. **2015**, C71, 3-8.

[10] Macrae, C. F. *J. Appl. Cryst*. **2008**, *41*, 466-470.

[11] a) Spek, A. L. *PLATON, A Multipurpose Crystallographic Tool*, Utrecht University, The Nederlands **2010**. b) Spek, A. L. *J. Appl. Cryst*. **2003**, *36*, 7-13.

[12] Farrugia, L. J. *J. Appl. Cryst*. **1999**, *32*, 837-838.

[13] Enders, D; Seki, A. Proline-Catalyzed Enantioselective Michael Additions of Ketones to Nitrostyrene. *Synlett* **2002**, 26-28.

[14] Ruiz-Olalla, A.; Retamosa, M. G.; Cossío, F. P. Densely Substituted l-Proline Esters as Catalysts for Asymmetric Michael Additions of Ketones to Nitroalkenes. *J. Org. Chem*. **2015**, *80*, 5588-5599.

[15](a) Becke, A. D. *J. Chem. Phys.* **1993**, *98*, 5648-5652. (b) Lee, C.; Yang, W.; Parr, R. G. *Phys. Rev. B* **1988**, *37*, 785-789. (c) Vosko, S. H.; Wilk, L.; Nusair, M. *Can. J. Phys.* **1980**, *58*, 1200-1211. (d) Grimme, S.; Antony, J.; Enrlich, S.; Krieg, S. *J. Chem. Phys.* **2010**, *132*, 154104. (e) Jensen, F. *WIREs Computational Molecular Science* **2013**, *3*, 273-295.

[16] (a) Cammi, R.; Mennucci, B.; Tomasi, J. *J. Am. Chem. Soc*. **1998**, *120*, 8834-8847. (b) Tomasi, J.; Mennucci, B.; Cammi, R. *Chem. Rev.* **2005**, *105*, 2999-3094.

[17] Frisch, M. J.; Trucks, G. W.; Schlegel, H. B.; Scuseria, G. E.; Robb, M. A.; Cheeseman, J. R.; Scalmani, G.; Barone, V.; Petersson, G. A.; Nakatsuji, H.; Li, X.; Caricato, M.; Marenich, A. V.; Bloino, J.; Janesko, B. G.; Gomperts, R.; Mennucci, B.; Hratchian, H. P.; Ortiz, J. V.; Izmaylov, A. F.; Sonnenberg, J. L.; Williams; Ding, F.; Lipparini, F.; Egidi, F.; Goings, J.; Peng, B.; Petrone, A.; Henderson, T.; Ranasinghe, D.; Zakrzewski, V. G.; Gao, J.; Rega, N.; Zheng, G.; Liang, W.; Hada, M.; Ehara, M.; Toyota, K.; Fukuda, R.; Hasegawa, J.; Ishida,

M.; Nakajima, T.; Honda, Y.; Kitao, O.; Nakai, H.; Vreven, T.; Throssell, K.; Montgomery Jr., J. A.; Peralta, J. E.; Ogliaro, F.; Bearpark, M. J.; Heyd, J. J.; Brothers, E. N.; Kudin, K. N.; Staroverov, V. N.; Keith, T. A.; Kobayashi, R.; Normand, J.; Raghavachari, K.; Rendell, A. P.; Burant, J. C.; Iyengar, S. S.; Tomasi, J.; Cossi, M.; Millam, J. M.; Klene, M.; Adamo, C.; Cammi, R.; Ochterski, J. W.; Martin, R. L.; Morokuma, K.; Farkas, O.; Foresman, J. B.; Fox, D. J.: Gaussian 16 Rev. C.01. Wallingford, CT, **2016**.
